# The genome evolution and low-phosphorus adaptation in white lupin

**DOI:** 10.1038/s41467-020-14891-z

**Published:** 2020-02-26

**Authors:** Weifeng Xu, Qian Zhang, Wei Yuan, Feiyun Xu, Mehtab Muhammad Aslam, Rui Miao, Ying Li, Qianwen Wang, Xing Li, Xin Zhang, Kang Zhang, Tianyu Xia, Feng Cheng

**Affiliations:** 1 Center for Plant Water-use and Nutrition Regulation and College of Life Sciences, Joint International Research Laboratory of Water and Nutrient in Crop, Fujian Agriculture and Forestry University, Jinshan, Fuzhou 350002 China; 2 Institute of Vegetables and Flowers, Chinese Academy of Agricultural Sciences, Key Laboratory of Biology and Genetic Improvement of Horticultural Crops of the Ministry of Agriculture, Sino-Dutch Joint Laboratory of Horticultural Genomics, Beijing, China

**Keywords:** Genome evolution, Agricultural genetics, Polyploidy in plants

## Abstract

White lupin (*Lupinus albus*) is a legume crop that develops cluster roots and has high phosphorus (P)-use efficiency (PUE) in low-P soils. Here, we assemble the genome of white lupin and find that it has evolved from a whole-genome triplication (WGT) event. We then decipher its diploid ancestral genome and reconstruct the three sub-genomes. Based on the results, we further reveal the sub-genome dominance and the genic expression of the different sub-genomes varying in relation to their transposable element (TE) density. The PUE genes in white lupin have been expanded through WGT as well as tandem and dispersed duplications. Furthermore, we characterize four main pathways for high PUE, which include carbon fixation, cluster root formation, soil-P remobilization, and cellular-P reuse. Among these, auxin modulation may be important for cluster root formation through involvement of potential genes *LaABCG36*s and *LaABCG37*s. These findings provide insights into the genome evolution and low-P adaptation of white lupin.

## Abstract (web summary)

White lupin is a legume crop that develops cluster roots for efficient phosphate acquisition. Here, the authors assemble its triplicated genome, reveal gene expression difference among sub-genomes, and characterize possible pathways related to high phosphorus-use efficiency.

## Introduction

Phosphorus (P) is required for plant growth. P is often present in the soil in unavailable forms, such as phytic acid, or calcium (Ca), iron (Fe), and aluminum (Al) phosphates^1^, and these forms are difficult for plants to absorb. About 5.7 billion hectares of land worldwide contain too little P for crop growth, and P deficiency constrains agricultural productivity^2^. To cope with P-limiting environments, several processes have evolved in plants. These include morphological, physiological, biochemical, and molecular adaptations^3^.

White lupin (*Lupinus albus*, 2*n* = 50) belonging to the family Fabaceae can mobilize soil phosphates by the formation of densely packed lateral roots. These are also called cluster roots or proteoid roots. White lupin can grow without the addition of P fertilizer or help from mycorrhizal fungi, and its ability to fix dinitrogen (N_2_) is less inhibited by P deficiency compared to other legumes^2^. The morphological and physiological factors of low-P adaptation in white lupin have been well studied^4^. However, the genome evolution and low-P adaptation in white lupin is unclear.

All plant species have experienced polyploidization events^5^, which are involved in plant speciation and evolution of new functions. Narrow-leaf lupin (*Lupinus angustifolius*) is a close relative of white lupin. *Lupinus angustifolius* experienced a hexaploidization event^6^ that was shared with white lupin. Based on genomic block (GB) systems^7^, diploid ancestors of paleopolyploids have been found in *Brassica*^8^ and in the Poaceae^9^. After allopolyploidization events, sub-genome dominance phenomena are important features shaping the evolution of polyploid genomes. Sub-genome dominance indicates that one sub-genome has a higher gene density than the others. In addition, there are more genes from this sub-genome expressed at higher levels than their paralogs from the other sub-genomes^10,11^. Transposable elements (TEs) have been shown to play a role in dominant expression of paralogous genes between sub-genomes in *Brassica rapa* and maize^12,13^. However, they do not show low-P adaptation. Knowledge of the evolution of the paleo-genome and sub-genomes in white lupin will contribute to our understanding of its adaptations to low P levels.

In this study, we use the long-read sequencing of PacBio technology combined with high-throughput chromatin capture (Hi-C) datasets, as well as mRNA-sequencing (mRNA-seq), comparative and evolutionary genomic analysis, pharmacology assays, genetic transformation, physiology, and biochemistry analyses to characterize the reference genome of white lupin and investigate its chromosomal evolution and the molecular basis of its adaptation to low-P availability.

## Results

### Pseudo-chromosome construction of the white lupin genome

We assembled the genome of the white lupin cultivar Amiga with combined datasets from third-generation long-read SMRT sequencing (PacBio) and long-range, Hi-C sequencing. We confirmed that the white lupin plant used for sequencing had 25 pairs of chromosomes using in situ hybridization (Supplementary Fig. 1). We then generated 60 Gb Illumina Solexa 150 bp paired-end reads data and estimated the genome size of white lupin as 584.51 Mb by 17 K-mer counting. We produced 84.29 Gb (~144×) PacBio reads data (Supplementary Table 1) and assembled the data into contigs using the software Canu^14^, followed by sequence polish^15^ and filtering. We obtained 3171 contigs with a total size of 558.74 Mb. The contig N50 was 1.76 Mb; the largest contig was 9.48 Mb (Table 1). A Hi-C library was constructed and generated ~100-fold coverage of Hi-C linkage data (100 bp paired-end reads). We then linked the contigs into scaffolds based on the Hi-C data^16^ with the help of a previously published linkage map^17^ (Supplementary Fig. 2). Finally, we obtained 1580 scaffolds, and the scaffold N50 was 18.66 Mb (Table 1). The 25 largest scaffolds comprised 1616 contigs, which accounted for 84.87% (474.20 Mb) of the assembled genome and corresponded to the 25 chromosomes of white lupin (Supplementary Table 2 and Supplementary Fig. 3). We compared this assembled genome with a currently released white lupin genome^18^, and found that they have a good chromosomal synteny relationship, except for some small-scale segment inversions (Supplementary Fig. 4).

**Table 1:** The assembly statistics of white lupin genome.

Type	Contig		Scaffold	
	Size (Mb)	Number	Size (Mb)	Number
Maximum	9.48	1	25.25	1
N50	1.76	71	18.66	14
N90	0.052	770	1.67	33
Total length	558.74	3171	558.90	1580
Chromosomes			474.20 (84.87%)	25
Genes	/		/	48,719
TEs			262.71 (43.86%)	/

We combined de novo prediction, homology search, and mRNA-seq assisted prediction to predict genes in the genome of white lupin (see Methods), and we obtained 48,719 genes (Supplementary Fig. 3 and Supplementary Tables 3 and 4). BUSCO (Benchmarking Universal Single-Copy Ortholog) analysis with embryophyta datasets^19^ estimated that 95.20% of the conserved single-copy genes were predicted. TEs are a major component of the white lupin genome. We used the tools RepeatModeler, RepeatMasker, and RepeatProteinMask to predict TEs from the white lupin genome^20^ (see Methods). The combined size of all TE sequences was 262.71 Mb and occupied 43.86% of the assembled genome (Supplementary Table 5 and Supplementary Fig. 3). Among the sequences, long terminal repeats were the major component (37.81%).

### The diploid ancestral genome of paleohexaploid white lupin

**Fig. 1:**
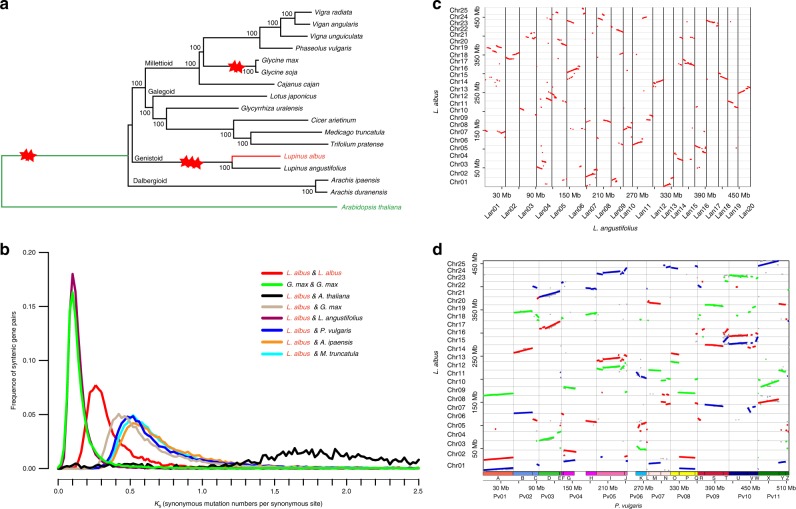
Phylogenetic relationships of white lupin and its genome comparison to the other legume species. **a** Phylogenetic tree of 16 legume species built on synonymous sites from syntenic homologous genes; *A. thaliana* was used as an outgroup species. The two red stars denote the whole-genome duplication event, while the three red stars denote the whole-genome triplication event. **b** Frequency distribution of *K*_s_ values between syntenic genes of compared genomes. **c** Dot plotting of syntenic genes between genomes of *L. albus* and *L. angustifolius*. **d** Dot plotting of syntenic genes between genomes of *L. albus* and *P*. *vulgaris*; the segments from the three sub-genomes in *L. albus* are plotted in red, green, and blue. The source data underlying Fig. 1b are provided as a Source Data file.

*Lupinus albus* and *L. angustifolius* experienced a common whole-genome triplication (WGT) event. We compared the genome of white lupin to 15 other legume species with sequenced genomes (Supplementary Table 6). *Arabidopsis thaliana* was used as the outgroup. First, we determined syntenic gene pairs between pairs of the 16 legume genomes using SynOrths^21^. From these syntenic gene datasets, we obtained 1664 homologous genes that were shared by all 16 genomes. We picked 78,926 synonymous sites from these homologous genes to build a phylogenetic tree (Fig. 1a). The times of divergence between legume species were estimated by calculating *K*_s_ values (the rate of synonymous mutations per synonymous locus) between syntenic genes (Fig. 1b). The results showed that *L. albus* has the closest relationship to *L. angustifolius* (*K*_s_ ~=0.12, divergent ~9.40 million years) (see Methods), followed by a group of combined species from Galegoid and Millettioid that contains *Cicer arietinum* and *Phaseolus vulgaris* (*L. albus* to *P. vulgaris*, *K*_s_ ~=0.51, divergent ~39.97 million years), then by the group of Dalbergioid that contains the two diploid ancestors of cultivated peanut *Arachis ipaensis* and *Arachis duranensis* (*L. albus* to *A. ipaensis*, *K*_s_ ~= 0.52, divergent ~40.75 million years) (Fig. 1). Two relatively recent polyploidization events were found in these analyzed legume species: one is a WGT (*K*_s_ ~=0.28, ~21.94 million years) event shared by *L. albus* and *L. angustifolius* (Fig. 1 and Supplementary Fig. 5), and the other is a whole-genome duplication (WGD, *K*_s_ ~=0.11, ~8.62 million years) event shared by wild and domesticated soybeans *Glycine max* and *Glycine soja*.

The genome of white lupin retained three copies of genomic fragments that show syntenic relationships with the diploid genome of *P. vulgaris*. Based on syntenic gene pairs between *L. albus* and *P. vulgaris*, we extracted 167 fragments longer than 200 kb in white lupin that showed synteny to the genome of *P. vulgaris* (see Methods). These syntenic fragments corresponded to 73.39% and 93.41% of *L. albus* and *P. vulgaris* genomes, respectively (Fig. 1d). These white lupin-specific genomic regions are rich in TE sequences (Supplementary Fig. 3). The repeat sequence-enriched regions may not have been assembled by short reads, or they may not exist in the *P. vulgaris* genome. Based on the syntenic relationships, we found three copies of *L. albus* genomic fragments syntenic to each genomic region in *P. vulgaris* (Fig. 1d).

**Fig. 2:**
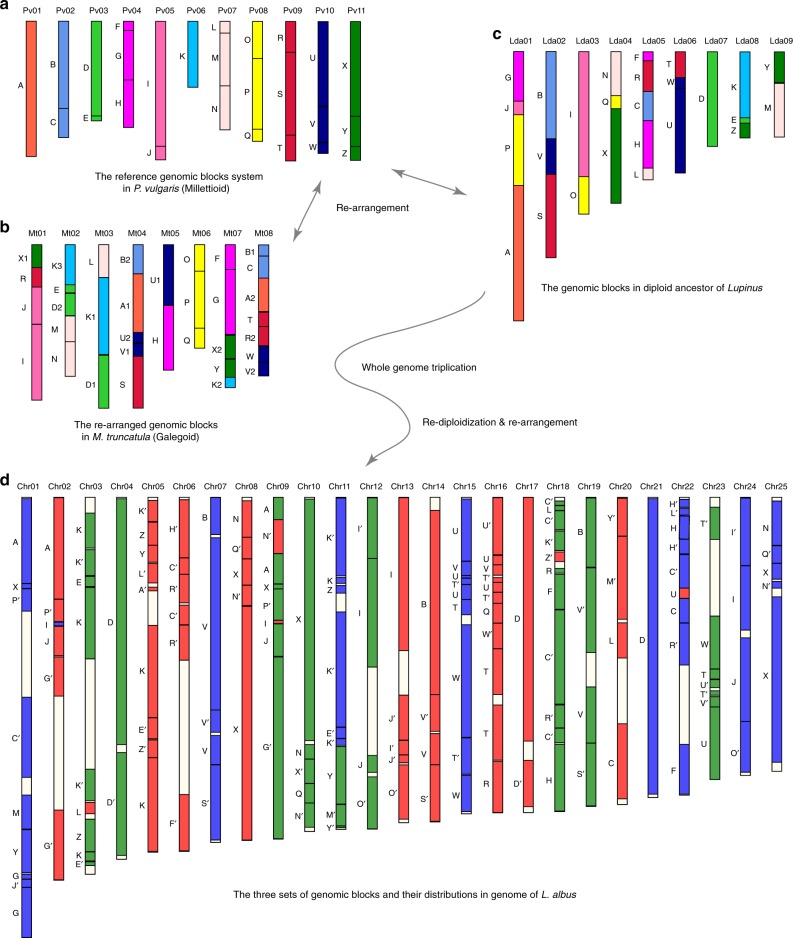
Genome evolution trajectory of white lupin. **a** Distribution of 26 genomic blocks (GBs) in the 11 chromosomes of *P. vulgaris*. **b** The re-arranged GBs in the eight chromosomes of *M. truncatula*. The numbers to the right of the GBs denote the order of fragmented GBs. **c** Distribution of 26 GBs in the deduced nine ancestral chromosomes of the diploid ancestor of white lupin. **d** Distribution of the triplicated 26 GBs in the 25 chromosomes of white lupin; the colors red, green, and blue indicate GBs from sub-genomes LF, MF1, and MF2, respectively. “′” denote inverted GBs.

The diploid ancestral genome of white lupin probably had nine chromosomes. Based on the chromosome syntenic relationships between *Lupinus* and the diploid genome of *P. vulgaris* from Millettioid, we defined a legume-specific GB system to aid comparative genome analysis of *L. albus*. The chromosome regions in *P. vulgaris* (used as a reference) that corresponded to breakpoints appearing in more than one sub-genome in *Lupinus* (both *L. albus* and *L. angustifolius*) were counted and considered as sequence associations that did not exist in the diploid ancestral genome of *Lupinus* (see Methods) (Fig. 1d and Supplementary Fig. 5). A total of 14 such breakpoints were found in the 11 chromosomes of *P. vulgaris*; these breakpoints split the *P. vulgaris* genome into 26 GBs (A–Z) (Fig. 2a and Supplementary Table 7). We further mapped the 26 GBs onto the 25 chromosomes of white lupin (Fig. 2) and screened the white lupin genome to find GB associations that appeared in more than one sub-genome. A total of 18 such GB associations were identified (Supplementary Table 8). These GB associations may have existed in the diploid ancestral genome of *Lupinus*. We concluded that the *Lupinus* diploid ancestor had nine chromosomes and followed the GB orders shown in Fig. 2c. The same results were obtained when we used *M. truncatula* from Galegoid and *Arachis ipaensis* from Dalbergioid as the references to deduce the diploid ancestor of *Lupinus* (Supplementary Figs. 6–8). The genome also shows a karyotype structure similar to previous studies^22,23^. With the diploid ancestor as the reference, we further reconstructed the ancestral status of the three sub-genomes in white lupin (Supplementary Fig. 9). Additionally, we found that two genomic fragments in *P. vulgaris* show variations among legume genomes (Supplementary Table 9). One fragment located in the middle of chromosome Pv04 and between GBs G and H has been lost in the genome of *M. truncatula* (Supplementary Fig. 6), while another located at the head of chromosome Pv06 and before block K has been lost in the genome of *A. ipaensis* (Supplementary Fig. 8), and both fragments have been lost in genomes of *L. albus* and *L. angustifolius* (Fig. 1d and Supplementary Fig. 5).

### Sub-genome dominance and biased TE distribution in *L. albus*

**Fig. 3:**
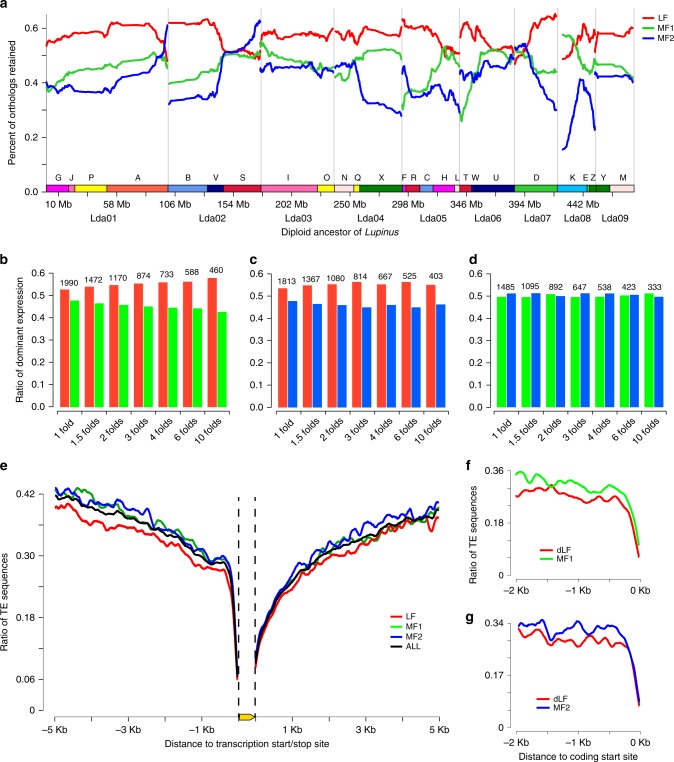
Sub-genome dominance phenomenon observed in the paleo-allopolyploidy genome of white lupin. **a** The density of syntenic genes in three sub-genomes of *L. albus* compared to the deduced diploid ancestor of *Lupinus* (genes of diploid genome *P. vulgaris* were used as the reference). The *x*-axis denotes the physical position of each gene locus in the genome of the diploid ancestor. The *y*-axis denotes the percentage of retained homologous genes in white lupin sub-genomes around each diploid ancestor gene, where 500 genes flanking each side of a certain gene locus were analyzed, giving a total window size of 1001 genes. **b** The number of dominantly expressed paralogs between sub-genomes LF and MF1, with significantly more genes located at sub-genome LF (red) than their paralogs from sub-genome MF1 (green) under different rules to determine the status of dominant expression. **c** Similar to **b**, the number of dominantly expressed paralogs between sub-genomes LF and MF2, with significantly more genes located at sub-genome LF (red) than their paralogs from sub-genome MF2 (blue). **d** The number of dominantly expressed paralogs between sub-genomes MF1 and MF2; no significant differences were found. **e** The difference in average TE density in the vicinity regions of paralogous genes from the three sub-genomes of white lupin. **f** Sub-genomes LF and MF1 with the gene dominantly (**d**) expressed in LF (dLF). **g** Sub-genomes LF and MF2 with the gene dominantly expressed in LF. Source data are provided as a Source Data file.

Sub-genome dominance has been observed in many allopolyploid genomes^10,11,24^, and it also exists in white lupin. We compared gene density among the three sub-genomes of white lupin in units of ancestral chromosomes. One chromosome copy always had significantly more genes (Supplementary Table 10) than the other two copies. One sub-genome had a higher gene density than the other two. This was true except for the head of GB K and the tails of GBs A, S, and H, in which large fragment deletions occurred (Fig. 3a). Copies of nine ancestral chromosomes that had the highest gene density were grouped and named as sub-genome LF (the least fractionated sub-genome), while the copies of nine chromosomes with moderately fractionated genes were grouped as sub-genome MF1 (more fractionated sub-genome 1). The remaining copy was grouped as sub-genome MF2 (more fractionated sub-genome 2). Among the *L. albus* genes that had syntenic orthologs in *P. vulgaris*, 7598, 6227, and 5567 genes were located in sub-genomes LF, MF1, and MF2, respectively. Sub-genome LF retained significantly more genes after WGT than sub-genomes MF1 and MF2 (*P* value = 4.50 × 10^−36^, *χ*^2^ test) (Supplementary Table 10). Dominant expression was observed between paralogous genes from the three sub-genomes of white lupin. Based on the combined information of syntenic genes and sub-genome partitions, we determined 2673, 2418, and 1948 pairwise paralogs between sub-genomes LF and MF1, LF and MF2, and MF1 and MF2, respectively. Using expression data of the white lupin leaf as an example, among these 2673 paralogous genes between LF and MF1, the sub-genome LF (637) had significantly more genes expressed at higher levels than MF1 (533) (two-fold change) (*P* value = 2.59 × 10^−3^, binomial test) (Fig. 3b). Similar results were found between sub-genomes LF and MF2 (*P* value = 2.58 × 10^−3^, binomial test) (Fig. 3c, d and Supplementary Table 11). Significant expression differences were not found for paralogous genes between MF1 and MF2 (Supplementary Table 11). We used different thresholds to determine the dominant expression status as 1-, 1.5-, 3-, 4-, 6-, and 10-fold changes, and similar results were found (Fig. 3b–d).

The density of TE sequences was negatively associated with the dominant expression status of paralogous genes in white lupin. The sub-genome LF had the lowest density of TEs (significant differences in both copy number and sequence density of TEs, Supplementary Table 12) compared to the two other sub-genomes MF1/2 (Fig. 3e). Genes from sub-genome LF that were dominantly expressed had a lower density of TE sequences in their promoter regions (2 kb upstream of the transcription start site) compared to their paralogs (Fig. 3f, g). Sub-genome LF had the highest number of gene copies and most genes were expressed at higher levels as well as at lowest TE density. This suggests that *L. albus* may have experienced a WGT similar to that of allopolyploid species as *Brassica*, in which a “two-step” polyploidization process resulted in the sub-genome dominance phenomenon^25^.

### P-use efficiency genes were largely expanded in white lupin

The expanded genes of *L. albus* differ from those of *L. angustifolius*. We compared genes that had two or more paralogous copies to genes that had only one copy in white lupin. The Gene Ontology (GO) terms GO:0003700 (transcription factor activity), GO:0006412 (translation), GO:0005840 (ribosome), and GO:0005524 (ATP binding) were over-represented in these multiple-copy genes (Supplementary Data 1). Genes annotated with these GO term functions were over-retained after gene fractionation (loss) following WGT, which is consistent with the theory of gene dosage balance^26^. Furthermore, genes with GO terms GO:0006817 (phosphate transport), GO:0016036 (cellular response to phosphate starvation), and GO:0006096 (glycolysis), GO:0006099 (tricarboxylic acid (TCA) cycle), GO:0008610 (lipid biosynthetic process), and GO:0048364 (root development), as well as GO:0048829 (root cap development) were also over-retained in white lupin (Supplementary Data 1), indicating that genes involved in P homeostasis and acquisition are under selection to be retained through gene fractionation. We compared the copy number difference of orthologous genes between *L. albus* and *L. angustifolius*. The two species shared a common WGT event and diverged over ~4 Mya. These two species show differences in the composition of expanded genes. Genes annotated as “glycolysis,” “tricarboxylic acid cycle,” “lipid biosynthetic process,” “ATPase activity,” and different types of phosphate-related functions increased in *L. albus* compared to *L. angustifolius* (Supplementary Data 2). This indicates that over-retaining of genes in these pathways occurred in *L. albus* after it diverged from *L. angustifolius*.

The number of P-use efficiency (PUE) genes has increased through WGT, tandem, and dispersed duplications in white lupin. For genes generated through WGT, we found that GO terms related to the phosphate biology process and root development were enriched in over-retained genes (having more than one copy of paralogs) in both *L. albus* and *L. angustifolius* (Supplementary Data 3 and 4). For the tandem duplicated genes, GO terms such as “phosphate transport,” “ATPase activity,” “lipid transport,” and “root hair cell differentiation” were enriched in *L. albus* (Supplementary Data 5), but not in *L. angustifolius* (Supplementary Data 6). For genes grouped into “dispersed duplication,” many PUE-related GO terms such as “phosphate transport” and “cellular response to phosphate starvation” were enriched in *L. albus*, but not in *L. angustifolius* (Supplementary Data 7 and 8). These results suggest that the high use efficiency of P developed along with genes amplified through WGT in *Lupinus* and further expanded through tandem and dispersed duplications in *L. albus* after its divergence from *L. angustifolius*.

### Adaptive pathways of white lupin to low-P availability

P deficiency induces a low-P adaptive response in white lupin. We performed mRNA-seq analysis on leaves, stems, and roots of white lupin under P-deficient (−P) and P-sufficient (+P) conditions (see Methods). Cluster roots from the −P condition were further dissected into four parts: the pre-emergent zone (PE, 2–3 cm adjacent to the root tips of first-order laterals), young cluster roots (YCRs), mature cluster roots (MCRs), and old cluster roots (OCRs) (Supplementary Fig. 10). We identified a total of 12,380 genes differentially expressed in leaves, stems, and different types of root tissues under P deficiency compared to those under +P conditions (|log_2_ fold change| ≥ 1; FDR (false discovery rate)-adjusted *P* value < 0.01) (Supplementary Fig. 11). Mfuzz clustering and GO enrichment analyses determined that these low-P-induced genes have a strong enrichment in GO terms associated with lipid metabolic process, carbohydrate metabolic process, and transport, as well as cell cycle and motor activity (Supplementary Fig. 12), showing a classical phosphate-starvation response that is consistent with previous studies in *L. albus*^27,28^ and *A*. *thaliana*^29^.

**Fig. 4:**
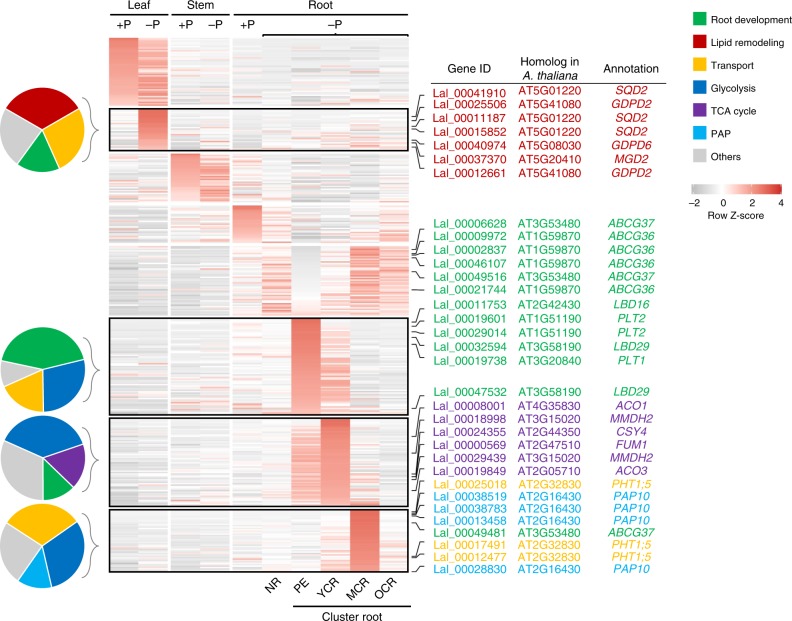
Expression profiles of the differentially expressed P-use efficiency genes of white lupin under P-sufficient or P-deficient conditions. PUE, P-use efficiency; +P, P-sufficient condition; −P, P-deficient condition. Roots from the P-deficient condition were further dissected into normal roots (NRs) and four parts of cluster roots based on the developmental stages, including pre-emergent zone (PE, 2–3 cm behind the root tip of first-order laterals), young cluster roots (YCRs), mature cluster roots (MCRs), and old cluster roots (OCRs). Nine clusters were identified by Mfuzz clustering analysis. Functional categorization of the clusters containing genes with the higher expression in leaves, PE, YCRs, and MCRs under P deficiency is shown on the left. Several key homologs of the *A. thaliana* PUE genes are given on the right. *SQD*, sulfoquinovosyltransferase; *GDPD*, glycerophosphodiester phosphodiesterase; *MGD*, monogalactosyl diacylglycerol synthase; *ABCG36*, ATP-binding cassette G36; *LBD*, lateral organ boundaries domain; *PLT*, plethora; *ACO*, aconitase; *MMDH*, mitochondrial malate dehydrogenase; *CSY*, citrate synthase; *FUM*, fumarase; *PHT*, phosphate transporter; *PAP*, purple acid phosphatase.

There are 512 genes that function in low-P response pathways referred to the previous studies in *A. thaliana*^3,29^ and white lupin^27,28^. Thus, we identified 882 putative orthologs of these 512 genes and termed them as PUE genes (Supplementary Data 9–11). These PUE genes show similar patterns of biased distribution and dominant expression to that of the sub-genome level. More than 40% of these PUE genes in white lupin (363 out of 882) showed two-fold changes of gene expression in the −P treatment (Fig. 4, Supplementary Table 13, and Supplementary Data 12 and 13). More importantly, consistent results were found compared to previous transcriptome studies in *L. albus*^27,28^, including up-regulated genes—under P deficiency—that encode sulfoquinovosyltransferase and monogalactosyl diacylglycerol synthase, which are involved in remodeling membrane lipid composition most probably to remobilize P from phospholipids^30^ as well as mitochondrial malate dehydrogenase, aconitase, and phosphate transporter, which are involved in carbon metabolism and phosphate transport (Fig. 4). The differentiation in transcription levels of these PUE genes was further supported by evidence from physiological datasets (Supplementary Figs. 13–16).

The low-P adaption in white lupin was found to be associated with duplications of the gene *PAP10* (purple acid phosphatase 10). PUE genes involved in the TCA cycle, glycolysis, lipid remodeling, phosphate transport, and auxin-regulated root development were expanded and showed a biased retention of paralogs in *L. albus* compared to *M. truncatula* and *L. angustifolius* (Supplementary Data 11 and 14). In particular, *PAP* genes, which are important for organic P mobilization in cluster roots of white lupin^31^, were largely expanded in the white lupin genome. White lupin has eight copies of *PAP10* compared to only one copy in *A. thaliana*. Among the eight *PAP10* genes, three, three, and two copies have arisen from polyploydization, tandem, and dispersed duplications, respectively (Supplementary Fig. 17 and Supplementary Data 15). Peak transcript abundance of *PAP10* genes coincides with the increase in acid phosphatase (AP) activity that occurs in cluster roots in −P plants (Fig. 4 and Supplementary Fig. 18). To further investigate the roles of *PAPs* in white lupin adaptation to low-P availability, we generated *PAP10* (*Lal_00013458*) and *PAP12* (*Lal_00004477*, ortholog of *A. thaliana PAP12*) (Supplementary Table 14) overexpressing hairy roots in white lupin using the *Agrobacterium rhizogenes*-based transformation system. When phytate (main organic P in soil) was the only P source in 1/2 Murashige and Skoog basal media, *35S:PAP10* and *35S:PAP12* transgenic hairy roots were found to contain more P per gram of dry weight than those transformed with empty vectors (Supplementary Figs. 19 and 20), supporting the function of multi-copy *PAP10* genes in releasing P from external organic P esters.

**Fig. 5:**
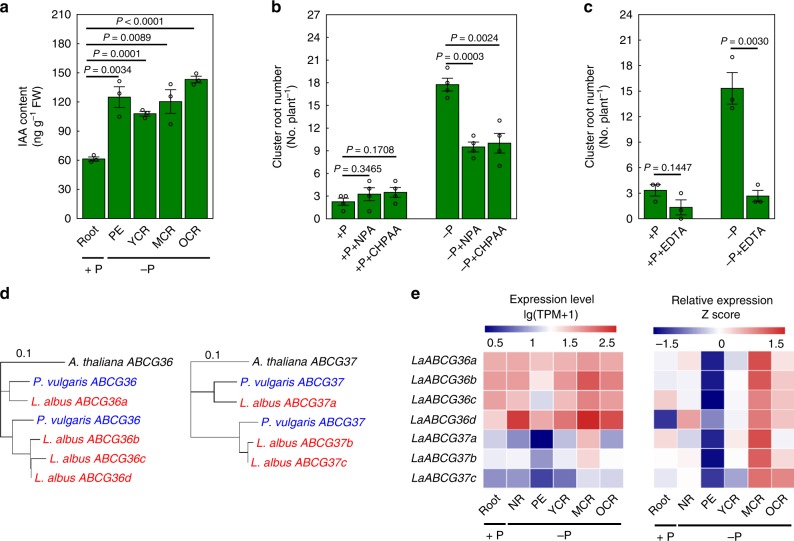
Roles of auxin modulation in cluster root formation of white lupin. **a** Quantification of IAA contents (ng per g of fresh weight, ng g^−1^ FW) in different types of white lupin roots. *n* = 3 plants. Effects of auxin polar transport inhibitors (**b**) and EDTA (**c**) on the formation of cluster roots in white lupin under P-sufficient (+P) or P-deficient (-P) conditions. *n* = 4 plants for (**b**) and *n* = 3 plants for (**c**). Error bars indicate s.e.m. *P* value was calculated using the unpaired two-sided Student’s *t* test. CHPAA, auxin influx inhibitor 3-chloro-4-hydroxyphenylacetic acid; NPA, auxin efflux inhibitor 1-naphtuylphthalamic acid; EDTA, metal chelator ethylenediaminetetraacetic acid. **d** Neighbor-joining phylogenetic tree of *ABCG36* and *ABCG37* genes from *A. thaliana*, *P. vulgaris*, and *L. albus*. **e** Expression profiles of *LaABCG36a–d* and *LaABCG37a–c* in roots under P-sufficient (+P) condition and normal roots (NRs) or different developmental stages of cluster roots under P deficiency (−P). PE, pre-emergent zone, 2–3 cm behind the root tip of first-order laterals; YCR, young cluster root; MCR, mature cluster root; OCR, old cluster root. The source data underlying Fig. 5a–c are provided as a Source Data file.

Besides these 882 PUE genes determined as aforementioned, we further found *LaABCG36*s and *LaABCG37*s as the potential genes functioning in the cluster root formation through auxin modulation in white lupin. Auxin is involved in P deficiency-induced formation of cluster roots in white lupin. Variation of indole-3-acetic acid (IAA) contents in different stages of the cluster roots was observed (Fig. 5a). Furthermore, the auxin influx inhibitor 3-chloro-4-hydroxyphenylacetic acid (CHPAA) and efflux inhibitor 1-naphtuylphthalamic acid (NPA) treatments resulted in a substantial decline in cluster root number under P deficiency conditions (Fig. 5b), which is consistent with a previous report^32^. Ethylenediaminetetraacetic acid (EDTA), a metal chelating agent, can also inhibit the formation of cluster roots under −P hydroponic solution (Fig. 5c). The activity of AAA proteins (ATPases Associated with diverse cellular Activities) can be inhibited by EDTA^33^. Members of the G subgroup of the ATP-binding cassette transporter, *ABCG36*, and its homolog, *ABCG37*, contain two AAA domains. The two genes act redundantly to transport the auxin precursor IBA (indole-3-butyric acid) in *A. thaliana*^34^. This leads to fluctuation of the level of auxin that is required for the development of lateral roots in *A. thaliana*^34^. Here, we determined four orthologous genes (*LaABCG36a*–*d*) of *ABCG36* and three orthologous genes (*LaABCG37a*–*c*) of *ABCG37* in *L. albus* (Supplementary Table 14), but only two and two, respectively, in *P. vulgaris* (Fig. 5d). Four orthologs of *ABCG36* and two orthologs of *ABCG37* were expanded through the WGT event, while one ortholog of *ABCG37* was expanded through tandem duplication in white lupin (Supplementary Fig. 17). RNA-sequencing analysis showed that *LaABCG36s* and *LaABCG37s* had the lowest expression in the PE zone, followed by a continuous expression that increased during the outgrowth and maturation of cluster roots compared with roots under +P conditions (Fig. 5e). In addition, phosphoproteomics analysis showed that the relative phosphorylation levels of *LaABCG36b* and *LaABCG36c* were increased by about 2.6- and 5.4-fold in white lupin plants under P deficiency compared to those under −P condition (Supplementary Data 16). Altogether, these results strongly suggest that auxin modulation in white lupin is important for cluster root formation by involving two potential genes, *LaABCG36s* or *LaABCG37s*.

## Discussion

White lupin is a dinitrogen (N_2_)-fixing legume. It develops cluster roots that remobilize and efficiently use soil P, and thus is a model crop for studying plant adaption to low-P availability in the soil. Through the assembly and analysis of the genome of white lupin (Table 1 and Supplementary Fig. 3), we deduced its diploid ancestor before WGT (Figs. 1 and 2) and reconstructed three sub-genomes as well as determined sub-genome dominance and biased TE distribution in white lupin (Fig. 3). Furthermore, we found that the PUE genes have been largely expanded through WGT, tandem, or dispersed duplications (Supplementary Fig. 17). Taken together, these results increased our knowledge on the genome evolution, as well as provide clues to the low-P adaptation of white lupin from the genome perspective. Additionally, protein phosphorylation might be involved in the regulation of PUE genes (Supplementary Data 16).

Four main biological pathways have likely co-evolved as adaptations of white lupin to low levels of P (Supplementary Fig. 21). The first pathway involves carbon fixation and the supply of carbon to roots^4^. White lupin was observed to largely maintain photosynthetic carbon fixation after 4 weeks of P deficiency (Supplementary Figs. 13 and 14). We found that genes related to sucrose transport were expanded (Supplementary Data 14), such as orthologs (*Lal_00013937* and *Lal_00026769*) of *AtSWEET7*^35^. These expanded genes were significantly up-regulated under P deficiency, and they should play an important role in sucrose transport from shoot to root.

Secondly, we identified genes whose expressions were associated with variation of IAA contents during cluster root formation in white lupin (Fig. 5a). These genes are orthologs of two IBA transporter genes *ABCG36* and *ABCG37*, which have not been reported as PUE genes in response to P starvation in *A. thaliana* or white lupin, but are functioning in auxin-regulated adventitious root formation in *A*. *thaliana*^36^. White lupin has four and three copies of *ABCG36* and *ABCG37*, respectively, compared to only one copy in *A*. *thaliana* (Fig. 5d). These multiple copies of IBA transport genes have mainly arisen from polyploydization duplication (Supplementary Fig. 17). Moreover, the phosphorylation levels of *LaABCG36b* and *LaABCG36c* were up-regulated under P deficiency (Supplementary Data 16). Therefore, the genes *LaABCG36s* and *LaABCG37s* were considered as potentially involved in the cluster root formation in white lupin through the regulation of auxin homeostasis.

Thirdly, bursts of organic acid and AP extrusion in cluster roots were previously observed to be associated with their ability toward external P remobilization from soil^2^. With the genome data, we found that genes involved in glycolysis, TCA cycle, and dark fixation of CO_2_, which are required for the synthesis of organic acids in cluster roots^37,38^, were largely expanded in white lupin (Supplementary Data 14). Meanwhile, the increased expression profiles of genes involved in these processes are consistent with reported transcriptome studies in *L. albus*^27,28^ and are further supported by physiological data (Supplementary Figs. 13–16). These genes include *PEPC* (*PHOSPHOENOLPYRUVATE CARBOXYLASE*) (Supplementary Fig. 17), which plays important roles in dark fixation of CO_2_ and whose protein phosphorylation level was significantly up-regulated under P deficiency (Supplementary Data 13 and 16), as well as *PAP*, one of the most well-studied groups of phosphatase genes linked to Pi remobilization^31^, whose overexpressing in hairy roots can mobilize P efficiently from organic P esters (Supplementary Figs. 19 and 20) in white lupin.

Fourthly, with respect to the reuse of internal P, we found that genes with a higher expression level in leaves under P deficiency (Fig. 4) were involved in the remodeling of membrane lipid composition most probably to remobilize P from phospholipids, which should be important to the internal P reuse in white lupin. Finally, it is likely that the copy number expansion and function evolution (such as expression and phosphorylation regulation) of genes involved in the four main pathways have contributed to white lupin’s adaptation to low P.

In conclusion, P is a limited resource derived from phosphate rock. The ready supply of P will eventually be depleted. Heavy P fertilizer use in farmland can also lead to environmental pollution. The legume white lupin provides an opportunity to study the mechanism of plant adaption to low available soil P. Understanding the high PUE in white lupin may also help to overcome the inhibition of N_2_ fixation under low levels of P in other leguminous crops. The dissection of the genome sequences of white lupin provides insights into its genome evolution and low-P adaptation. The results of this study may contribute to the breeding of crops with high PUE traits.

## Methods

### Plant material and genome survey

White lupin (*L. albus*) cv. Amiga was used for genome sequencing. Seeds were provided by Professor Feng Yan at the Justus Liebig University, Germany. Lupin plants were grown hydroponically in a +P or −P nutrient solution as described by Yan et al.^39^ and were then moved to a greenhouse. If not otherwise specified, 5-week-old plants were used for the measurements in this study.

DNA was isolated from plants using a standard magnetic beads genomic DNA extraction method. Data from 60 Gb Illumina Solexa 150 bp paired-end reads (350 bp insert library) were generated and the genome was estimated using a K-mer size of 17 bp by JellyFish and GenomeScope^40,41^.

### Genome sequencing and assembly

Approximately 84.29 Gb PacBio sequencing data were generated, covering about 140-fold of the estimated genome size of white lupin. The average size of the PacBio reads was ~8.5 kb, and there were more than 80.67 Gb data with reads longer than 3 kb. The genome assembler Canu^14^ was used for PacBio reads correction, trimming, and assembly following the standard pipeline. The following parameters were used in the assembly: minReadLength = 1000, GenomeSize = 0.7 g, minOverlapLength = 500 bp, and corOutCoverage = 200. The assembly graphs were checked by Bandage^42^ to estimate the assembly quality on the heterozygous, homologous, and repetitive regions. Finally, 3171 contigs were obtained (Table 1). These contigs were further polished with ~60× coverage of Illumina Solexa 150 bp paired-end reads to remove sequencing errors using Pilon^15^ and Bowtie2^43^.

The Hi-C library^16^ was constructed using the *Hin*dIII enzyme with fresh leaf tissue of white lupin by the Proximo Hi-C Plant Kit following standard protocols, and 288 M of 150 bp paired-end reads were generated on the Illumina HiSeq2000 platform. These Hi-C reads were mapped to the assembled contigs using Bowtie2^43^ (BOWTIE2_GLOBAL_OPTIONS = –very-sensitive -L 30 –score-min L,−0.6,−0.2 –end-to-end –reorder; BOWTIE2_LOCAL_OPTIONS = –very-sensitive -L 20 –score-min L,−0.6,−0.2 –end-to-end –reorder), and 38.16% valid mapping reads were obtained. These mapped datasets were submitted to Juicer^44^ software and 3ddna^45^ for grouping, ordering, and orienting (scaffolding) of the contigs. The linking results were also manually curated to correct mis-joins and mis-assemblies based on visualization with JuicerBox^46^.

### Repetitive elements’ prediction

The de novo repeat library of white lupin was constructed using the pipeline of RepeatModeler^20^. TE families with lengths longer than 100 bp and N content <5% were selected, and we identified 10,550 candidate TE families as the TE library. TE sequences in the genome of white lupin were further identified using homology-based searches against both Repbase and the de novo TE library. At the DNA level, TEs were identified using RepeatMasker (http://www.repeatmasker.org/), and at the protein level, TEs were identified using RepeatProteinMask^20^. The results were combined, and redundant TE elements that overlapped (≥80%) to other ones were filtered out.

### Protein-coding gene prediction and annotation

After pre-masking of TE sequences, genes were predicted via ab initio prediction, homology-based searches, and mRNA-seq-assisted prediction. Augustus^47^ and GeneScan^48^ were used in ab initio gene prediction. In homology searches, we collected protein sequences of *Arabidopsis thalina*, *P. vulgaris*, *Cajanus cajan*, *L. angustifolius*, *G. max*, *C. arietinum*, *Pyunus persica*, *M. truncatula*, *A. duranensis*, *Vigan angularis*, and *Lotus japonicas*, and used Genewise v2.4.1^49^ software. We used mRNA-seq datasets on tissues of root, leaf, and stem of white lupin to assist gene prediction. Total RNA was isolated using TRIzol reagent (Invitrogen) according to the manufacturer’s instructions. An independent mRNA-seq library was prepared by the Beijing Genomics Institute (Shenzhen, China), and sequencing was performed on the BGISEQ500 platform (Illumina). Hisat2, Stringtie, and TransDecode^50–52^ were used to assemble mRNA-seq data to unigenes and to perform gene prediction. All gene prediction datasets were combined by Maker^53^ to generate the final gene set of white lupin.

The predicted genes of white lupin were further aligned to the Swiss-Prot and TrEMBL databases^54,55^ with BLASTP at an *E* value 1 × 10^−5^, and the most significant hits were retained. InterPro^56^ was used to annotate motifs and domains in predicted genes by comparison with Pfam, PRINTS, PROSITE, ProDom, and SMART^57–60^ databases. Additionally, the white lupin genes were mapped to KEGG (Kyoto Encyclopedia of Genes and Genomes) pathway maps^61^ based on the most probable Swiss-Prot hit for each gene.

### Identification of syntenic genes

The syntenic orthologs of *L. albus* genes in *P. vulgaris* and other legume species were determined by both sequence similarity and the sequence homozygy of their flanking genes using SynOrths^21^ with *L. albus* as the query genome and each of the other legume species as the subject genome. *Arabidopsis thaliana*, a member of the Brassicaceae, is highly divergent from legume species. Genome synteny between *A. thaliana* and legume species was largely absent. Therefore, we determined orthologous genes (only sequence homology, rather than genomic synteny) between *A. thaliana* and legume species.

### *K*_s_ and phylogenetic analysis

Protein sequences from *L. albus* were aligned with the syntenic orthologs from 16 legume genomes (Supplementary Table 6) using MUSCLE^62^. Protein alignments were translated into coding sequence alignments using an in-house Perl script. *K*_s_ values were then calculated based on the coding sequence alignments using the method of Nei and Gojobori as implemented in the KaKs_calculator^63^. The *K*_s_ values of all syntenic orthologs between *L. albus* and legume species were plotted as histograms (Fig. 1b). For the phylogenetic analysis, the MUSCLE alignments performed in the *K*_s_ analysis were used. The genotypes of 78,926 synonymous loci were extracted from multiple alignments on each of these 1664 syntenic orthologs shared by all 16 legume species. The 78,926 synonymous loci were concatenated into a single sequence for each species, and a phylogenetic tree was constructed with the concatenated sequences using the neighbor-joining method and plotted with the MEGA software (Fig. 1a).

### Analysis of WGT

*K*_s_ values were calculated for the homologous gene pairs within *L. albus* and between *L. albus* and the other legume species. The greatest *K*_s_ value for white lupin peanut (*K*_s_ ~=0.52) revealed the divergence time of these two species, while the peak for white lupin soybean (*K*_s_ ~=0.51) separated the *Lupinus* lineage-specific WGT event (*K*_s_ ~=0.28) from α WGD (*K*_s_ ~=0.11) shared by *G. max* and *G. soja* (Fig. 1a). The neutral mutation rate *ρ* or the constant number 6.38 × 10^−9^ was applied to calculate the time of divergence^6^.

### Determination of syntenic fragments

Continuously distributed syntenic gene pairs along chromosomes in two genomes were identified as intact ancestral fragments that were inherited by the two species. Factors such as local structural variations and assembly incompetence may exist in either or both genomes. Therefore, local syntenic gene pairs may not be distributed immediately adjacent to others, although they are located in a synteny fragment. We thus determined that if genes from two pairs of syntenic genes were interrupted by <50 genes (inserted in between) or had a distance <200 kb in both genomes, then the two gene pairs were grouped in one syntenic fragment. We performed the analysis between *L. albus* and *P. vulgaris*, as well as between *L. angustifolius* and *P. vulgaris* using the same method (Fig. 1c, d).

### Definition of GBs in the Fabaceae

We selected three genomes (*L. albus*, *L. angustifolius*, and *P. vulgaris*) for detailed genomic structure comparisons. The diploid genome of *P. vulgaris* was used as the reference to determine its arrangement of genomic fragments to *Lupinus*, since it shows reasonable divergence from *Lupinus* while possessing good genomic synteny to *Lupinus* genomes (Fig. 2). Syntenic gene pairs were used as painting primers in comparative chromosome painting^64^ analysis to determine the relationships of shared genomic fragments between species. We then scanned across the 11 chromosomes of *P. vulgaris* to find the breakpoints occurring simultaneous to the paralogous fragments in the three sub-genomes of white lupin. Finally, a framework of 26 GBs was built for the comparative genomic analysis in *Lupinus* and other legume species.

### Deciphering the ancestral diploid genome of *Lupinus*

The GB information was mapped from *P. vulgaris* to the three sub-genomes of white lupin (Supplementary Table 7). We then searched the GB associations in the genome of *L. albus* and compared these with the GB associations in *P. vulgaris*. For the GB associations not existing in *P. vulgaris* but appearing in more than one sub-genome of *L. albus*, we counted and recorded these as GB associations that are specific to the diploid ancestor of *L. albus* (Supplementary Table 8). The same analysis was also performed between *L. angustifolius* and *P. vulgaris*, and similar results were obtained. With these GB associations and their orders in the genome of white lupin, we deduced that the diploid ancestor genome of white lupin had nine chromosomes (Fig. 2c).

### Reconstruction of three sub-genomes in white lupin

The white lupin genome is composed of three sub-genomes, each containing 26 GBs. We annotated these GBs along the 25 chromosomes of white lupin. Using the deduced nine chromosomes of the diploid ancestor as the reference, the triplicated copies of each diploid chromosome in the hexapolyploid ancestor (before rearrangement) of white lupin were reconstructed (Supplementary Fig. 9). Two rules were followed in the linking of genomic fragments: (1) individual chromosome copies could not contain overlapping and redundant genomic fragments, and (2) the final reconstructed optimum sub-genomes were the ones involving the fewest genomic rearrangements compared to the other possibilities.

### Comparison of dominant expression between gene doublets

The mRNA-seq datasets from three tissues (root, stem, and leaf) were used in this analysis. Reads were mapped to genes of white lupin using Hisat2^51^, and FeatureCount was used to extract mapped reads for each gene to compute the TPM values as the expression levels of the genes^65^. Paralogous gene pairs (gene doublets) were extracted from the homologous genes among the three sub-genomes of white lupin. To avoid the effects of false mapped reads, all genes were sorted from high to low based on their expression levels. The bottom 1% of genes were considered as not expressed. When comparing the dominant expression status in each gene doublet, at least one of the two genes had an expression value >5.

### TE distribution in neighboring regions of white lupin genes

We used a 100 bp sliding window with a 10 bp step moving across the 5′ and 3′ flanking regions of genes to estimate the TE density around each gene of white lupin. In each 100 bp window, we calculated the ratio of TE nucleotides and then averaged the ratio across subsets of the white lupin genes. The averaged values were plotted as the TE density in the flanking region of these subsets of white lupin genes (Fig. 3e–g).

### Gene retention after WGT in white lupin

We counted the number of syntenic genes in *L. albus* in relation to each *P. vulgaris* gene. There were 7598, 6227, and 5567 *P. vulgaris* genes that had one, two, and three paralogs in the three sub-genomes of white lupin, respectively. Using the GO for each *P. vulgaris* gene, we then investigated the gene retention features in *L. albus*. For each GO term, we compared its enrichment in genes with two or more copies to genes with only one copy in white lupin. The one-sided Fisher’s exact tests tests were performed to check the significance of GO enrichment. Similar analyses were performed between different gene groups (Supplementary Data 1–8).

### Genes expanded through different ways of duplications

Three main types of duplicated genes were separated in white lupin. Genes having syntenic relationships with any of the 16 legume species (Supplementary Table 6) were considered as either being inherited or expanded through polyploidization (WGT) in white lupin. We then determined neighboring genes with sequence homology to each other in the genome of white lupin as being expanded through tandem duplication. Genes that were not classified into WGT or tandem duplication were grouped as transposition or other ways of duplication and were termed as dispersed duplication genes.

### Response of white lupin to P deficiency

Leaves, stems, and roots of white lupin under −P and +P hydroponic solution were harvested after 28 days of culture. Roots from the −P condition were further dissected into normal roots and four parts of cluster roots based on the developmental stages, the PE (2–3 cm behind the root tip of first-order laterals), YCRs, MCRs, and OCRs (Supplementary Fig. 10). Three biological replications were used for each experiment. The processes of mRNA extraction, sequencing, and gene expression level calculation followed the BGI standard methods. Differential expression analysis between −P and +P conditions was performed using DESeq package^66^ (v.1.10.1) in R (v.3.6.1). The resulting *P* values (negative binomial distribution) were adjusted using Benjamini and Hochberg’s approach for controlling the FDR. Genes with |log_2_ fold change)| >1 and FDR value < 0.01 were defined as differentially expressed. The expression dynamics of PUE genes in white lupin were visualized using the “ComplexHeatmap” R package (v.2.2.0).

### Identification of PUE genes in white lupin

To study the high PUE genes in white lupin, genes involved in PUE-related pathways (e.g., TCA cycle, glycolysis, CO_2_ fixation, and root development) from *A. thaliana* (Supplementary Data 9) were used as subject sequences. All protein sequences of *M. truncatula* and *L. angustifolius* and white lupin were aligned to these PUE proteins by Blastp with >1 × 10^−20^ *E* value, >50% coverage, and >35% identity in sequence alignment. The syntenic relationship of these genes in white lupin to *M. truncatula* or *P. vulgaris* were checked for confirming the WGT events.

### Phosphoproteomic analysis

Proteins from roots were extracted by TCA/acetone mixture. After protein quantification, phosphopeptides were enriched by TiO_2_ beads. The enriched phosphopeptides were then separated by UltiMate 3000 HPLC (high-performance liquid chromatography) (Thermo Fisher Scientific, San Jose, CA, USA). After a precursor scan of intact peptides was measured in the LTQ Orbitrap Velos (Thermo Fisher Scientific) by scanning from *m/z* 350−1500 (with a resolution of 120,000), the eight most intense multiple-charged precursor ions were subjected to collision induced for 30 ms with a normalized collision energy of 35.0. Automatic gain control targets were 10,000 for tandem mass spectrometry (MS/MS) scans and 100,000 ions for Orbitrap scans. Data were analyzed using Progenesis LC-MS software (v4.1, Nonlinear Dynamics), and label-free quantification was performed using a liquid chromatography-MS/MS system^67^.

### IAA analysis

Root homogenate was prepared with cold buffer (methanol:H_2_O:acetic acid = 80:20:1, v/v/v). After purification by petroleum ether and ethyl acetate, IAA was quantified by HPLC (Rigol L3000, RIGOL Technologies, Inc. China) with a reverse-phase C18 Gemini HPLC column (250 mm × 4.6 mm, 5 μm). The mobile phase consisted of an equal volume mixture of methanol and acetic acid at a flow rate of 0.8 mL min^−1^. Excitation and emission wavelengths were set at 275 and 345 nm, respectively. The amount of IAA in the sample was calculated from the peak area (Fig. 5a).

### Addition of cluster root formation inhibitors

To evaluate effects of EDTA on cluster root formation, lupin plants grown in +P or −P nutrient solution were treated by 10 mM EDTA for 7 days, while control plants were hydroponically grown without EDTA. In terms of auxin influx and efflux inhibitor (CHPAA and NPA) treatment, 30 µM of CHPAA or NPA was added to the nutrient solution, and the number of cluster roots was recorded after 5 days of inhibitor addition. To evaluate the effects of H^+^ flux on cluster root formation, 30 µM Na_3_VO_4_ was applied to plants for the duration of a 5-day treatment (Fig. 5c and Supplementary Fig. 16c).

### DNA probe preparation for FISH

A synthetic telomere oligo probe labeled with TexasRed-5-dUTP (provided by James A. Birchler, Univ. of Missouri, Columbia, MO, USA) was used for fluorescence in situ hybridization (FISH). The 170 bp repetitive sequence PCR amplified from lupin genomic DNA was labeled with Alexa Fluor 488-5-dUTP (Invitrogen Life Technologies) by the nick translation method^68^.

### Somatic chromosome preparation and FISH

Metaphase chromosome isolation and chromosome spreading were performed according to Kato et al.^68^. After preparation of slides with fluorescence-labeled probes and DAPI (4′,6-diamidino-2-phenylindole; Vector Laboratories, Burlingame, CA, USA), FISH images were captured by a Leica DFC340 FX Digital charge-coupled device camera under a fluorescence microscope (Leica Microsystems, Wetzlar, Germany). FISH images obtained from different channels were merged by Lecia CW4000 FISH software and analyzed with Lecia CW4000 Karyo software (Supplementary Fig. 1).

### Photosynthetic parameters monitoring

After 4 weeks of P deficiency, the fifth fully expended leaf counting from the top of the plant was chosen for the measurements of net photosynthetic rate, stomatal conductance, and intercellular CO_2_ concentration. Photosynthetic parameters were monitored by a portable photosynthesis system (LI-COR 6400-XT, LI-COR, Nebraska, USA) with a 2 × 3 cm^2^ cuvette. The cuvette conditions were as follows: light intensity, 1000 μmol m^−2^ s^−1^; flow rate, 500 μmol s^−1^; sample CO_2_, 400 μmol mol^−1^; and leaf temperature, 29 ± 2 °C (Supplementary Fig. 13a–c). Pigments in leaf tissue were analyzed by spectrophotometry (Supplementary Fig. 14).

### Root exudate collection and analysis

To collect root exudates, +P roots were incubated in a matrix solution containing 0.5 mM K_2_SO_4_, 0.5 mM Na_2_SO_4_, and 0.5 mM CaSO_4_ (pH 6) for 1.5 h, while normal and cluster roots in the −P condition were separately collected. Organic acids in the exudates were determined by HPLC (Agilent 1200, Agilent Technologies, Germany) with an Agilent C18 AQ column (250 mm × 4.6 mm, 5 μm) and a flow rate of 0.5 mL min^−1^. The amount of organic acid was calculated from the peak area (Supplementary Fig. 15).

### Western blotting

Total membrane proteins (15 µg) from roots were separated on 10% (w/v) sodium dodecyl sulfate (SDS)–polyacrylamide gel electrophoresis followed by electrotransfer onto a polyvinylidene difluoride membrane (0.45 µm, Merck Millipore Ltd, USA) with a semi-dry blotting system. H^+^-ATPase was detected using an anti-PMA2 polyclonal antibody (1:2000, v/v). Immunodetection was performed using a monoclonal anti-actin (plant) secondary antibody (Sigma). Intensities of H^+^-ATPase immunoreactive bands were quantified by Gel-Pro Analyzer software 4.0 (Media Cybernetics Inc., Bethesda, MD, USA) and expressed in terms of absolute integrated optical density (Supplementary Fig. 16a).

### Plasma membrane isolation and H^+^-ATPase activity assay

Plasma membrane was isolated from three types of lupin roots: +P roots, −P normal roots, and −P cluster roots. The plasma membrane was isolated by using the method of aqueous two-phase partitioning^39^. Plasma membrane H^+^-ATPase activity was determined in a 0.5 mL reaction mixture containing 15 µg membrane, 50 mM BTP, 15 mM MgSO_4_, 7 mM KCl, and 0.25 mM Brij58 with or without 7 mM vanadate. The reaction was initiated by adding 10 mM ATP and terminated by 2.5 mL stop solution containing 2% (v/v) concentrated H_2_SO_4_, 5% (w/v) SDS, and 0.7% (w/v) (NH_4_)_2_MoO_4_. The absorbance at 700 nm was recorded after 30 min of incubation at room temperature. ATPase activity was calculated as P liberated in excess of vanadate addition control (Supplementary Fig. 16b). The protein concentration in membrane preparations was determined by the Bradford reagent.

### Detection of H^+^ flux by scanning ion-selective electrode technique

The scanning ion-selective electrode technique was used to detect root H^+^ flux. Cluster roots and 2–3 cm apical segments of normal roots were prepared immediately before detection. Roots were washed and immediately immersed and immobilized in the solution (0.1 mM KCl, 0.1 mM CaCl_2_, 0.1 mM MgCl_2_, 0.5 mM NaCl, 0.3 mM MES, 0.2 mM Na_2_SO_4_, pH 6.0) and allowed to equilibrate for 30 min. Then, the roots were moved to the chamber for analysis. H^+^ flux was measured by moving an ion-selective microelectrode along the root at intervals of 30 μm, and the measured point was finally positioned to the elongation zone of the root (Supplementary Fig. 16b, c).

### AP activity assay

AP activity was measured according to the manufacturer’s instructions (Nanjing Jiancheng Bioengineering Institute, China). One unit of AP activity was defined as 1 mg phenol liberation per min at 37 °C. Protein concentration was determined by the Bradford reagent (Supplementary Figs. 18 and 19).

### Construction for transformation vector

The vector pFGC5941, consisting of the CAMV 35S promoter and kanamycin resistance gene, was used. The amplicon of the desired gene (*PAP10* and *PAP12*) from lupin complementary DNA (cDNA) was digested with two restriction enzymes *Bam*HI and *Sma*I, and then the gene was introduced into the T-DNA region of pFGC5941. The constructs were transformed into *Agrobacterium rhizogenes* strain K599 (Shanghai Weidi Biotechnology, China) using the heat-shock method according to the manufacturer’s instructions.

### White lupin transformation and gene function analysis

Lupin seeds were surface sterilized by chlorine gas. The seeds were germinated on half strength Murashige and Skoog medium (PhytoTech Labs, USA) at 25 °C in darkness. After 2 days of germination, the hypocotyl and half of the cotyledon were removed, and the remaining cotyledon was soaked in *A. rhizogenes* solution for 20 min. The cotyledons were then placed on sterile filter paper and incubated at 22 °C in darkness for 3 days. After washing with a 300 mg L^−1^ Timentin solution (Solarbio Life Sciences, China), cotyledons were transferred onto half strength MS solid medium containing 300 mg L^−1^ Timentin (Plant Cell Technology, USA) for hairy root development. The *Bar* gene was PCR amplified to identify the transgenic roots, and the primers are shown in Supplementary Table 15. After 30 days of root development in darkness, hairy roots were transferred into P-omitted MS solution with or without 0.25 mM phytate (myo-inositolhexakisphosphate, sodium salt hydrate; Solarbio Life Sciences, China), and the roots were then sampled after 3 days of treatment.

### Isolation of RNA and quantitative real-time PCR

Total RNA was extracted by TRIzol reagent (Invitrogen). The first-strand cDNAs were synthesized by a PrimeScript^TM^ RT Reagent Kit (TaKaRa, DALIAN). Gene expression level was analyzed on a LightCycler Real-Time PCR Detection System (Bio-Rad) by using SYBR^®^*Premix Ex Taq*^TM^ (TaKaRa, DALIAN). The primers used for quantitative real-time PCR (qRT-PCR) are shown in Supplementary Table 15. All quantifications were normalized to *ACTIN*. The qRT-PCR reactions were performed in triplicate for each of three independent samples. The relative expression of genes was calculated by the 2^−∆∆Ct^ method^69^ (Supplementary Fig. 19a, b).

### Determination of P concentration

For the determination of P concentration, plant tissues were dried and then grinded, and the resulting sample was digested in concentrated H_2_SO_4_. P concentration was spectrophotometrically determined by the ammonium molybdate/vanadate method (Supplementary Figs. 10 and 20).

### Reporting summary

Further information on research design is available in the Nature Research Reporting Summary linked to this article.

## Supplementary information

Supplementary Information

Peer Review File

Reporting Summary

Description of Additional Supplementary Files

Supplementary Data 1–16

## Data Availability

Data supporting the findings of this work are available within the paper and its Supplementary Information files. A reporting summary for this article is available as a Supplementary Information file. The datasets generated and analyzed in the current study are available from the corresponding author on reasonable request. All the genome and annotation datasets of white lupin were deposited to the NCBI BioProject database under accession PRJNA592024. The assembled genome sequences of white lupin have been deposited at DDBJ/ENA/GenBank under the accession JAAEJY000000000. The version described in this paper is version JAAEJY010000000. The white lupin genome datasets are also freely available through http://brassicadb.org/brad/pub/genomes/Lalbus/. The two public mRNA-seq datasets in white lupin under P deficiency were downloaded from the NCBI database under accession SRA145661 and GSE31132. Genome datasets of *A. thaliana* were downloaded from the TAIR database (TAIR10; http://www.arabidopsis.org/index.jsp). Genome sequences of other Legume species were downloaded from the legume information system (https://legumeinfo.org/) as well as the Phytozome database (https://phytozome.jgi.doe.gov/). The source data underlying Figs. 1b, 3, and 5a–c, as well as Supplementary Figs. 10, 13–16, and 18–20 are provided as a Source Data file.

## Source data

Source Data
