# Supplementary material for: The genome evolution and low-phosphorus adaptation in white lupin
Source: Nat Commun. 2020 Feb 26;11:1069. doi: 10.1038/s41467-020-14891-z (PMC7044338; doi:10.1038/s41467-020-14891-z)
Supplement: Supplementary file 1 — Supplementary Information [file 41467_2020_14891_MOESM1_ESM.pdf]

The genome evolution and low-phosphorus adaptation in white lupin

Xu *et al*.

Supplementary Table 1. The statistics of PacBio reads.

Reads length (bp)	Mean length (bp)	Data (Gb)
>3,000	9,730	80.67
>2,000	9,073	82.68
>1,000	8,456	83.94

Supplementary Table 2. The length and the number of contigs in each chromosome of white lupin.

Chromosome ID	Length (bp)	#Contigs
Chr01	25,248,489	62
Chr02	21,956,312	54
Chr03	21,600,612	95
Chr04	20,734,107	114
Chr05	20,351,510	54
Chr06	20,295,173	78
Chr07	19,763,691	111
Chr08	20,164,945	116
Chr09	19,588,981	69
Chr10	19,132,984	128
Chr11	19,043,260	49
Chr12	19,026,298	55
Chr13	18,661,206	41
Chr14	19,024,428	52
Chr15	18,133,549	84
Chr16	17,942,030	18
Chr17	18,080,893	41
Chr18	18,022,918	47
Chr19	17,734,605	25
Chr20	17,727,255	54
Chr21	17,226,637	34
Chr22	17,071,188	66
Chr23	16,183,900	41
Chr24	15,951,480	40
Chr25	15,691,216	88
Total	474,198,567	1,616

Supplementary Table 3. The statistics of genes in each prediction process.

	Gene set	Number	Average mRNA length(bp)	Average CDS length(bp)	Average exon per gene	Average exon length(bp)	Average intron length(bp)
**De novo**	Augustus	107,853	2158.49	1242.58	3.26	380.59	404.40
	SNAP	154,075	2045.67	820.91	3.37	243.90	517.69
**Homolog**	*Arabidopsis thaliana*	105,847	2234.30	931.36	3.54	263.39	513.76
	Phaseolus vulgaris	147,794	2243.47	929.52	3.46	268.72	534.33
	Lupinus angustifolius	207,847	2217.35	917.59	3.52	260.62	515.62
	Glycine max	318,552	2199.43	931.44	3.55	262.67	498.03
	Cicer arietinum	143,849	2049.34	864.75	3.19	270.80	540.08
	Prunus persica	113,716	2225.52	939.59	3.38	278.34	541.27
	Cajanus cajan	355,727	1185.75	568.87	2.11	270.13	557.80
	Medicago truncatula	290,122	1543.77	708.58	2.43	291.55	583.87
	Arachis_duranensis	154,397	1911.86	809.98	3.14	258.04	515.15
	Vigna_angularis	121,300	1960.59	816.93	3.18	256.72	524.10
	Lotus_japonicus	275,330	1566.38	701.61	2.68	261.98	515.32
**RNA-Seq**	-	54,328	4773.11	2348.29	5.95	394.87	490.16
**Maker**	-	48,719	3286.02	1205.57	4.61	261.29	428.40

Supplementary Table 4. The function annotation of predicted genes of white lupin.

Types		Number	%Annotated
**Total genes**		50,253	-
	**InterPro**	40,490	83.11
	**GO**	24,756	50.81
	**KEGG**	35,013	71.87
**Annotated**	**Swissprot**	33,306	68.36
	**TrEMBL**	47,248	96.98
	**KOG**	32,747	67.22
	**Nr**	47,481	97.46
**Overall**		47,842	98.20

Supplementary Table 5. The statistics of different groups of transposable elements in genome of white lupin.

	RepBase TEs		TE Proteins		De novo		RepeatExplorer+Dante		Combined TEs	
	Length (bp)	%in Genome	Length (bp)	% in Genome	Length (bp)	% in Genome	Length(bp)	% in Genome	Length (bp)	% in Genome
**DNA**	6,910,043	1.15359	823,880	0.13754	17,341,454	2.89505	1,860,262	0.31056	201,010,211	3.77506
**LINE**	3,238,272	0.54061	365,799	0.06107	5,767,902	0.96292	952,630	0.15904	8,089,994	1.30718
**SINE**	564,739	0.09428	0	0	283,191	0.04728	0	0	742,035	0.12388
**LTR**	61,751,654	10.30905	53,852,270	8.9903	219,015,780	36.56332	32,368,626	5.40374	227,051,838	37.81179
**Other**	5,760	0.00096	1,599	0.00027	240,766	0.04019	0	0	246,360	0.04113
**Unknown**	0	0	165	0.00003	7,286,866	1.2165	0	0	7,287,031	1.21652
**Total**	70,930,410	11.84139	55,024,110	9.18593	241,267,035	40.27802	35,181,518	5.87333	262,705,502	43.85704

Supplementary Table 6. The number of chromosomes and whole genome polyploidizations of the 16 legume species and an outgroup *Arabidopsis*.

Indes	Species	#Chromosomes	WGP*
1	*Arabidopsis thaliana*	5	1
2	*Arachis duranensis*	10	1
3	*Arachis ipaensis*	10	1
4	*Lupinus angustifolius*	20	3
5	*Lupinus albus*	25	3
6	*Trifolium pratense*	7	1
7	*Medicago truncatula*	8	1
8	*Cicer arietinum*	8	1
9	*Glycyrrhiza uralensis*	-	1
10	*Lotus japonicus*	6	1
11	*Cajanus cajan*	12	1
12	*Glycine soja*	20	2
13	*Glycine max*	20	2
14	*Phaseolus vulgaris*	11	1
15	*Vigna unguiculata*	11	1
16	*Vigna angularis*	11	1
17	*Vigra radiata*	11	1

**: whole genome polyploidizations*

Supplementary Table 7. The 26 genomic blocks and their intervals in genome of *P. vulgaris*.

Index	Genomic Block	Chromosome	Start	Stop
1	A	NC_023749.1	1	50367376
2	B	NC_023750.1	1	32437796
3	C	NC_023750.1	32438390	43275151
4	D	NC_023751.1	1	35225664
5	E	NC_023751.1	35442757	37469608
6	F	NC_023752.1	1	3376840
7	G	NC_023752.1	3914630	22355204
8	H	NC_023752.1	41999933	59662532
9	I	NC_023753.1	1	46489492
10	J	NC_023753.1	46703048	51758522
11	K	NC_023754.1	7470581	31977256
12	L	NC_023755.1	1	4347981
13	M	NC_023755.1	4369254	24354813
14	N	NC_023755.1	24449388	40819286
15	O	NC_023756.1	1	13945638
16	P	NC_023756.1	14763895	41109847
17	Q	NC_023756.1	41146788	45960019
18	R	NC_023757.1	1	11433588
19	S	NC_023757.1	11670041	42660996
20	T	NC_023757.1	42668758	52284309
21	U	NC_023758.1	1	31498796
22	V	NC_023758.1	31784043	45023149
23	W	NC_023758.1	45042905	49040938
24	X	NC_023759.1	1	35229069
25	Y	NC_023759.1	35247003	46675662
26	Z	NC_023759.1	46698852	52205531

Supplementary Table 8. The observed ancestral GB associations in the genome of white lupin.

Index	Chromosome	***P. vulgaris****		***M. truncatula****	
		**GB asso****	**Copies**	**GB asso****	**Copies**
1	Lda01	G/J	3	G/J	3
2	Lda01	J/P	3	J/P[f]	3
3	Lda01	P/A	3	J/P[f]/A	3
4	Lda02	B/V	3	B/V	3
5	Lda02	V/S	3	V/S	3
6	Lda03	I/O	3	I/O	3
7	Lda04	N/Q	3	N/Q	3
8	Lda04	Q/X	3	Q/X	3
9	Lda05	F/R	3	F/R	3
10	Lda05	R/C	3	R/C	3
11	Lda05	C/H	3	C/H	3
12	Lda05	H/L	2	C/L[***]	2
13	Lda06	T/W	3	T/W	3
14	Lda06	W/U	3	W/U	3
15	Lda07	D	3	D	3
16	Lda08	K/E	3	K/E	3
17	Lda08	E/Z	3	E/Z	3
18	Lda09	Y/M	3	Y/M	3

* reference genome used.
** genomic block associations.
[***] sequence fractionated.

Supplementary Table 9. Two genomic fragments show variations among legume genomes.

Location	Dalbergioid<br>*A. ipaensis*	Genistoid<br>*Lupinus*	Galegoid<br>*M. trancatula*	Millettioid<br>*P. vulgaris*
G/fragment/H	exist	lost	lost	exist
fragment/K	lost	lost	exist	exist

Supplementary Table 10. The difference in retained genes among the three copies of ancestral chromosomes in white lupin.

Chromosome	#Reference Genes*	#Synteny genes			*P*-value (χ2 test)**
		LF	MF1	MF2	
Lda01	1,653	903	739	715	1.44×10^{-04}
Lda02	1,697	921	779	774	3.76×10^{-03}
Lda03	1,692	954	802	743	8.36×10^{-05}
Lda04	1,409	801	638	544	3.40×10^{-08}
Lda05	1,781	979	754	624	6.16×10^{-13}
Lda06	1,785	984	761	775	4.49×10^{-06}
Lda07	1,361	761	628	582	1.64×10^{-04}
Lda08	1,680	901	809	523	4.14×10^{-17}
Lda09	696	394	317	287	2.28×10^{-03}
Total	13,754	7,598	6,227	5,567	4.50×10^{-36}

* Genes in diploid species P. vulgaris were used as the reference.

** *P*-value was calculated using the two-sided χ^2 test.

Supplementary Table 11. The difference in the number of dominantly expressed genes between paralougs from each two of the three sub-genomes in white lupin.

Pairs		#Dominant expression (2-folds)			*P*-value (Binomial test)*
		LF	MF1	MF2	
**LF-MF1**	2,370	637	533	/	2.59×10^{-03}
**LF-MF2**	2,170	590	/	490	2.58×10^{-03}
**MF1-MF2**	1,764	/	450	442	0.81

* *P*-value was calculated using the two-sided Binomial test.

Supplementary Table 12. The difference in the density and copy of TEs among the three sub-genomes in white lupin.

Group		LF	MF1	MF2	***P*-value (χ^2 test)***
**Size (bp)**	**Genome**	115,175,050	99,811,333	87,958,197	0.00
	**TE**	39,585,172	37,243,384	31,458,380	
	**TE%**	0.34	0.37	0.36	
**Copy**	**Gene**	8,366	7,015	6,037	9.42E-15
	**TE**	125,592	114,181	103,954	
	**TE/Gene**	15.01	16.28	17.22	

* *P*-value was calculated using the two-sided χ^2 test.

Supplementary Table 13. The number of genes that show two-folds expression changes out of 882 PUE genes on the comparison of P-deficient to P-sufficient plants.

	Two-fold changes		
	**Leaf**	**Stem**	**Root***
**Leaf**	12	1	53
**Stem**	-	2	5
**Root**	-	-	279
**ALL**		11	

*: at least one comparison of root samples from P deficient and sufficient condition.

Supplementary Table 14. Gene symbol used in this study.

Gene ID	Gene symbol
Lal_00021744	*LaABCG36a*
Lal_00002837	*LaABCG36b*
Lal_00009972	*LaABCG36c*
Lal_00046107	*LaABCG36d*
Lal_00049481	*LaABCG37a*
Lal_00006628	*LaABCG37b*
Lal_00049516	*LaABCG37c*
Lal_00013458	*PAP10*
Lal_00004477	*PAP12*

Supplementary Table 15. List of primers for qRT-PCR and identification of transgenic plants.

Gene	Description	Orientation	Sequence (5'-3')
LaACTIN2	constitutively expressed in vegetative tissues, which was selected as housekeeping gene	Forward	AGATTTGGCATCACACTTTCTAC
		Reverse	ATTTGGGTCATCTTCTCTCTGTT
PAP10	secretory purple acid phosphatase 10	Forward	TCGTTCTCATGCATGCACCT
		Reverse	AGACGCGTTCAGATCGTTCA
PAP12	secretory purple acid phosphatase 12	Forward	CAGCATACCGTGAAGCCAGT
		Reverse	GCCTCCACTGCGTATCCATC
bar	*bar* PCR amplified product with a size of 524 bp to selecte transgenic lines	Forward	CTAGATTGTTGAGCAGATCT
		Reverse	ATGAGCCCAGAACGACGCCC

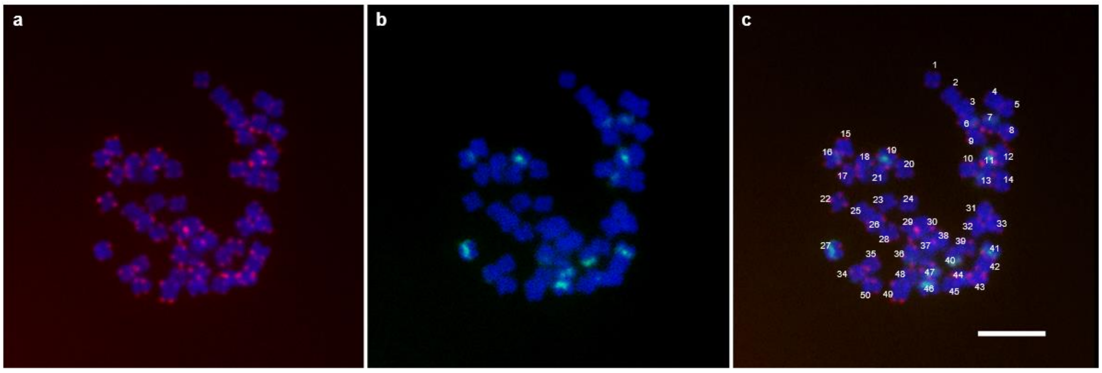

Supplementary Figure 1. Visualization of metaphase chromosomes of white lupin after fluorescence in situ hybridization. (**a**) Texas Red-dUTP–labelled telomere of chromosomes (red). (**b**) Alexa Fluo 488-dUTP labelled a 170 bp repetitive sequence (green) of chromosomes. (**c**) Merged image of a and b. Similar results were obtained for a-c in three independent experiments. Scale bar = 5 µm.

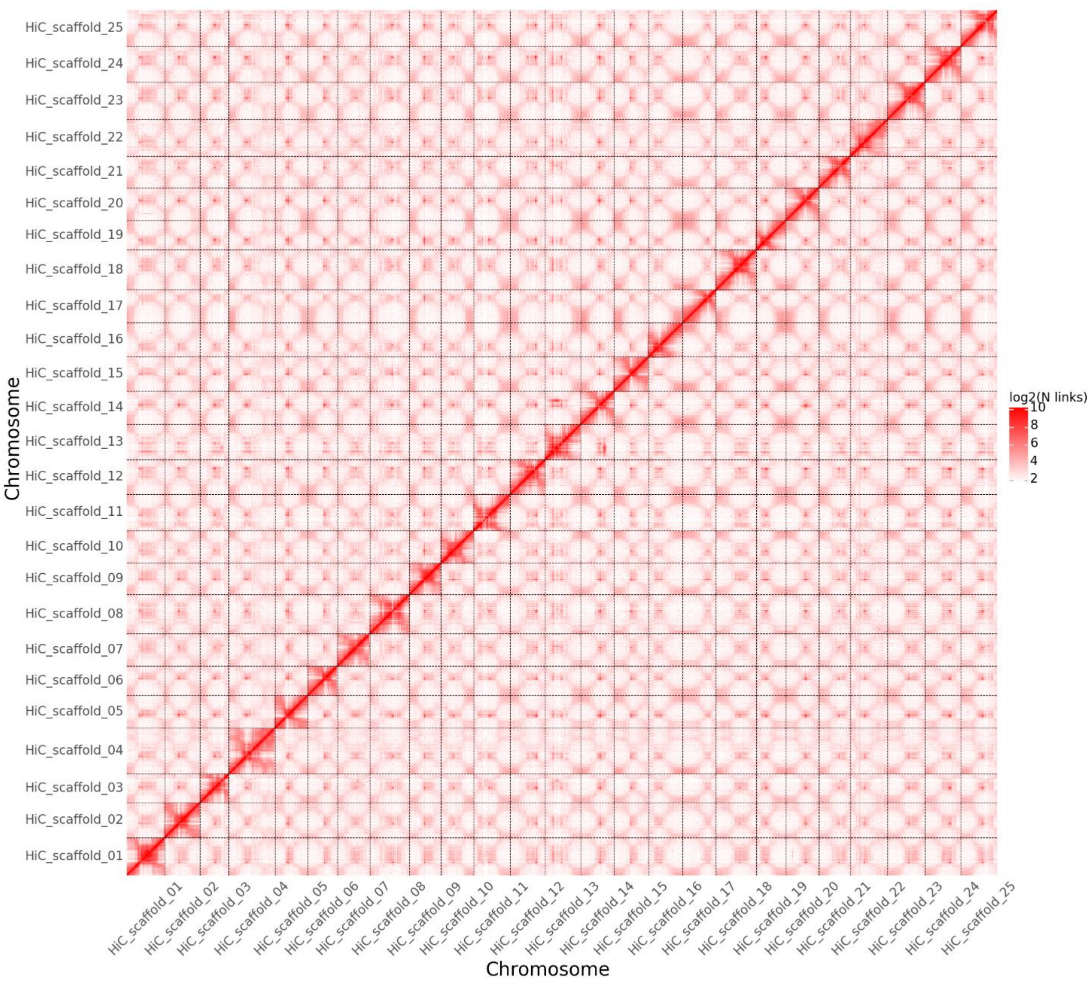

Supplementary Figure 2. Hi-C based clustering of the white lupin chromosomes. Heat map showing the density of Hi-C interactions (calculated by pair-end reads) between contigs, the level of red color indicates the level of density of interactions.

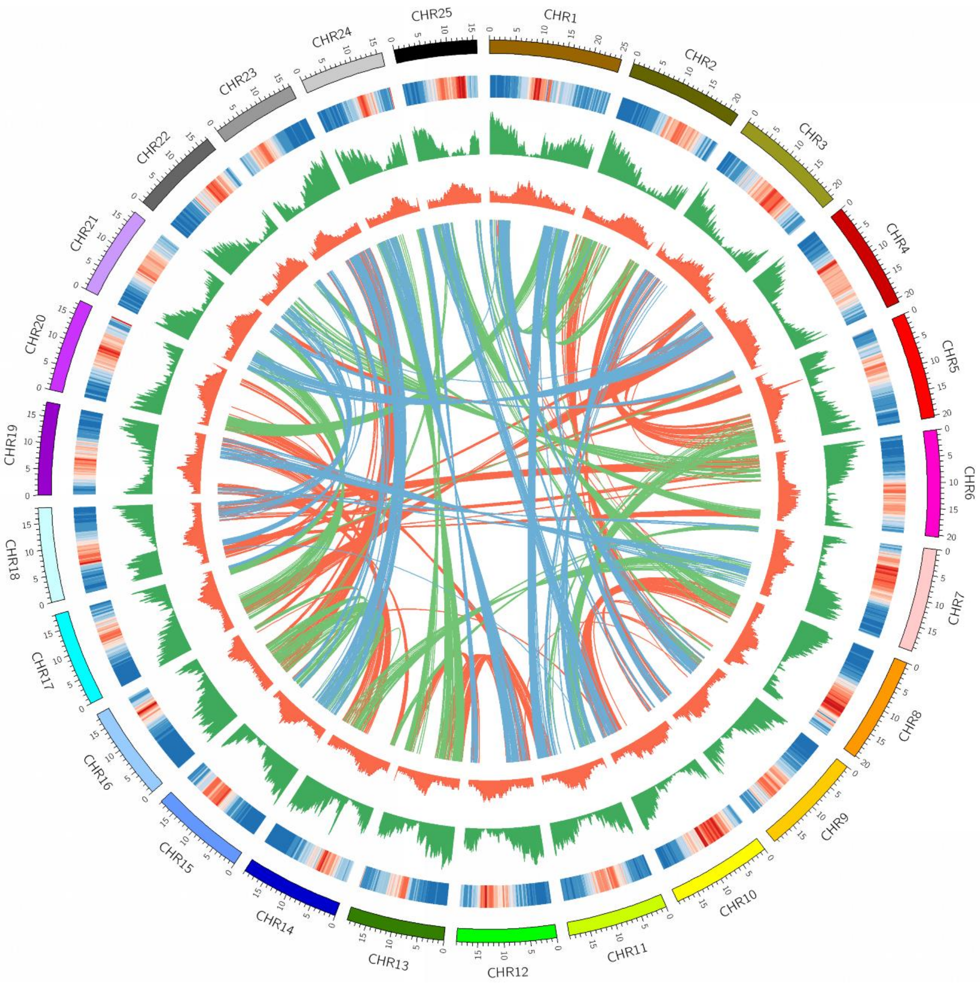

Supplementary Figure 3. Characterization of the white lupin genome with circos plotting. The concentric circles from outermost to innermost show (1) the 25 pseudo-chromosomes, (2) heatmap of the density of repeat sequences (the colors red-white-blue denote high-media-low levels of gene density), (3) histogram of the gene density, and (4) histogram of the GC content. The linking lines indicating the paralogous fragments between either two of the three sub-genomes that were generated from the whole genome triplication event.

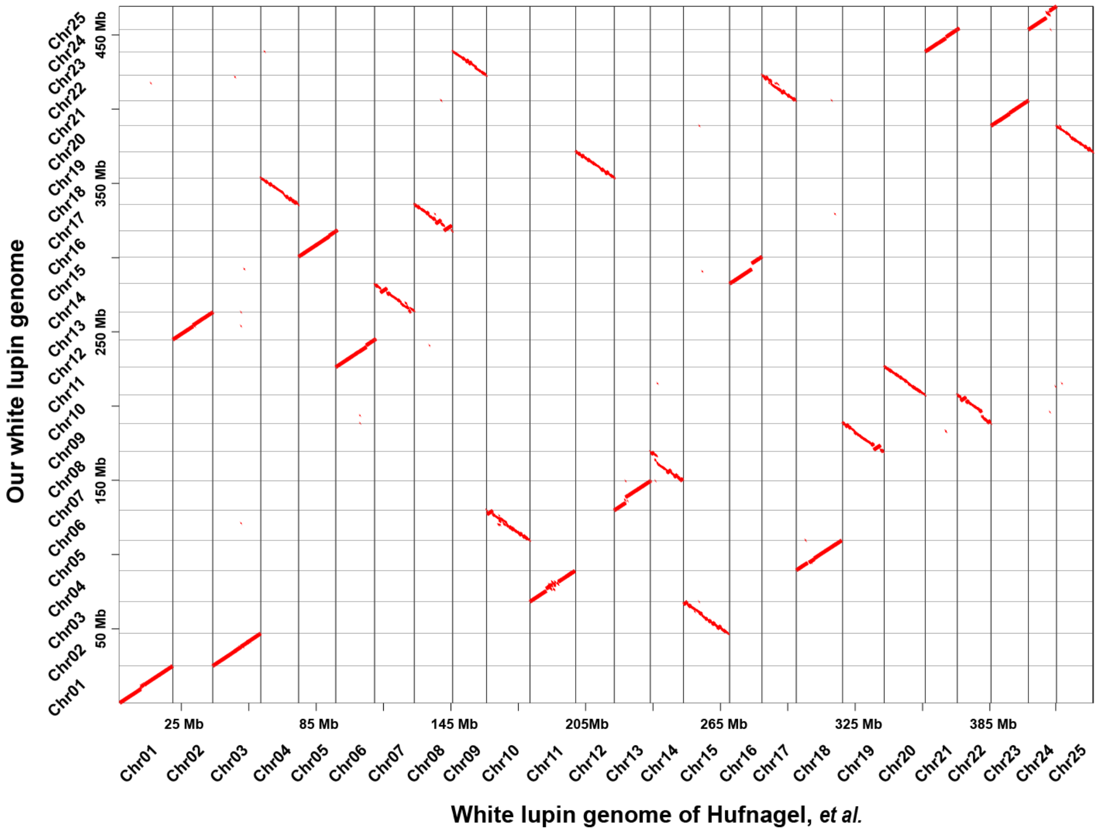

Supplementary Figure 4. Chromosomal synteny comparison between two white lupin genomes. The two genome assemblies of white lupin have different chromosome orders and orientations. The chromosomes 1-25 in our assembly were ordered based on the sequence length of chromosomes. Analysis of chromosomal synteny was performed using the tool Nucmer.

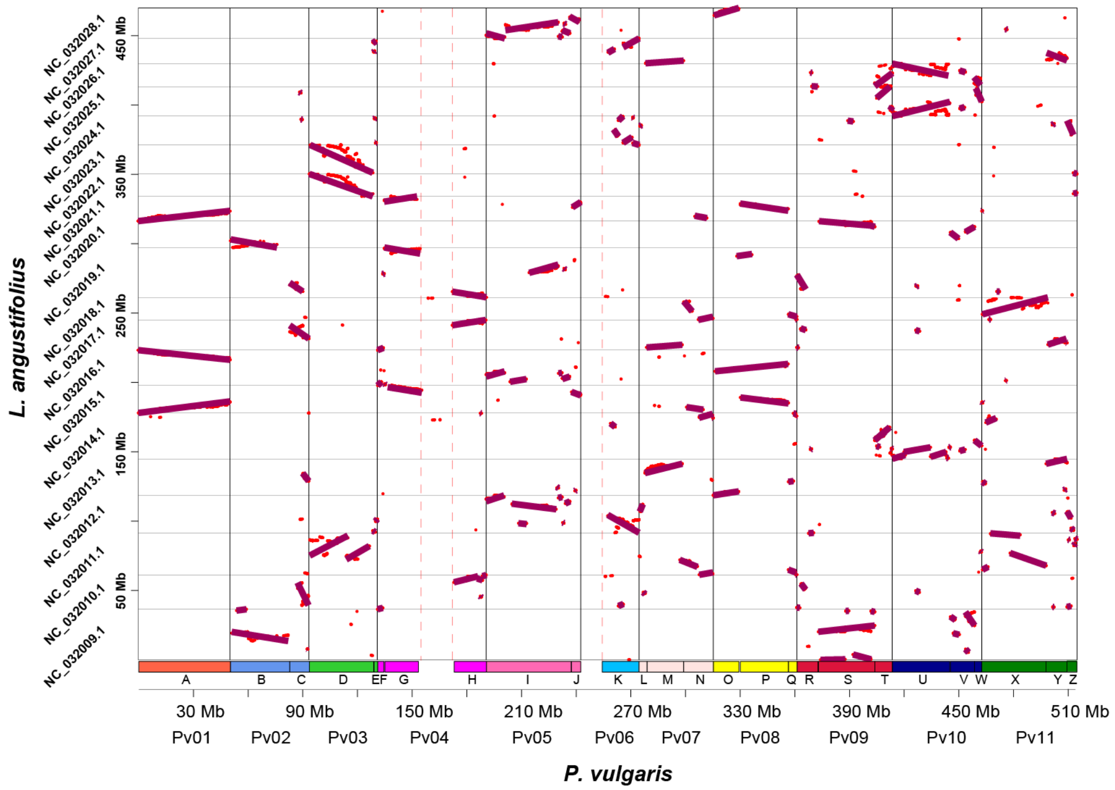

Supplementary Figure 5. The doplots of syntenic gene pairs and syntenic chromosomal fragments between *L. angustifolius* and *P. vulgaris* showing the whole genome triplicated fragments in genome of *L. angustifolius*. Colored bars along the *x*-axis show the 26 genomic blocks defined in genome of *P. vulgaris*. The vertical dash-lines indicate two regions (one is located at the middle of Pv04 and between blocks G and H, the other one is located at the head of Pv06 and before block K) that were lost in *L. angustifolius* genome.

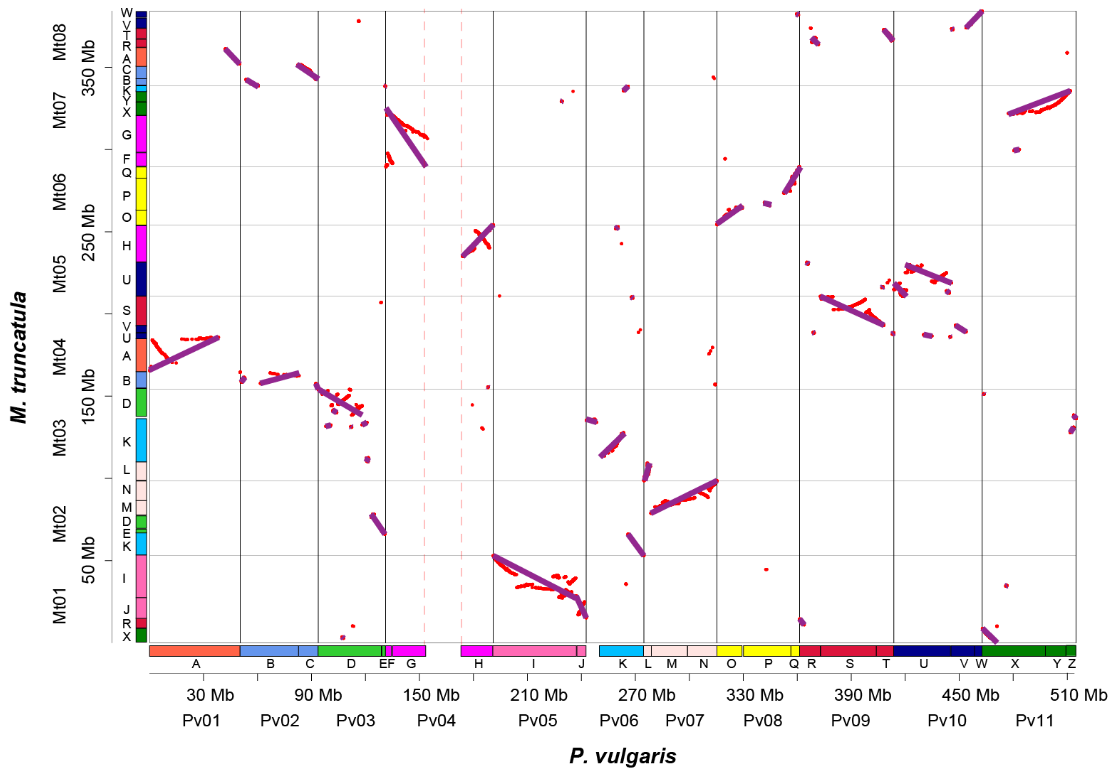

Supplementary Figure 6. The doplots of syntenic gene pairs and syntenic chromosomal fragments between *M. truncatula* and *P. vulgaris* showing the re-arrangement of genomic blocks between the two species. Colored bars along the *x*- and *y*-axis show the distributions of genomic blocks in the two genones. The vertical dash-lines indicate one region (located at the middle of Pv04 and between blocks G and H) that was lost in *M. truncatula* genome.

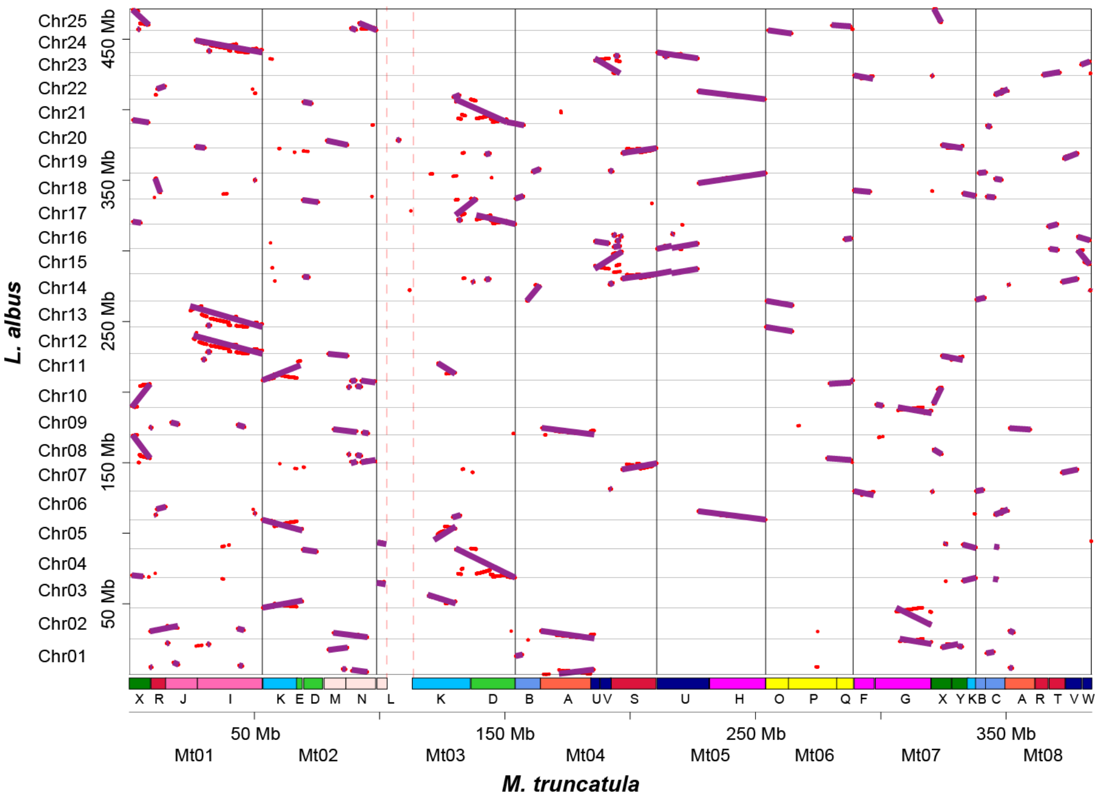

Supplementary Figure 7. The doplots of syntenic gene pairs and syntenic chromosomal fragments between *L. albus* and *M. truncatula* showing the whole genome triplicated fragments in genome of *L. albus*. Colored bars along the *x*-axis show the distribution of genomic blocks in *M. truncatula*. The vertical dash-lines indicate one region (before block K in Mt03) that was lost in *L. albus* genome.

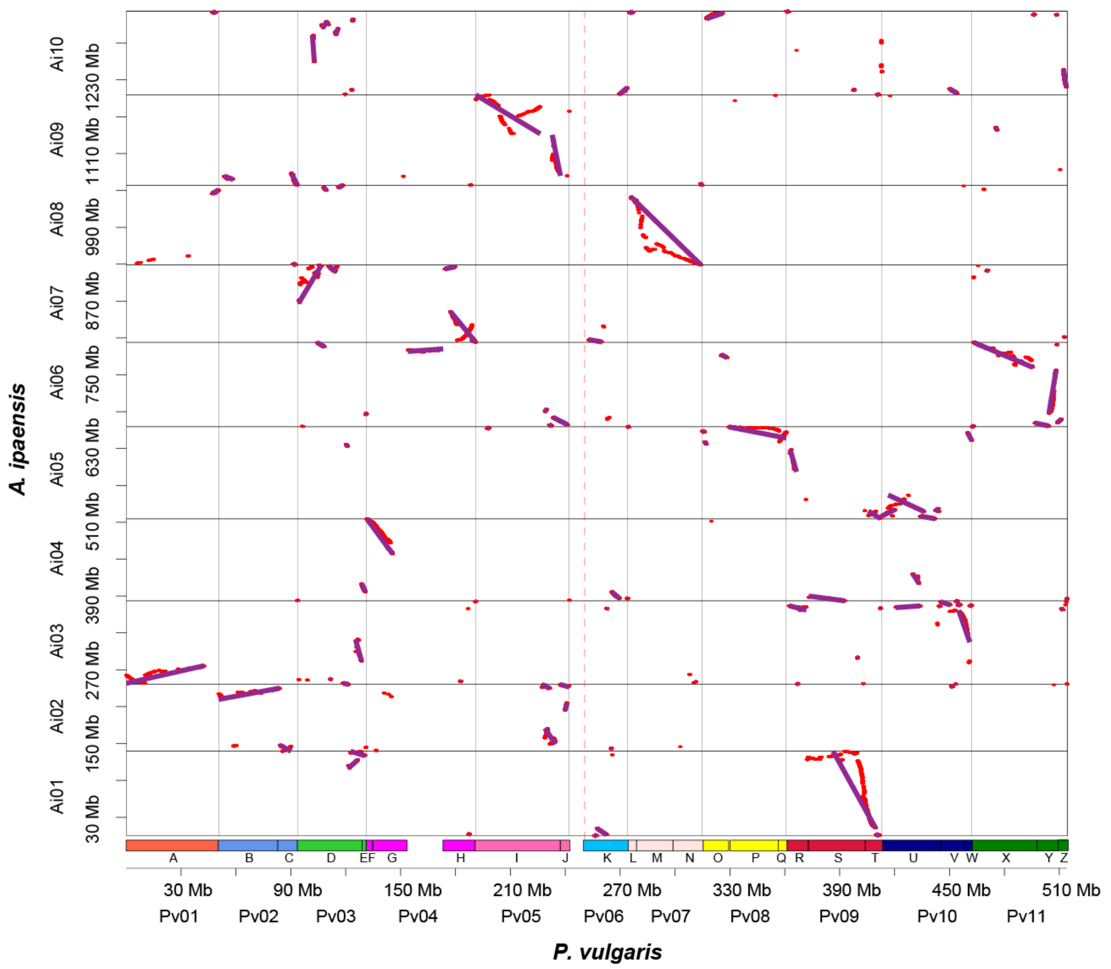

Supplementary Figure 8. The doplots of syntenic gene pairs and syntenic chromosomal fragments between *A. ipaensis* and *P. vulgaris* showing the re-arrangement of genomic blocks between the two species. Colored bars along the *x*-axis show the 26 genomic blocks. The vertical dash-line indicates one region (locate at the head of Pv06 and before block K) that was lost in *A. ipaensis* genome.

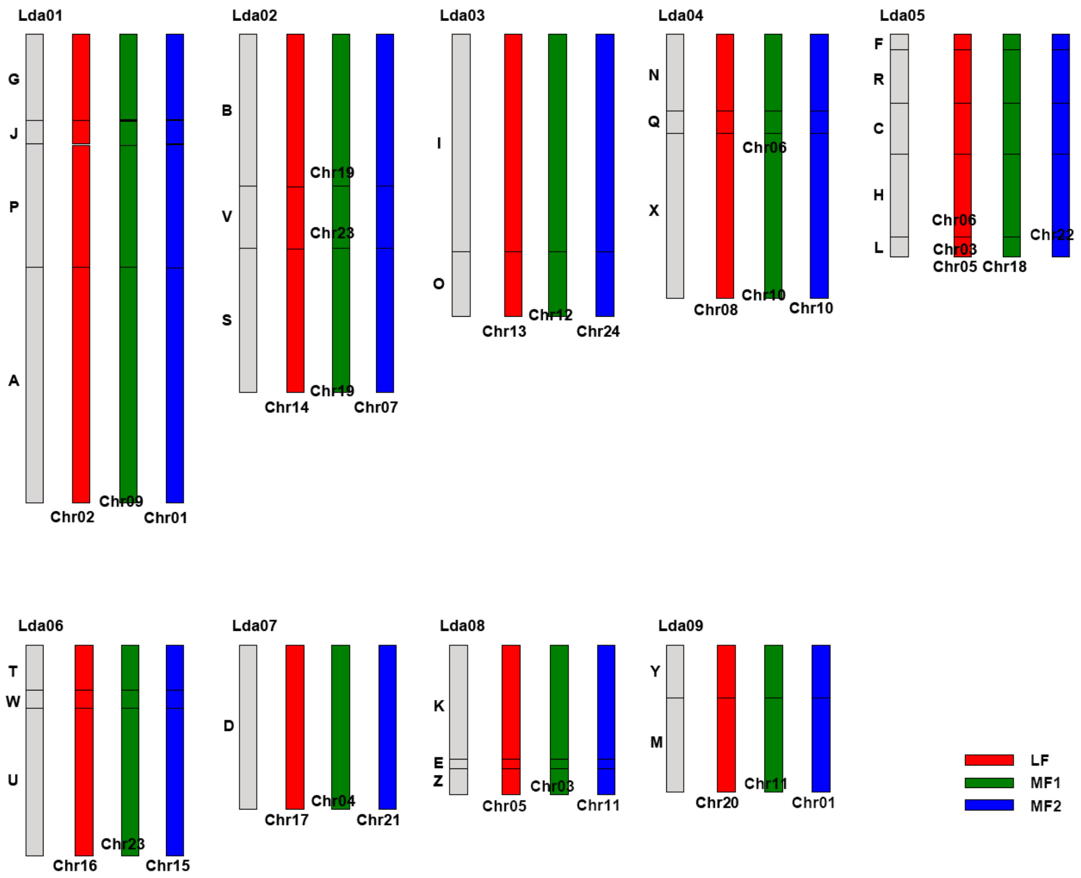

Supplementary Figure 9. The re-arrangement of genomic blocks in the three sub-genomes and their locations in chromosomes of white lupin.

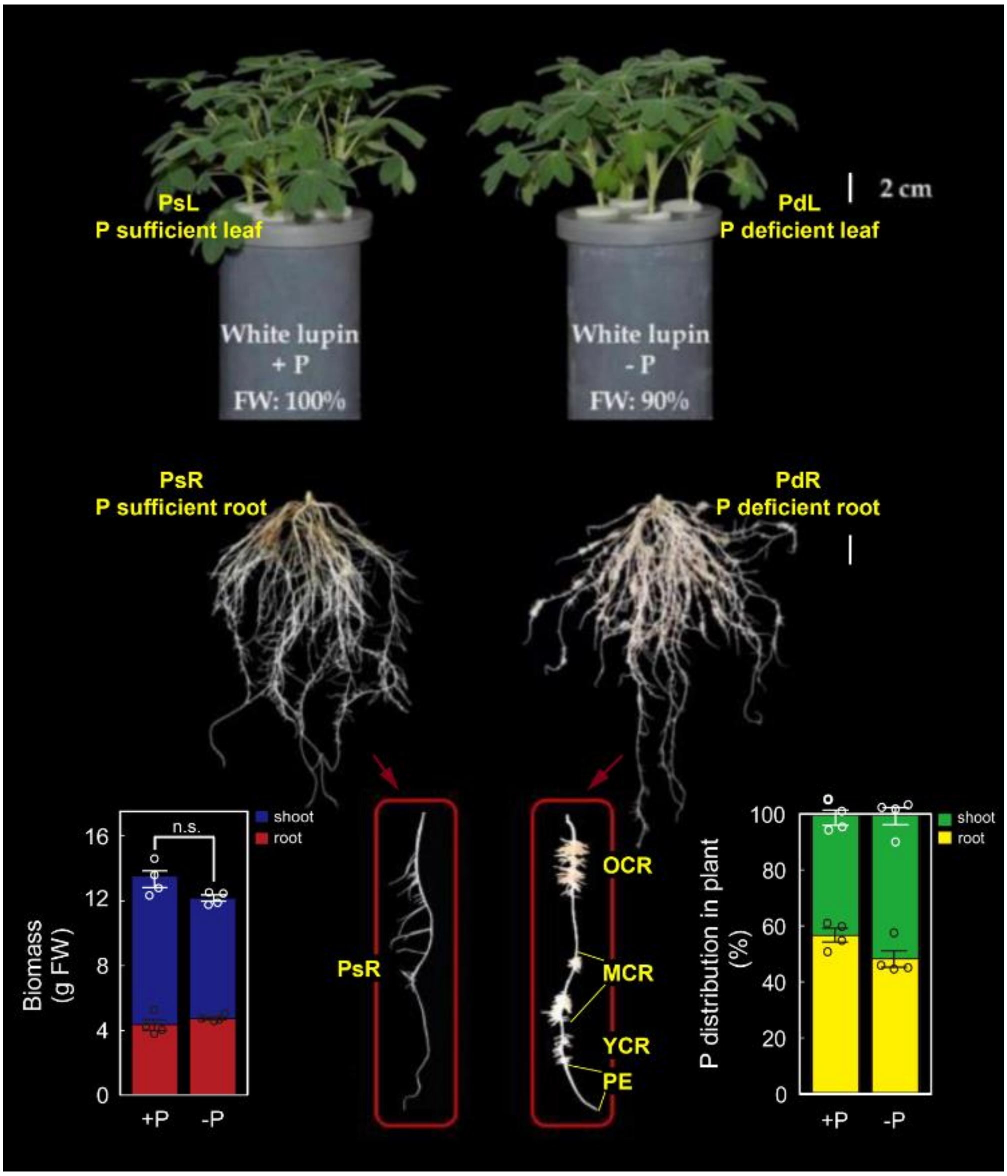

Supplementary Figure 10. Phenotypes of white lupin under P-sufficient and -deficient condition for 4 weeks culture. PsL, leaf of white lupin under P sufficiency; PdL, leaf of white lupin under P deficiency; PsS, stem of white lupin under P sufficiency; PdS, stem of white lupin under P deficiency; PsR, root of white lupin under P sufficiency; PE, pre-emergent zone; YCR, young cluster root; MCR, mature cluster root; OCR, old cluster root. Bars = 2 cm. Error bars indicate s.e.m., n = 4 plants. n.s. indicates no significant difference at $P < 0.05$, according to the unpaired two-sided Student's t-test ($P = 0.1472$). Source data are provided as a Source Data file.

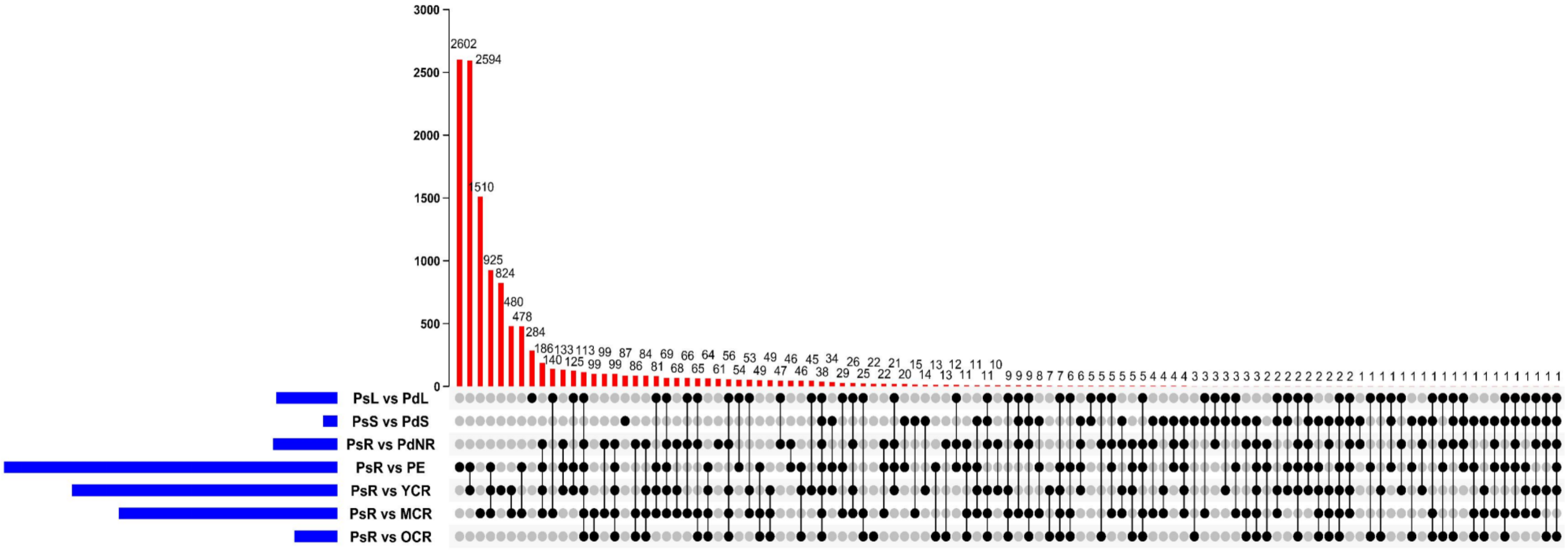

Supplementary Figure 11. Upset graph of differentially expressed genes in comparison of samples from P-deficient and -sufficient treatment. The top bars represent the number of differentially expressed genes (intersection size). The comparison in question is indicated by the dots or connected dots below its respective bar. The left bars indicate the total number of differentially expressed genes for each comparison. PsL, leaves under P sufficiency; PdL, leaves under P deficiency; PsS, stems under P sufficiency; PdS, stems under P deficiency; PsR, roots under P sufficiency. PdNR, normal roots under P deficiency; PE, pre-emergent zone, 2-3 cm behind the root tip of first order laterals; YCR, young cluster root; MCR, mature cluster root; OCR, old cluster root.

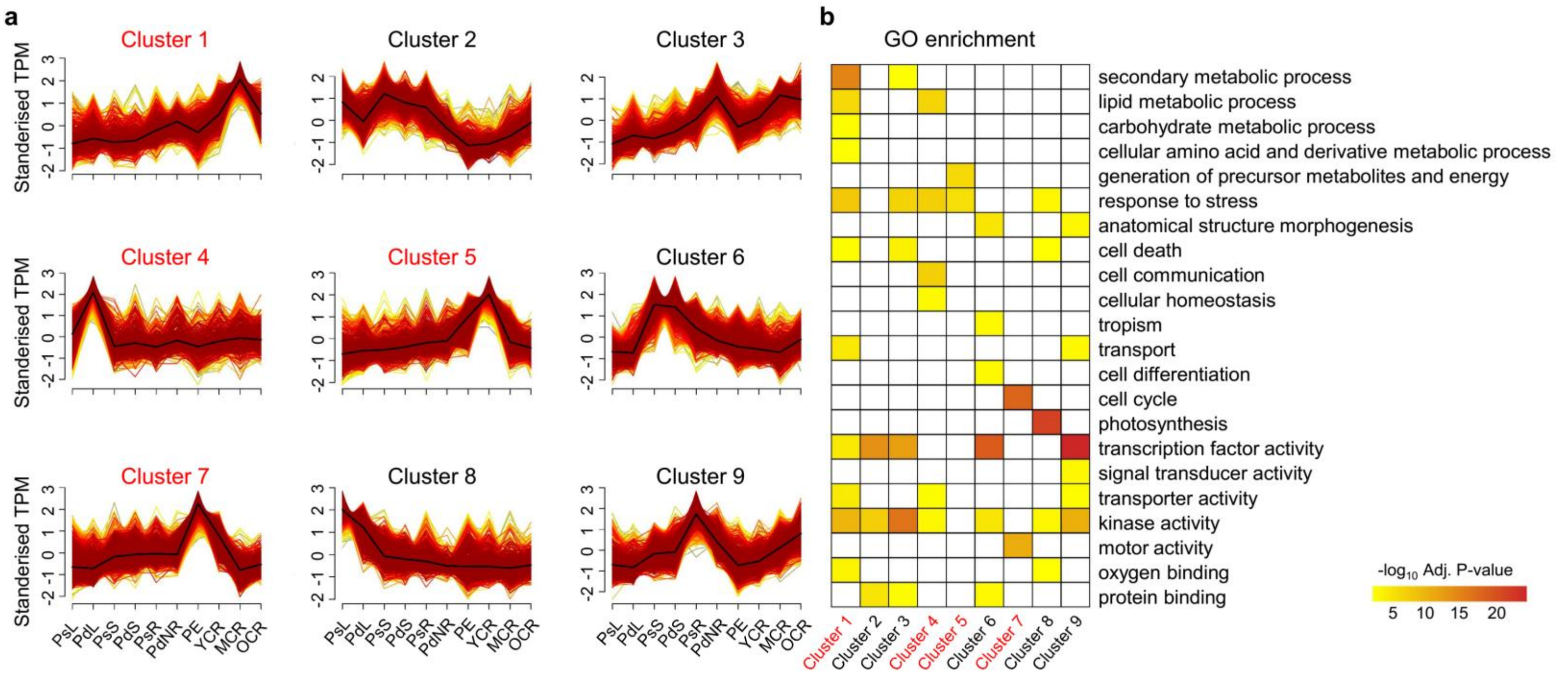

Supplementary Figure 12. Mfuzz clustering and GO enrichment analyses of genes differentially expressed between P-deficient and -sufficient condition. (**a**) Mfuzz clustering showing the transcriptome expression profiles. Nine clusters were identified based on expression levels in ten tissues. PsL, leaves under P sufficiency; PdL, leaves under P deficiency; PsS, stems under P sufficiency; PdS, stems under P deficiency; PsR, roots under P sufficiency. PdNR, normal roots under P deficiency; PE, pre-emergent zone, 2-3 cm behind the root tip of first order laterals; YCR, young cluster root; MCR, mature cluster root; OCR, old cluster root. (**b**) Gene Ontology enrichment among the nine clusters. Yellow to red, significant enrichment, adjust P-value < 0.01; white, not significant. Four main clusters containing the highest number of low-P induced genes were colored in red.

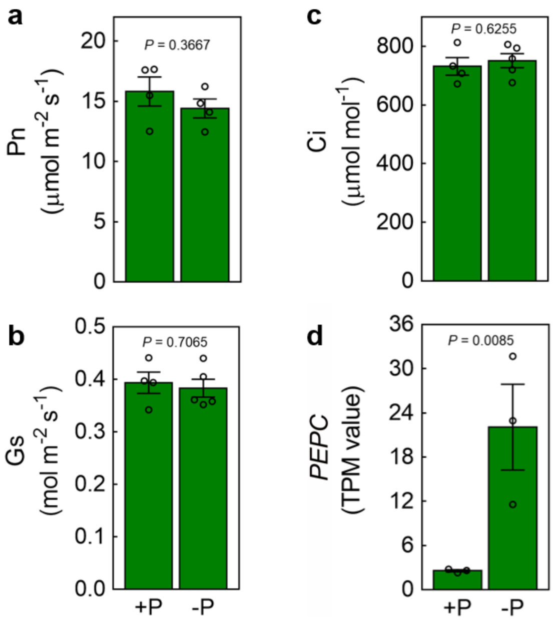

Supplementary Figure 13. Maintenance of photosynthesis of white lupin plant after 4-week of P deficiency. (**a**) Net photosynthesis rate (Pn). Error bars indicate s.e.m., n = 3 plants. (**b**) Stomatal conductance (Gs). n = 4 plants for +P and 5 plants for -P. (**c**) Intercellular CO_2 concentration (Ci). n = 4 plants for +P and 5 plants for -P. (**d**) Expression level of the white lupin gene encoding phosphoenolpyruvate carboxylase (*PEPC*) in P sufficient (+P) and deficient (-P) leaves (*PEPC*). n = 3 plants. *P* value was calculated using the unpaired two-sided Student's t-test. TPM represents the transcript per million fragments mapped. Source data are provided as a Source Data file.

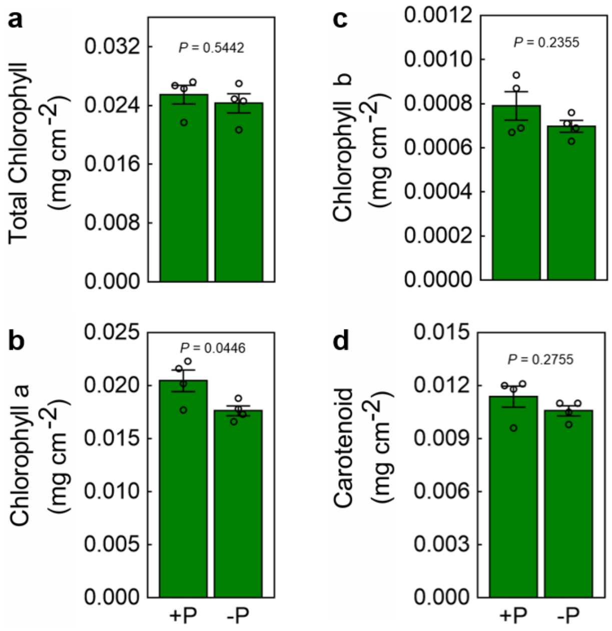

Supplementary Figure 14. Maintenance of leaf pigments after 4-week of P deprivation. (**a**) Total chlorophyll content. (**b**) chlorophyll a. (**c**) chlorophyll b. (**d**) carotenoid in white lupin leaves under P sufficient (+P) or deficient (-P) condition. Error bars indicate s.e.m., n = 4 plants. *P* value was calculated using the unpaired two-sided Student's t-test. Source data are provided as a Source Data file.

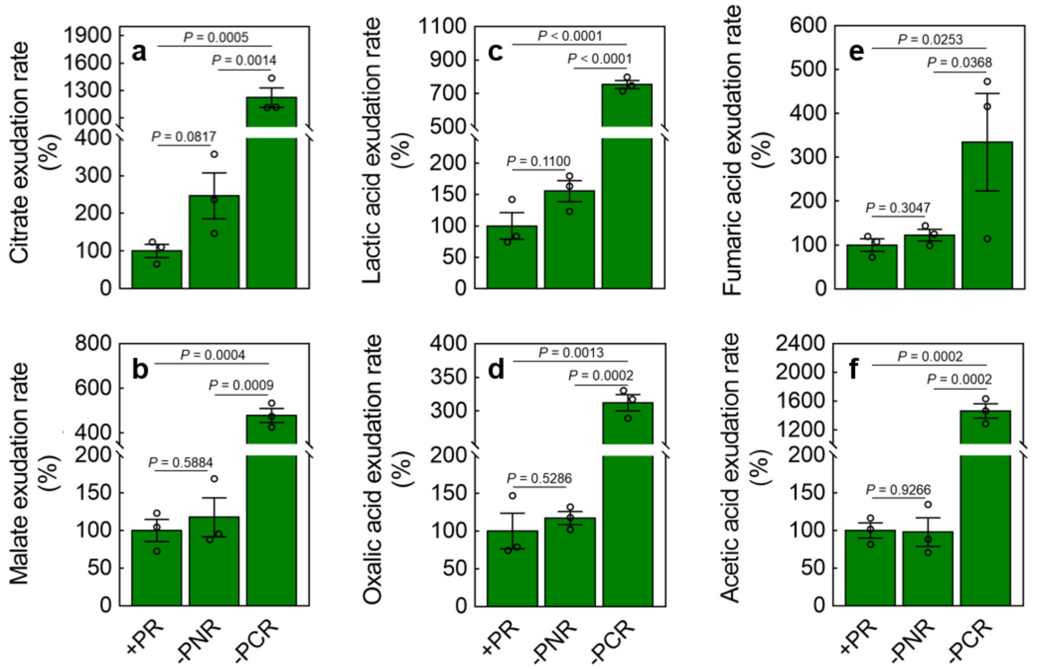

Supplementary Figure 15. Root exudation rate of organic acids from roots of white lupin. (**a** to **f**) Represents the exudation rate of citrate, malate, lactic acid, oxalic acid, fumaric acid and acetic acid, respectively. The plants were grown with or without P. +PR, roots under P sufficiency; -PNR, normal roots under P deficiency; -PCR, cluster roots under P deficiency. Error bars indicate s.e.m., n = 3 plants. *P* value was calculated using the unpaired two-sided Student's t-test. Source data are provided as a Source Data file.

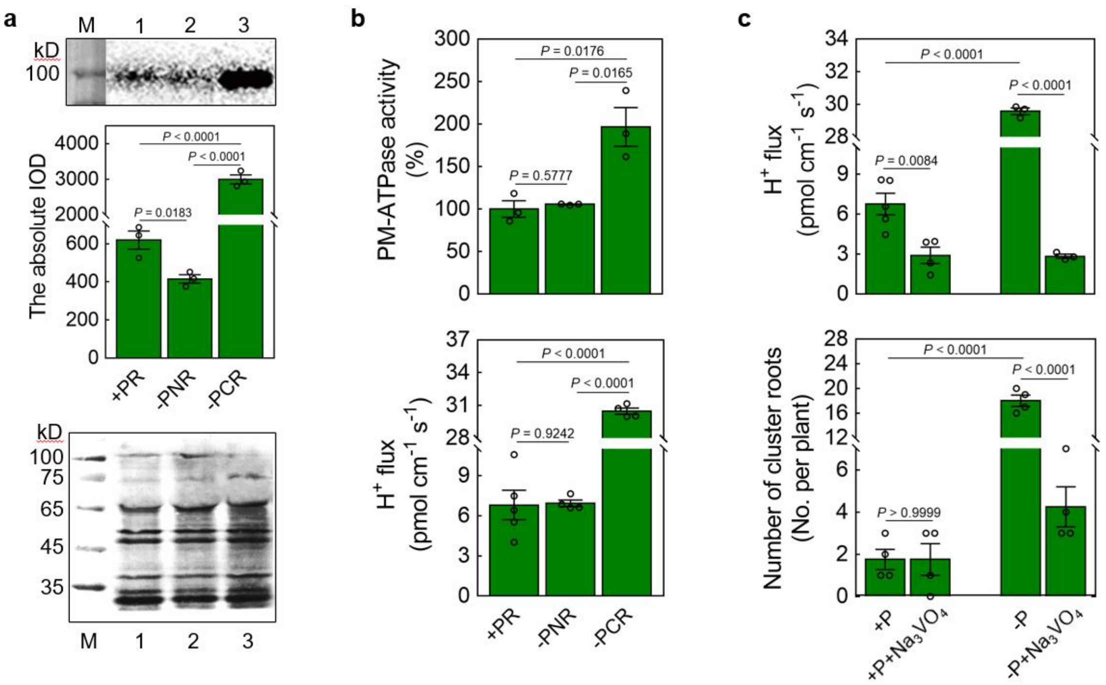

Supplementary Figure 16. Roles of plasma membrane H^+-ATPase in white lupin cluster roots formation and function. (**a**) Immunoblot analysis of PM H^+-ATPase protein abundance. Total membrane proteins extracted from different type roots were separated by SDS-PAGE and analyzed by western blotting with anti-AHA2 antibody. Coomassie Blue staining served as the loading control, and the bands were quantified and expressed as absolute integrated optical density (IOD). Similar results were obtained from three independent biological replicates. (**b**) Hydrolytic activity and H^+ flux rate of PM H^+-ATPase in different types of roots. n = 3 plants for PM ATPase activity and 4 plants for H^+ flux. (**c**) Effects of vanadate (Na_3VO_4, inhibitor of P-type ATPase) on H^+ flux rate and cluster root formation in white lupin under P sufficient and deficient condition. n = 4 plants. Error bars indicate s.e.m., *P* value was calculated using the unpaired two-sided Student's t-test. +PR, P sufficient roots; -PNR, P deficient normal roots; -PCR, P deficient cluster roots; Lane 1, +PR; Lane 2, -PNR; Lane 3, -PCR. Source data are provided as a Source Data file.

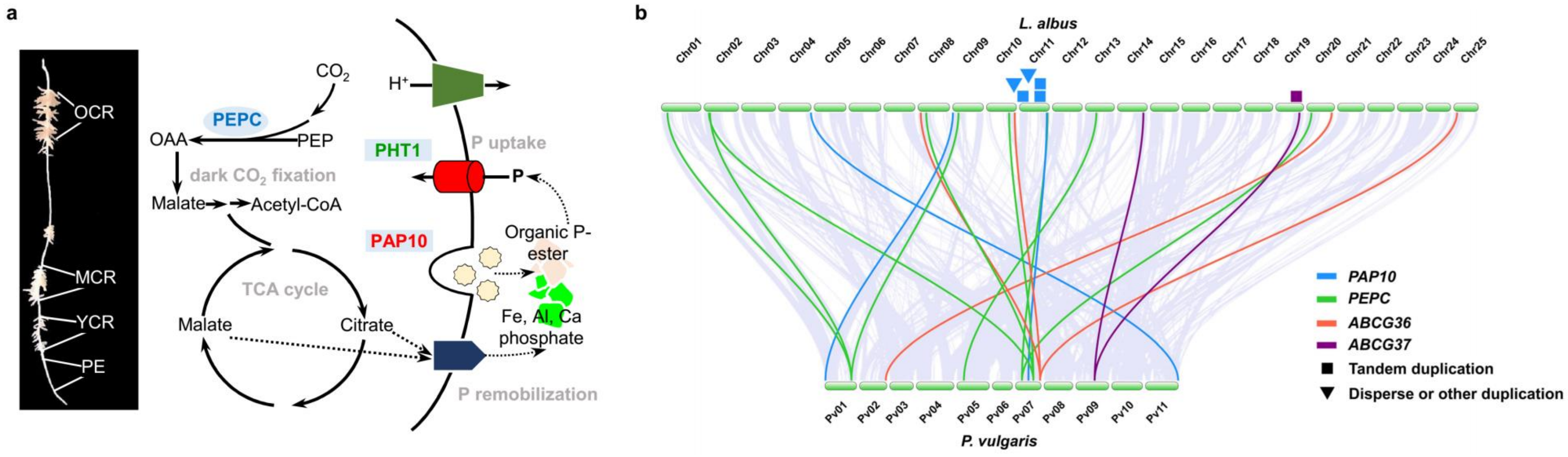

Supplementary Figure 17. The P-use efficiency genes are expanded in white lupin. (a) Overview of high phosphorus remobilization and aquisition by white lupin cluster roots. Organic acids (e.g. malate and citrate), protons and purple acid phosphatases released by cluster roots are capable of freeing inorganically and organically bound P in soil, which then acquired by high affinity phosphate transporter. Therein, malate and citrate production requires TCA cycle and dark CO_2 fixation. *PEPC*, *PHT1* and *PAP10* represent genes encoding phosphoenolpyruvate carboxylase, phosphate transporter 1 and purple acid phosphatase 10, respectively. PEP, phosphoenolpyruvate; OAA, oxaloacetate. (**b**) The syntenic homologous genes between genomes of *P. vulgaris* and *L. albus*. All syntenic gene pairs between the two genomes are linked by lines colored in light grey, while three groups of genes *PAP10*, *PEPC*, *ABCG36*, and *ABCG37* were linked by lines colored in blue, green, orange, and purple, respectively. For the *PAP10* genes, there are three and two of them have been expanded through tandem duplication (red square) and dispersed duplication (red triangle), respectively.

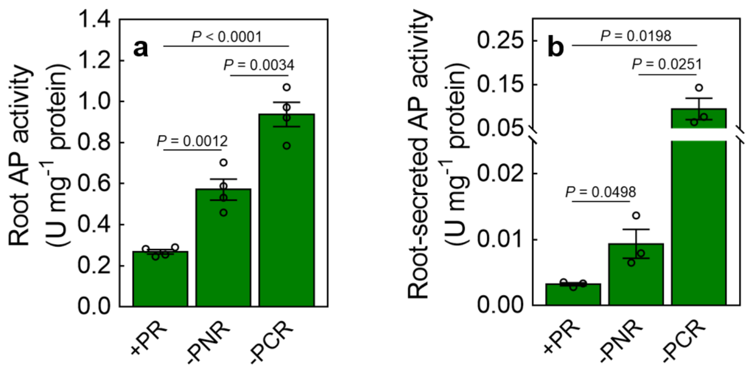

Supplementary Figure 18. Activity of acid phosphatase (AP) in roots and root exudates of white lupin. (**a**) AP activity in roots. n = 4 plants. (**b**) AP activity in root exudates of plants grown with or without phosphorus. n = 3 plants. +PR, roots under P sufficiency; -PNR, normal roots under P deficiency; -PCR, cluster roots under P deficiency. Error bars indicate s.e.m., *P* value was calculated using the unpaired two-sided Student's t-test. Source data are provided as a Source Data file.

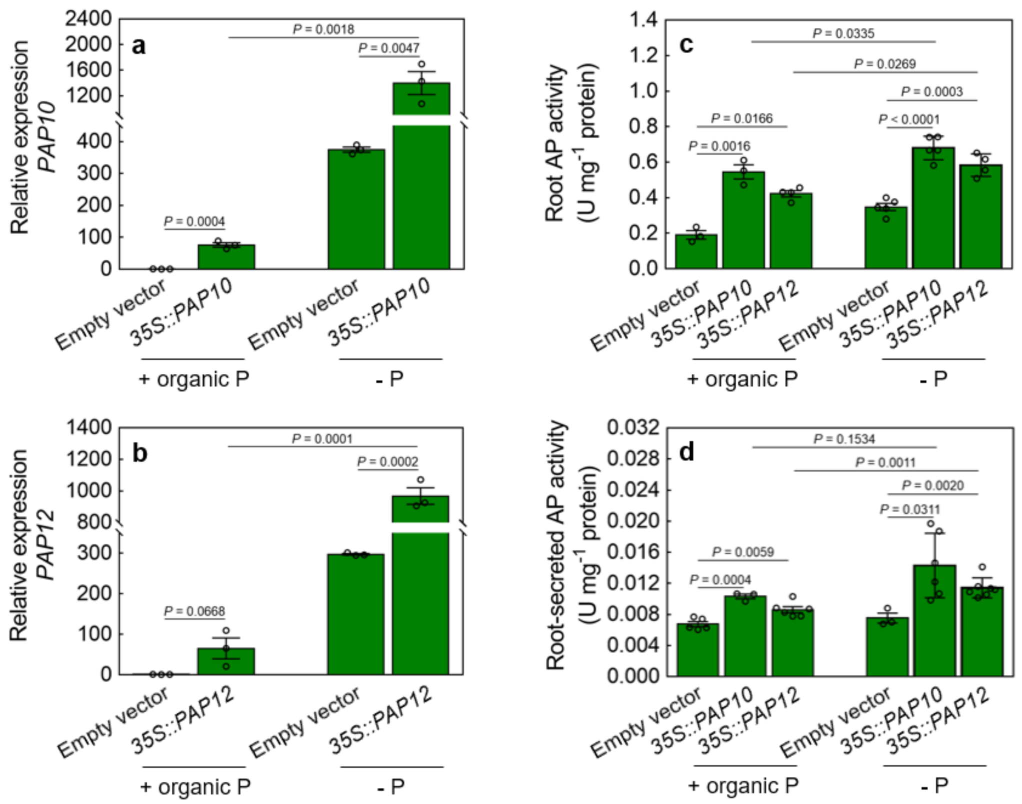

Supplementary Figure 19. Functional analysis of *PAP10* and *PAP12* genes in white lupin by *Agrobacterium rhizogenes*-based hairy root transformation system. (**a** and **b**) Expression levels of *PAP10* and *PAP12* in *35S::PAP10* and *35S::PAP12* transgenic hairy roots relative to the control transformed with empty vector. n = 3 plants. (**c**) Acid phosphatase activities of white lupin in root tissue. n = 3 plants for organic P treatment and 5 plants for -P treatment. (**d**) Acid phosphatase activities of white lupin in root exudates. n = 5 plants for organic P treatment and 6 plants for -P treatment. Error bars indicate s.e.m., *P* value was calculated using the unpaired two-sided Student's t-test. Source data are provided as a Source Data file.

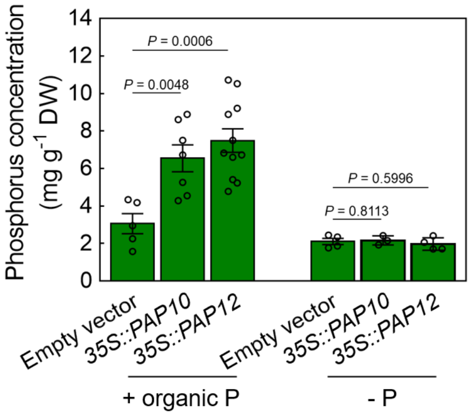

Supplementary Figure 20. P content in hairy roots of white lupin. Plants were transformed with empty vector, *35S::PAP10* and *35S::PAP12* under P deficiency with or without phytate (main form organic P in soil). –P, 1/2 MS basal salt without phosphate; + organic P, 1/2 MS basal salt without phosphate but added with phytate. Error bars indicate s.e.m., n = 5 plants for empty vector, 7 plants for *35S::PAP10*, 11 plants for *35S::PAP12* under organic P condition and 4 plants for -P condition. *P* value was calculated using the unpaired two-sided Student's t-test. Source data are provided as a Source Data file.

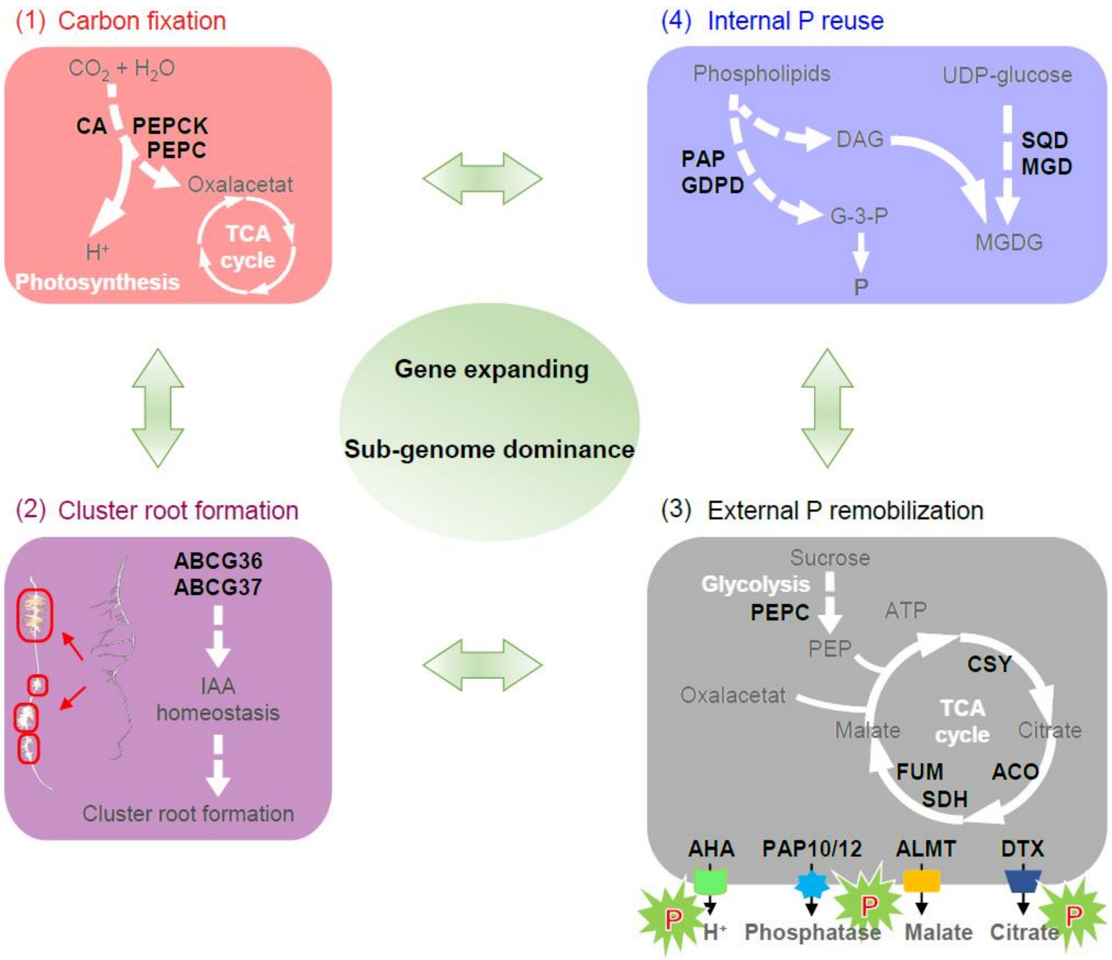

Supplementary Figure 21. The proposed model of four main pathways for low-phosphorus adaption in white lupin. (1) Carbon fixation. The derived carbohydrate and carboxylate from photosynthesis and/or non-photosynthetic carbon fixation provide roots including cluster roots with the energy and C-skeleton sources. (2) Cluster root formation. With the energy and C-skeleton sources prepared from shoot and root carbon fixation, *ABCG36* and *ABCG37* promote cluster-root-forming by affecting IAA homeostasis in root tip. (3) External P remobilization. The formed cluster roots effectively solubilize inorganic and organic P bound in soil by exuding H^+ (proton), citrate, malate and phosphatase into rhizosphere. In this biological process, the carboxylate metabolized from glycolysis is incorporated in the tricarboxylic acid cycle (TCA cycle) to generate citrate and malate; the organic acids are then pumped into soil by ALMT and DTX to remobilize Al-P and Fe-P; H^+ was pumped out by proton pump (AHA) to remobilize Ca-P; the secreted phosphatase PAP10 and PAP12 can remobilize organic P in soil. (4) Internal P reuse. Under P deficient conditions, phospholipids are partially degraded by a cascade of enzymes including PAP, GDPD, SQD and MGD to cope with internal P shortage; the derived P from phospholipid degradation is subsequently engaged in the maintenance of photosynthesis as well as other bioprocesses. PAP shows the dual roles in P use (external P remobilization and internal P reuse). All the co-opted four bioprocesses were mediated by polyploidization and/or protein phosphorylation. CA, carbonic

anhydrase; PEPCK, phosphoenolpyruvate carboxylase kinase; PEPC, phosphoenolpyruvate carboxylase; ABCG36, ATP-binding cassette G36; ABCG37, ATP-binding cassette G37;CSY, citrate synthase; ACO, aconitase; SDH, succinate dehydrogenase; FUM, fumarase; AHA, proton pump (PM H^+-ATPase); PAP, purple acid phosphatase, phosphatidic acid phosphatase; ALMT, aluminum-activated malate transporter; DTX, detoxifying efflux carrier (MATE efflux family protein); GDPD, glycerophosphodiester phosphodiesterase; SQD, sulfoquinovosyltransferase; MGD, monogalactosyl diacylglycerol synthase.
