# Supplementary material for: The genome evolution and low-phosphorus adaptation in white lupin
Source: Nat Commun. 2020 Feb 26;11:1069. doi: 10.1038/s41467-020-14891-z (PMC7044338; doi:10.1038/s41467-020-14891-z)
Supplement: Supplementary file 3 — Reporting Summary [file 41467_2020_14891_MOESM3_ESM.pdf]

nature research

Corresponding author(s): Weifeng Xu, Feng Cheng, Qian Zhang, Tianyu Xia

Last updated by author(s): 7/2/2020

Reporting Summary

Nature Research wishes to improve the reproducibility of the work that we publish. This form provides structure for consistency and transparency in reporting. For further information on Nature Research policies, see Authors & Referees and the Editorial Policy Checklist .

Statistics

For all statistical analyses, confirm that the following items are present in the figure legend, table legend, main text, or Methods section.

n/a	Confirmed	
☐	☒	The exact sample size (*n*) for each experimental group/condition, given as a discrete number and unit of measurement
☐	☒	A statement on whether measurements were taken from distinct samples or whether the same sample was measured repeatedly
☐	☒	The statistical test(s) used AND whether they are one- or two-sided<br>*Only common tests should be described solely by name; describe more complex techniques in the Methods section.*
☐	☒	A description of all covariates tested
☒	☐	A description of any assumptions or corrections, such as tests of normality and adjustment for multiple comparisons
☐	☒	A full description of the statistical parameters including central tendency (e.g. means) or other basic estimates (e.g. regression coefficient) AND variation (e.g. standard deviation) or associated estimates of uncertainty (e.g. confidence intervals)
☐	☒	For null hypothesis testing, the test statistic (e.g. *F*, *t*, *r*) with confidence intervals, effect sizes, degrees of freedom and *P* value noted<br>*Give P values as exact values whenever suitable.*
☒	☐	For Bayesian analysis, information on the choice of priors and Markov chain Monte Carlo settings
☒	☐	For hierarchical and complex designs, identification of the appropriate level for tests and full reporting of outcomes
☒	☐	Estimates of effect sizes (e.g. Cohen's *d*, Pearson's *r*), indicating how they were calculated

Our web collection on statistics for biologists contains articles on many of the points above.

Software and code

Policy information about availability of computer code

Data collection	GE ImageQuant TL Image Analysis Software Set IQTL8.1
Data analysis	The softwares used in this manuscript include GraphPad Prism 7, Microsoft Office 2016, Adobe Photoshop CC, Adobe Illustrator CC, Hisat2 (v2.1.0), R (v3.6.1), MEGA-X (v10.0.5), Gel-Pro Analyzer software (v4.0), SAS (v9.1).<br>The R packages used include DESeq (v1.10.1), ComplexHeatmap (v2.2.0).<br>JellyFish (v2.2.3), Canu (v1.8), GenomeScope (v1.0), Bandage (0.8.1), JuicerBox (v1.5.1), Juicer (v1.6.2), Pilon (v1.23), Bowtie2 (2.3.5), RepeatModeler (v1.0.11), RepeatMasker (v4.0.8), RepeatProteinMask (v3.3.0), SynOrths (v1.0), Genewise (v2.4.1), Augustus (v3.2.1), GeneScan (v3.7), Stringtie (v1.3.6), TransDecode (v5.0.2), Maker (v2.31.10), MUSCLE (3.28.0).

For manuscripts utilizing custom algorithms or software that are central to the research but not yet described in published literature, software must be made available to editors/reviewers. We strongly encourage code deposition in a community repository (e.g. GitHub). See the Nature Research guidelines for submitting code & software for further information.

Data

Policy information about availability of data

All manuscripts must include a data availability statement. This statement should provide the following information, where applicable:

- Accession codes, unique identifiers, or web links for publicly available datasets
- A list of figures that have associated raw data
- A description of any restrictions on data availability

This Whole Genome Shotgun project has been deposited at DDBJ/ENA/GenBank under the accession JAAEJY000000000. The version described in this paper is version JAAEJY010000000 [https://www.ncbi.nlm.nih.gov/nuccore/JAAEJY000000000]. The white lupin genome datasets are also freely available through the link: http://brassicadb.org/brad/pub/genomes/Lalbus/. The two public mRNA-seq data sets in white lupin under P deficiency were downloaded from the NCBI database under accession SRA145661 [https://www.ncbi.nlm.nih.gov/sra/?term=SRA145661] and GSE31132 [https://www.ncbi.nlm.nih.gov/geo/query/acc.cgi?acc=GSE31132]. Genome datasets of A. thaliana were downloaded from the TAIR database (TAIR10; http://www.arabidopsis.org/index.jsp). Genome sequences of

other Legume species were downloaded from the legume information system (https://legumeinfo.org/) as well as the Phytozome database (https://phytozome.jgi.doe.gov/). Data supporting the findings of this work are available within the paper and its Supplementary Information files. A reporting summary for this article is available as a Supplementary Information file. The datasets generated and analyzed in the current study are available from the corresponding author on reasonable request. The source data underlying Figs. 1b, 3, 5a-c, Supplementary Figure 10 and Supplementary Figures 14-20 are provided as a Source Data file.

Field-specific reporting

Please select the one below that is the best fit for your research. If you are not sure, read the appropriate sections before making your selection.

[x] Life sciences [ ] Behavioural & social sciences [ ] Ecological, evolutionary & environmental sciences

For a reference copy of the document with all sections, see nature.com/documents/nr-reporting-summary-flat.pdf

Life sciences study design

All studies must disclose on these points even when the disclosure is negative.

Sample size	No sample size calculations were performed. Sample size was determined to be adequate based on the magnitude and consistency of measurable differences between groups. We performed statistical tests with obtained data to ensure that a sample size with 3-11 plants is sufficient to draw conclusions about significance. We have performed ten mRNA-seq for three tissues with 3 biological replicates for differential gene expression analysis.
Data exclusions	No data were excluded from the analyses.
Replication	All experiments were performed at independent experimental days and showed similar tendencies.
Randomization	Samples were randomly allocated into experimental groups.
Blinding	Investigators were blinded.

Reporting for specific materials, systems and methods

We require information from authors about some types of materials, experimental systems and methods used in many studies. Here, indicate whether each material, system or method listed is relevant to your study. If you are not sure if a list item applies to your research, read the appropriate section before selecting a response.

Materials & experimental systems

n/a	Involved in the study	
[ ]	[x]	Antibodies
[x]	[ ]	Eukaryotic cell lines
[x]	[ ]	Palaeontology
[x]	[ ]	Animals and other organisms
[x]	[ ]	Human research participants
[x]	[ ]	Clinical data

Methods

n/a	Involved in the study	
[x]	[ ]	ChIP-seq
[x]	[ ]	Flow cytometry
[x]	[ ]	MRI-based neuroimaging

Antibodies

Antibodies used	The following antibodies were used in this experiment: anti-PMA2 polyclonal antibody against the conserved catalytic domain of the plasma membrane H+-ATPase of Arabidopsis (AHA2) raised in rabbits were made and kindly supplied by Toshinori Kinoshita from Kyushu University, Japan; anti-actin (plant) monoclonal antibody produced in mouse, Sigma, A0480-200UL, Lot #062M7821V. The antibodies were used at 1:2000 and 1:10000 respectively.
Validation	Anti-PMA2 polyclonal antibody is validated in previous publications for Arabidopsis (Hayashi et al. Biochemical Characterization of In Vitro Phosphorylation and Dephosphorylation of the Plasma Membrane H + -ATPase. Plant & Cell Physiology. 51: 1186-1196 (2010), and by the Western Blot techniques.
