# Supplementary material for: The genome evolution and low-phosphorus adaptation in white lupin
Source: Nat Commun. 2020 Feb 26;11:1069. doi: 10.1038/s41467-020-14891-z (PMC7044338; doi:10.1038/s41467-020-14891-z)
Supplement: Supplementary file 4 — Description of Additional Supplementary Files [file 41467_2020_14891_MOESM4_ESM.docx]

**Descriptions of Additional Supplementary Files**

File name: Supplementary Data 1
Description: The significantly enriched GO terms in multiple-copy geneset comparing to that in single-copy geneset from orthologous genes of white lupin to *A. thaliana. P* value was calculated using the one-sided Fisher's exact test.

File name: Supplementary Data 2
Description: The significantly enriched GO terms in multiple-copy geneset comparing to that in single-copy geneset from orthologous genes of white lupin to *L. angusfolius. P* value was calculated using the one-sided Fisher's exact test.

File name: Supplementary Data 3
Description: The significantly enriched GO terms in white lupin in the multiple-copy genes comparing to single-copy genes from synteny gene pairs to *P. vulgaris. P* value was calculated using the one-sided Fisher's exact test.

File name: Supplementary Data 4
Description: The significantly enriched GO terms in *L. angustifolius* in the multiple-copy genes comparing to single-copy genes from synteny gene pairs to *P. vulgaris. P* value was calculated using the one-sided Fisher's exact test.

File name: Supplementary Data 5
Description: The significantly enriched GO terms in tandem duplicated genes of white lupin. *P* value was calculated using the one-sided Fisher's exact test.

File name: Supplementary Data 6
Description: The significantly enriched GO terms in tandem duplicated genes of *L. angustifolius. P* value was calculated using the one-sided Fisher's exact test.

File name: Supplementary Data 7
Description: The significantly enriched GO terms in transpose duplicated genes of white lupin *. P* value was calculated using the one-sided Fisher's exact test.

File name: Supplementary Data 8
Description: The significantly enriched GO terms in transpose duplicated genes of *L. angustifolius*. *P* value was calculated using the one-sided Fisher's exact test.

File name: Supplementary Data 9
Description: PUE (P-use efficiency) genes in *A. thaliana* grouped by biological process or molecular function.

File name: Supplementary Data 10
Description: Orthologs of the *A. thaliana* PUE genes in white lupin.

File name: Supplementary Data 11
Description: Statistics of PUE genes in *A. thanliana* and white lupin categorized by biological process or molecular function.

File name: Supplementary Data 12
Description: Expression profiles of the differentially expressed PUE genes in leaves, stems and roots of white lupin under P deficiency compared to those from P-sufficient condition.

File name: Supplementary Data 13
Description: List of key white lupin PUE genes involved in low-P adaptive pathways

File name: Supplementary Data 14

Description: Expansion of key white lupin PUE genes involved in low-P adaptive pathways

File name: Supplementary Data 15.

Description: Locations of *PAP10* genes in the white lupin genome.

File name: Supplementary Data 16

Description: Phosphoproteomic profiles of the white lupin plants under P-sufficient and -deficient condition. *P* value was calculated using the two-sided Fisher's exact test.
