# Supplementary material for: The genome evolution and low-phosphorus adaptation in white lupin
Source: Nat Commun. 2020 Feb 26;11:1069. doi: 10.1038/s41467-020-14891-z (PMC7044338; doi:10.1038/s41467-020-14891-z)
Supplement: Supplementary file 2 — Peer Review File [file 41467_2020_14891_MOESM2_ESM.pdf]

Reviewers' comments:

Reviewer #1 (Remarks to the Author):

Xu et al. “Co-evolution of multiple pathways to low phosphorus adaptation in white lupin” reports a version of the white lupin genome of cv Amiga. White lupin is of particular interest because it is a protein-rich crop that has developed a peculiar adaptation to soil phosphorus deficiency. The authors present results on the structure, evolution and expression of the white lupin genome, with a focus on P acquisition- and metabolism-related genes.
The manuscript brings interesting results for example on sub-genome dominance and link with TE density in white lupin, or on paralogs’ retention, but overall, the text is too long and should be deeply re-written in order to better convey the results. The wording is often unclear, redundant or not precise enough. The terms P-use or high-P-use are in most cases inappropriate in the paper and should be replaced by P use efficiency, or P acquisition or uptake, or low-P adaptation.
The title does not convey the general content of the paper.
The abstract should be revised and checked. White lupin is not the only crop that develops cluster roots (see for example Plant and Soil (2005) 274:101–125). Lines 33-35 are not clear: the notion of sub-genome dominance is not straightforward and “the biased distribution of TE” is not clear enough. Express in a clearer way that on average, the genic expression of the different subgenomes vary according to the TE density of these subgenomes. Lines 35-38 seems contradictory. Have P-related genes been expanded more through the triplication event, or through tandem duplication? The notion of transposed duplication is unclear and is not supported by the results. Avoid using etc. at Line 39 and at other places in the text but rather be more specific or give a reference. Line 44, what is the enigma of genome evolution? More simply write “gives insights into genome evolution”.
The introduction should be re-written to be more specific and accurate. It focuses mainly on the phosphorus problematics but should also give some background on genome evolution, polyploidy including sub-genome dominance, paloegenomics that are topics largely developed in the results. Paragraphs 1&2 should be merged and reduced (especially Line 60-70). Line 72: meaning of “barely soluble soil phosphates” unclear; Line 74: same remark as in abstract; Line 77: specify if high P use is the real focus of your work or low P adaptation and high P acquisition? Line 81-82: the genome itself is not sufficient to reveal “the detailed mechanisms of high P use” efficiency.
The results section is too long and may be reorganised in order to condense it on the most significant results from this study.
On genome sequencing: it would be good to give the name of the sequenced accession here. Also, it is surprising that no information about the availability of the sequencing data is given in the text (only in the reporting summary). The sequencing data should be made publicly available for the review and then upon publication. The genome from this study should be discussed relative to Hufnagel et al. BioRXiv 2019.
On assembly and annotation: Can the authors explain the limitation of their assembly: the PacBio technology usually provides long reads that allow capturing almost all contigs into pseudomolecules. The 25 chromosomes of their assembly is captured by 1771 contigs. Can the authors give some information about why 3482 contigs were not captured (HiC did not worked well? Genetic maps were missing? Other?). The annotation method is classical but the choice of legume proteomes included in the homology search was surprising: why not include Medicago truncatula, a reference sequence obtained from Sanger sequencing and for which the gene annotation has been more polished than other more recent sequences. The number of predicted genes is high though, relative to other recently produced genomes. Are some of these 59,014 genes TE-related genes? TE annotation is a bit weak for characterizing the types of TEs; tools that were used probably did not allow to discover old TE and total TE content is probably underestimated (see Hufnagel et al. BioRXiv). However, this is not impacting the information of density of TE that is used in the following analyses in the paper. The genome from this study should be discussed relative to Hufnagel et al. BioRXiv 2019.
The reconstruction of WL ancestor is using only Phaseolus vulgaris as reference. This probably induced a bias in the identification of genomic blocks that could have been limited by using several references (including one galegoid genome). The paragraph should be reduced and explanations on the method

should be transferred to the method section. The reconstruction provided here should be compared to what was observed by Hufnagel et al. 2019. Line 150 and later, the term "Ks loci" is not usually used. Use synonymous site instead? Line 153: what means "we determined orthologous instead of syntenic genes?". Line 201-209 is not clear.
The sub-genome dominance paragraph is original, but should be reduced and some parts rephrased (for example Line 236-240, Line 261-266, Line 269-273, Lines 290-295). Line 261: are these RNA sequencing present in a database? They should be made publicly available. Line 270-273: what are the numbers of pairwise paralogs: do they correspond to genes only present on two subgenomes? Line 285: the meaning of horse race is not precise. Why not simply indicate ~1?
In the paragraph on paralog gene retention, the fact that some GO families are more retained in white lupin as compared to A. thaliana and L. angustifolius is interesting (Line 315-379) but overall the paragraph should be condensed. What life history trait differing in white lupin and L angustifolius could explain this differential adaptation? Lines 345-348 are redundant. Line 356: the notion of transposition duplicated genes is obscure. Different scenarios could have led to this results without the involvement of TE.
In the paragraph (lines 380-519) and throughout the manuscript, the adaptation of white lupin to low phosphorus is highlighted. However, the analysis and insights on this topic seem limited in the present form in the manuscript. The paragraph is confused and difficult to follow. The RNAseq data described are interesting considering the specific mode of P uptake of white lupin. But the choice of focusing only on PUE genes identified in Arabidopsis is part of the reason why no really new information is presented. It is very surprising that other work on cluster root gene expression studies are not cited or discussed (O'Rourke et al. 2013, Secco et al. 2014). The authors should give an overview and give a coherent view of their own expression data results, before comparing to Arabidopsis thaliana PUE genes and more importantly, to previous data in white lupin. The choice of the candidate genes that are discussed in this paragraph is not clear: were they chosen based on the authors results? or based only on the literature? Also, this paragraph gives much redundant information as compare to the previous paragraph on gene retention. All mentions to gene expansion should be purged here and transferred, if needed, to the preceding paragraph. Furthermore, it is obvious that as compared to Arabidopsis, the recent triploid white lupin should present more paralogs. The RNAseq sequencing data should be made publicly available.
Table 1: It is strange that N50 lengths are smaller than N90 ones. Should be the contrary.
Figure 1: the choice of colors should be revised because it is difficult to differentiate lalb & lalb curve from lalb&lang curve, as well as gmax&gmax from lalb &gmax.
Figure 2: fig2c is difficult to read.
Figure 3: horse race is not a generic wording. Why not simply use 1?
Figure 4 seems not very meaningful. Why highlight these genes and not others showing higher fold ratios? Could be replaced by suppl. Figure 8.
Figure 6 does not seem to be stemming from the results and could be either deleted or redrawn for highlighting the specific results from this study.
Supplementary Figure 2: It is very strange that high gene density regions also correspond to high TE density regions, as shown on this figure (2) and (3). How can the authors explain that?

Reviewer #2 (Remarks to the Author):

This manuscript describes the sequencing and annotation of white lupin. The assembly sounds like it is of high quality - though I was not able to access it to try to assess this myself. Interesting aspects of the analysis include inference of an ancestral genistoid genome structure (prior to the whole-genome triplication that occurred early in the Genisteae, prior to divergence of Lupinus), and an initial analysis of evolution of genes involved in phosphorus metabolism. The manuscript is reasonably well written, though has some grammatical errors. The main defect I see in the paper currently is that the data are not available for review (neither the genome assembly nor the gene annotations: GFF, CDS, protein, mRNA).

Abstract:
"has high P use" - shouldn't this be something like "is able to thrive on soils with limited P" or "efficient P use in low-P soils"

Introduction
"existed in compounds, which makes" --> "exist in compounds that make"

"is an effective strategy" --> "is an attractive alternative strategy" (the strategy will only be effective if it is implemented well; that is: it is not a viable strategy currently.)

"Evolution led crops to develop" - this sounds teleological. Also, it's not really "crops," but "plant species" in general.

"Some research have" --> "Some research has"

"sequencing plant of white lupin" - do you mean "the genome of white lupin"?

Results:
"Leguminosae species" - this could be shortened (in several places) to "legume species." Formally, "Leguminosae" is a noun, whereas "legume" has qualities of a noun ("a legume") and an adjective (essentially, as a shortening of "leguminous"). Gramatically, "species of Leguminosae" and "legume species" are both correct, and "Leguminosae species" is not.

"syntenic genes pairs" --> "syntenic gene pairs"

"between two groups of Leguminosae species" - it would be good to use accepted taxonomic names for these clades. See LPWG (2017; https://doi.org/10.12705/661.3) or Ren et al., (2019; https://doi.org/10.1111/nph.15770)

"the left 36.82% of the white lupin" --> "the leftover 36.82% of the white lupin"

Section on genomic blocks and ancestral chromosomal states: it would be good to cite two new papers that report similar reconstructions (and I believe come to similar conclusions - well, at least Ren et al. also infers of a pre-WGT ancestral state for the Genisteae group (containing lupin) of 9 chromosomes): Ren et al., 2019 (https://doi.org/10.1111/nph.15770) and Kreplak et al., 2019 (https://doi.org/10.1038/s41588-019-0480-1).

"allo-polyploidy" - "allopolyploidy" (no hyphen) is more common in the literature, I believe.

"response to P deficient" --> "response to P deficiency"

Data availability: I am not able to see accession PRJNA558877 at GenBank. That looks like a bioproject identifier rather than an assembly accession. Further, deposition of annotations (gene sequences and gene models) are typically done separately from the assembly. Sometimes, annotations are made available via a different repository - for example, DataDryad, Figshare, Zenodo, GigaScience, or LegumeInfo. For now, I consider the assembly and annotations to be UNAVAILABLE. I don't feel comforatable recommending acceptance of the manuscript until these data sets are available.

Reviewer #3 (Remarks to the Author):

The paper “Co-evolution of multiple pathways to low phosphorus adaptation in white lupin” by Xu et al. describes the sequencing and analysis of the white lupin genome. This work is novel and of considerable interest, considering that white lupin serves as a model for plant adaptations to nutrient deficiency.
Illumina Genomic Survey and Long-read sequencing (Pac Bio) were used to sequence the lupin genome; HiC sequencing aided in mapping the sequences onto the corresponding chromosomes. Bioinformatics combined with RNA-seq aided in genome annotation and expression analysis.
Authors used comparative genomics to convincingly shed light on the unusual evolution of white lupin, revealing two whole genome duplication (WGD) events and expansion of phosphate-starvation related genes.

Overall, I thought the paper was of considerable interest; the genome sequencing and analysis sections appear well done and are easy to follow, though the language will benefit from some revisions.

My main concern with this paper are the conclusions on cluster root development.
Autors show that homologues of auxin precurser (IBA) transporters are expanded in white lupin. Two of these genes (ABCG36s and ABCG37s) showed increased expression during maturation of cluster roots; auxin concentration also fluctuated during cluster root development. This is a potentially very interesting correlation, considering that auxin is known to contribute to cluster root development.
To show causation, authors grew lupin without phosphate, but with addition of EDTA; EDTA has been shown previously to inhibit ABC transporters, and indeed, cluster roots formation was reduced. My problem with this approach is that EDTA chelates metals, and thus can inhibit many enzymes, not specifically the two ABC transporters of interest. While this is an interesting correlation, I would not agree that causation has been established, and advise a more cautious interpretation of the data.

The discussion doesn’t do the huge amount of work and findings justice; the first part is basically a summary of the background, followed by somewhat random statements that don’t tell a compelling story. I suggest to carefully revise the discussion in order to highlight and interpret the main results, and put them into perspective with the current literature.

Figures were for the most part informative and helped to illustrate the results, though I have some suggestions and concerns outlined in minor points.

Minor points (For additional minor points, please see attached pdf)

I suggest some language revisions throughout

Fig. 1 b: why is the Ks higher and frequency of syntenic gene pairs lower in L. albus vs. L. albus (red line) than in L. albus vs. L. angustifolius (magenta)?

Fig. 3 b, c,d: don’t understand these figures; what exactly are the x and y axis? What does horse-race mean for the first unit of x-axis? Also, would it be possible to combine 1 b,c,d into 1 graph?

Fig. 5 f should come before Fig. 5c as it helps to understand the x-axis of several panels in this figure

Fig. 5d not needed, and may be an over-simplification

Fig. 6, panel 2, perhaps a dashed arrow to indicate a correlation

Methods
Needs some editing, spaces are often missing.

See pdf for more minor points

Responses to reviewers' comments point by point

Comments from Reviewer #1:

Question 1: Xu et al. "Co-evolution of multiple pathways to low phosphorus adaptation in white lupin" reports a version of the white lupin genome of cv Amiga. White lupin is of particular interest because it is a protein-rich crop that has developed a peculiar adaptation to soil phosphorus deficiency. The authors present results on the structure, evolution and expression of the white lupin genome, with a focus on P acquisition- and metabolism-related genes. The manuscript brings interesting results for example on sub-genome dominance and link with TE density in white lupin, or on paralogs' retention, but overall, the text is too long and should be deeply re-written in order to better convey the results.

Answer 1: Thank you very much for your comments! Yes, we have largely reduced and re-written the manuscript as advised to better convey our results. We provided an editing-tracked version of the revised manuscript for easily tracing of these revisions (IN BLUE). Detail revisions have also been referred in below responses.

Q2: The wording is often unclear, redundant or not precise enough. The terms P-use or high-P-use are in most cases inappropriate in the paper and should be replaced by P use efficiency, or P acquisition or uptake, or low-P adaptation.

A2: We have revised all the terms of P-use or high-P-use throughout the manuscript as advised. We also asked help from native English editing company to revised wording and other English writing problems (see attached editing certificate).

Q3: The title does not convey the general content of the paper.

A3: We have changed the title to "The genome evolution and low phosphorus adaptation in white lupin (*Lupinus albus*)".

Q4: The abstract should be revised and checked. White lupin is not the only crop that develops cluster roots (see for example Plant and Soil (2005) 274:101–125). Lines 33-35 are not clear: the notion of sub-genome dominance is not straightforward and "the biased distribution of TE" is not clear enough. Express in a clearer way that on average, the genic expression of the different subgenomes vary according to the TE density of these subgenomes. Lines 35-38 seems contradictory. Have P-related genes been expanded more through the triplication event, or through tandem duplication? The notion of transposed duplication is unclear and is not supported by the results. Avoid using etc. at Line 39 and at other places in the text but rather be more specific or give a reference. Line 44, what is the enigma of genome evolution? More simply write "gives insights into genome evolution".

A4: Yes, we have revised this abstract as suggested to make them clear, and please it in detail. The P-related genes were over-retained through fractionation of paralogous genes after WGT in white lupin, and were also expanded through tandem and other ways of duplications. There are more PUE genes retained through fractionation after WGT.

Q5: The introduction should be re-written to be more specific and accurate. It focuses mainly on the phosphorus problematics but should also give some background on genome evolution, polyploidy including sub-genome dominance, paloegenomics that are topics largely developed in the results. Paragraphs 1&2 should be merged and reduced (especially Line 60-70). Line 72: meaning of "barely soluble soil phosphates" unclear; Line 74: same remark as in abstract; Line 77: specify if high P use is the real focus of your work or low P adaptation and high P acquisition? Line 81-82: the genome itself is not sufficient to reveal "the detailed mechanisms of high P use" efficiency.

A5: We have re-written the introduction section as suggested (Line 45, 46, 48-49, 49-50), and paragraphs 1&2 have be merged. We have added a paragraph on genome evolution, polyploidy including sub-genome dominance, paloegenomics as advised.

Q6: The results section is too long and may be reorganised in order to condense it on the most significant results from this study.

A6: We have largely condensed the result part as suggested.

Q7: On genome sequencing: it would be good to give the name of the sequenced accession here. Also, it is surprising that no information about the availability of the sequencing data is given in the text (only in the reporting summary). The sequencing data should be made publicly available for the review and then upon publication. The genome from this study should be discussed relative to Hufnagel et al. BioRXiv 2019.

A7: We have added the name of the sequenced white lupin in the place as suggested. We also provided the NCBI accession code and a website link in the revised manuscript to make sure that all the datasets referred in this study are freely available. We have also compared our genome with Hufnagel et al. BioRXiv 2019, and we found they have a good chromosomal synteny relationship, except for some small scale segment inversions (see below figure of two genomes comparison generated by NUCMER alignment). The two assembly have different orders and orientations of chromosomes, we ordered white lupin chromosomes 1-25 based on the length of these chromosomes.

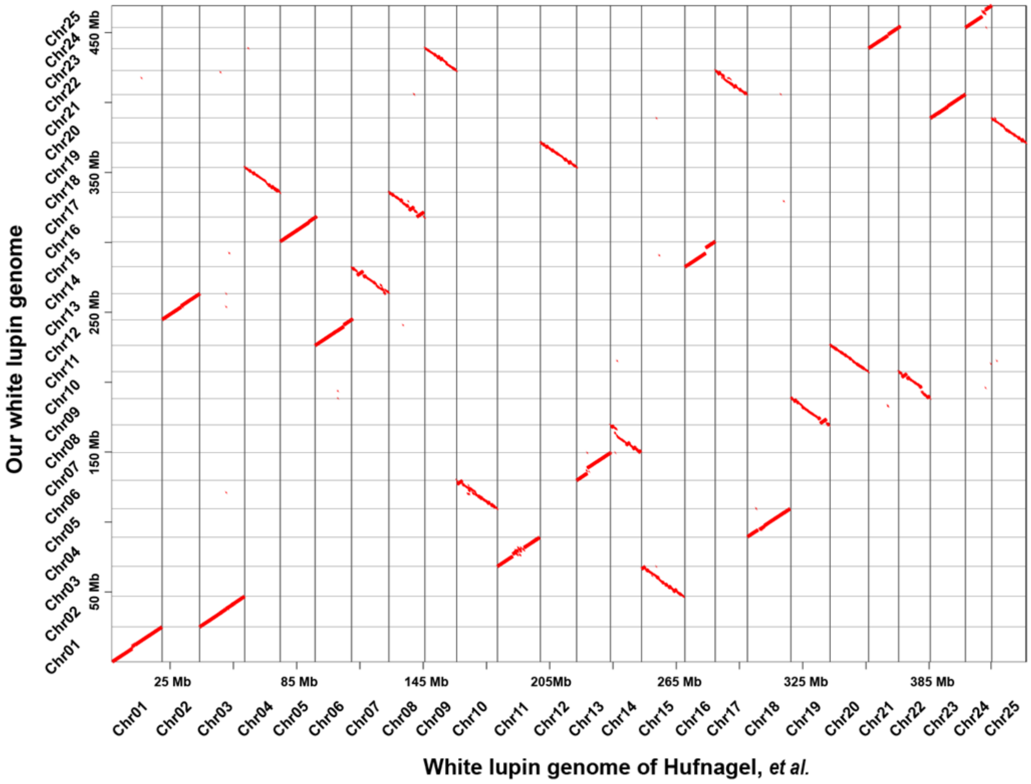

Q8: On assembly and annotation: Can the authors explain the limitation of their assembly: the PacBio technology usually provides long reads that allow capturing almost all contigs into pseudomolecules. The 25 chromosomes of their assembly is captured by 1771 contigs. Can the authors give some information about why 3482 contigs were not captured (HiC did not worked well? Genetic maps were missing? Other?). The annotation method is classical but the choice of legume proteomes included in the homology search was surprising: why not include Medicago truncatula, a reference sequence obtained from Sanger sequencing and for which the gene annotation has been more polished than other more recent sequences. The number of predicted genes is high though, relative to other recently produced genomes. Are some of these 59,014 genes TE-related genes? TE annotation is a bit weak for characterizing the types of TEs; tools that were used probably did not allow to discover old TE and total TE content is probably underestimated (see Hufnagel et al. BioRXiv). However, this is not impacting the information of density of TE that is used in the following analyses in the paper. The genome from this study should be discussed relative to Hufnagel et al. BioRXiv 2019.

A8: Thanks for the comments and advises. The ratio of anchored contigs by HiC data is comparable with other reported genomes, such as studies in Musa and *B. rapa* genomes (Wang, Z., *et al.*, 2019, Nature Plants; Zhang, L., *et al.*, 2018, Horticulture Research). Using the PacBio sequencing reads, we have assembled 7,005 contigs. In the updated version, we removed contigs with low sequence complexity, contaminated contigs, and heterozygous sequences. Finally obtained 3,171 contigs. Among which, 1,616 contigs have been assembled into 25 pseudomolecules. The 25 pseudomolecules covered 84.87% of the total length of assembled contigs. These left contigs have not enough HiC links to assign them into pseudo-chromosomes. Additionally, the rate of mapped HiC contacts to all HiC reads is 39.73%, which indicates the high quality of our HiC data comparing to other genome assembly (normally distributed around 20%-40%), thus our HiC dataset is OK.

We have re-annotated the white lupin genome with more legume species, such as *Medicago truncatula* as suggested, as well as *Arachis duranensis*, *vigan angularis*, and *lotus japonicas*, together with ~200 Gb mRNA sequencing data from 30 samples. Finally, we predicted 48,719 genes in the updated annotation. TE related genes were masked by tools RepeatMasker and RepeatProteinMasker, thus our gene set doesn't include TE related genes.

We followed the traditional and widely used methods (RepeatMasker pipeline) to annotate the TEs in white lupin genome. However, in the revised manuscript, we further applied the TE annotation methods (RepeatExplorer and Dante) used by Hufnagel et al. BioRXiv 2019, and merged the two TE datasets to do the analysis. Yes, with the new TE annotation, we re-do the analysis and found that it is not impacting the previous results on density of TEs (see Figure 3). We also compared TEs annotated by RepeatMasker to that annotated by RepeatExplorer (Hufnagel et al. BioRXiv 2019), the results show that RepeatMasker annotated 261.20 Mb sequences as repeats, while RepeatExplorer annotated 35.18 Mb repeat sequences. The two combined annotations has 262.71 Mb repeats, and RepeatExplorer annotation contributed only 1.51 Mb repeats to RepeatMasker annotation.

Q9: The reconstruction of WL ancestor is using only Phaseolus vulgaris as reference. This probably induced a bias in the identification of genomic blocks that could have

been limited by using several references (including one galegoid genome). The paragraph should be reduced and explanations on the method should be transferred to the method section. The reconstruction provided here should be compared to what was observed by Hufnagel et al. 2019. Line 150 and later, the term “Ks loci” is not usually used. Use synonymous site instead? Line 153: what means “we determined orthologous instead of syntenic genes?”. Line 201-209 is not clear.

A9: Thanks for the comments. We selected *Phaseolus vulgaris* as a reference, for its relatively distant divergence to white lupin in Leguminosae family. Here, we have added *M. truncatula*, one galegoid genome as advised and also *A. ipaensis*, a dalbergioid genome in the revised manuscript. They produce similar results, which doesn’t change the association relationships of genomic blocks determined in previous genome analysis of white lupin.

We have largely reduced corresponding paragraph as suggested, and we compared our reconstruction with Hufnagel et al. 2019, the overall genomic karyotype evolution is consistent between the two manuscripts. However, we further reconstructed the diploid ancestor of white lupin between WGT event, and the diploid ancestor show similar genomic structure to the deduced ancestor of Genistoid clade (lupin) a previous study (Ren et al., 2019, https://doi.org/10.1111/nph.15770), which also has nine chromosomes.

We have revised “K_s loci” to synonymous sites as suggested.

Because the Brassicaceae species *Arabidopsis thaliana* is distantly divergent from legume, genome synteny between legume species and *A. thaliana* are largely lost during evolution and became undetectable, thus there are limited syntenic genes can be found between them. Therefore, we determined orthologous genes between legume species and *A. thaliana* only based on sequence homology, other than based on both chromosomal synteny and sequence homology. We have removed according contents in the results section, and revised related sentences in the methods section to avoid confusion.

Q10: The sub-genome dominance paragraph is original, but should be reduced and some parts rephrased (for example Line 236-240, Line 261-266, Line 269-273, Lines

290-295). Line 261: are these RNA sequencing present in a database? They should be made publicly available. Line 270-273: what are the numbers of pairwise paralogs: do they correspond to genes only present on two subgenomes? Line 285: the meaning of horse race is not precise. Why not simply indicate ~1?

A10: We have revised these parts as suggested. These RNA sequencing reads were freely available through link: http://brassicadb.org/brad/pub/genomes/Lalbus/, and we have provided this information in the revised manuscript. Yes, pairwise paralogs mean paralogous genes from either two of the three sub-genomes (LF *vs.* MF1, LF *vs.* MF2, and MF1 *vs.* MF2), both of the two genes in each paralogous pair show synteny to the same gene in diploid species as *P. vulgaris*. We have replaced “horse race” with “one fold” as suggested.

Q11: In the paragraph on paralog gene retention, the fact that some GO families are more retained in white lupin as compared to *A. thaliana* and *L. angustifolius* is interesting (Line 315-379) but overall the paragraph should be condensed. What life history trait differing in white lupin and L angustifolius could explain this differential adaptation? Lines 345-348 are redundant. Line 356: the notion of transposition duplicated genes is obscure. Different scenarios could have led to this results without the involvement of TE.

A11: We condensed the paragraph as suggested. Redundant sentences have been removed in lines 345-348. We have revised line 356 to make it clear (Line 237-238). We think that genes triplicated through different ways were submitted to different selection pressure in white lupin and *L. angustifolius*, which then resulted in the current differences in GO families. Yes, we agree that transpose duplicated genes don’t mean that they were duplicated through the involvement of TEs. We used “transpose” to describe their dispersed distribution—these genes do not keep the ancestral location comparing to close related genomes. To make it clear, we revised and used “disperse or other ways of duplicated genes” to include cases that can’t be simply grouped into genes duplicated through transposition.

Q12: In the paragraph (lines 380-519) and throughout the manuscript, the adaptation of white lupin to low phosphorus is highlighted. However, the analysis and insights on this topic seem limited in the present form in the manuscript. The paragraph is confused and difficult to follow. The RNAseq data described are interesting considering the specific mode of P uptake of white lupin. But the choice of focusing only on PUE genes identified in Arabidopsis is part of the reason why no really new information is presented. It is very surprising that other work on cluster root gene expression studies are not cited or discussed (O'Rourke et al. 2013, Secco et al. 2014). The authors should give an overview and give a coherent view of their own expression data results, before comparing to Arabidopsis thaliana PUE genes and more importantly, to previous data in white lupin.

A12: As advised, in the revised manuscript, we have first gave a brief review of our mRNA-seq data and found that GO terms associated with lipid metabolic process, carbohydrate metabolic process, transport and cellular response to phosphate starvation were significantly enriched in low-P induced gene clusters (**Supplementary Figures 10 and 11**), suggesting a classical phosphate starvation response in white lupin. Secondly, we compared our expression data results with the previous published P deficiency transcriptomic studies in white lupin (O'Rourke et al. 2013, Secco et al. 2014). This result was depicted in the **Supplementary Table 26**. Finally, we found that some genes related to auxin modulation, CO_2 fixation and acid phosphatase were commonly differentially expressed in all three studies, emphasizing the importance of these processes for high PUE in white lupin, and two genes *ABCG36* and *ABCG37* were found to be important to the high PUE of white lupin. We have re-written and condensed this section.

Q13: The choice of the candidate genes that are discussed in this paragraph is not clear: were they chosen based on the authors results? or based only on the literature? Also, this paragraph gives much redundant information as compare to the previous paragraph on gene retention. All mentions to gene expansion should be purged here and transferred, if needed, to the preceding paragraph. Furthermore, it is obvious that as compared to Arabidopsis, the recent triploid white lupin should present more paralogs. The RNAseq sequencing data should be made publicly available.

A13: We chose these candidate genes not only based on our mRNA-seq results, but also on the literature, as well as on GO enrichment of genes specially duplicated in white lupin. We have removed redundant contents and re-write corresponding paragraph as suggested. Yes, the recent triploid white lupin should present more paralogs across different gene families. What we focused is that the biased retention of gene families through extensive fractionation of paralogous genes following WGT, and their difference between white lupin and *L. angustifolius*. The RNA-seq datasets are freely available.

Q14: Table 1: It is strange that N50 lengths are smaller than N90 ones. Should be the contrary.

Figure 1: the choice of colors should be revised because it is difficult to differentiate lalb & lalb curve from lalb&lang curve, as well as gmax&gmax from lalb &gmax.

Figure 2: fig2c is difficult to read.

Figure 3: horse race is not a generic wording. Why not simply use 1?

Figure 4 seems not very meaningful. Why highlight these genes and not others showing higher fold ratios? Could be replaced by suppl. Figure 8.

Figure 6 does not seem to be stemming from the results and could be either deleted or redrawn for highlighting the specific results from this study.

Supplementary Figure 2: It is very strange that high gene density regions also correspond to high TE density regions, as shown on this figure (2) and (3). How can the authors explain that?

A14: Many thanks for your good comments. For table 1, we have moved the N90 statistics below that of N50 in the revised manuscript. We revised or replaced Figure 1, 2, 3, 4, and 6 as suggested. In respect to Supplementary Figure 2, we are sorry that we used a reversed order of colors to indicate the densty of TEs, and we have corrected the mistake in the revised version.

Comments from Reviewer #2:

Q1: This manuscript describes the sequencing and annotation of white lupin. The assembly sounds like it is of high quality - though I was not able to access it to try to assess this myself. Interesting aspects of the analysis include inference of an ancestral genistoid genome structure (prior to the whole-genome triplication that occurred early in the Genisteae, prior to divergence of Lupinus), and an initial analysis of evolution of genes involved in phosphorus metabolism. The manuscript is reasonably well written, though has some grammatical errors. The main defect I see in the paper currently is that the data are not available for review (neither the genome assembly nor the gene annotations: GFF, CDS, protein, mRNA).

A1: Thanks you very much for your positive comments! We are sorry that the datasets were not accessible in previous version. In the revised version, we uploaded the datasets used in this analysis in two places as NCBI database and http://brassicadb.org/brad/pub/genomes/Lalbus/ to make sure all datasets are freely available.

Q2: Abstract: "has high P use" - shouldn't this be something like "is able to thrive on soils with limited P" or "efficient P use in low-P soils"

A2: Yes, we have revised "has high P use" into “has high P-use-efficiency (PUE) in low-P soils” as advised (IN BLUE).

Q3: Introduction

"existed in compounds, which makes" --> "exist in compounds that make"

"is an effective strategy" --> "is an attractive alternative strategy" (the strategy will only be effective if it is implemented well; that is: it is not a viable strategy currently.)

"Evolution led crops to develop" - this sounds teleological. Also, it's not really "crops," but "plant species" in general.

"Some research have" --> "Some research has"

"sequencing plant of white lupin" - do you mean "the genome of white lupin"?

A3: We have revised these words and sentences as suggested in the revised manuscript.

Q4: Results:

"Leguminosae species" - this could be shortened (in several places) to "legume species." Formally, "Leguminosae" is a noun, whereas "legume" has qualities of a noun ("a legume") and an adjective (essentially, as a shortening of "leguminous"). Gramatically, "species of Leguminosae" and "legume species" are both correct, and "Leguminosae species" is not.

"syntenic genes pairs" --> "syntenic gene pairs"

"between two groups of Leguminosae species" - it would be good to use accepted taxonomic names for these clades. See LPWG (2017; https://doi.org/10.12705/661.3) or Ren et al., (2019; https://doi.org/10.1111/nph.15770)

"the left 36.82% of the white lupin" --> "the leftover 36.82% of the white lupin"

Section on genomic blocks and ancestral chromosomal states: it would be good to cite two new papers that report similar reconstructions (and I believe come to similar conclusions - well, at least Ren et al. also infers of a pre-WGT ancestral state for the Genisteae group (containing lupin) of 9 chromosomes): Ren et al., 2019 (https://doi.org/10.1111/nph.15770) and Kreplak et al., 2019 (https://doi.org/10.1038/s41588-019-0480-1).

"allo-polyploidy" - "allopolyploidy" (no hyphen) is more common in the literature, I believe.

"response to P deficient" --> "response to P deficiency"

A4: Yes, we have revised according parts as suggested (legume species; syntenic gene pairs; leftover; allopolyploidy; deficiency). We also used accepted taxonomic names (Genistoid, Galegoid, Milletioid and Dalbergioid) for different legume clades in Fig. 1a and the maintext. Yes, we compared and added the two reference papers as suggested.

Q5: Data availability: I am not able to see accession PRJNA558877 at GenBank. That looks like a bioproject identifier rather than an assembly accession. Further, deposition of annotations (gene sequences and gene models) are typically done separately from the assembly. Sometimes, annotations are made available via a different repository - for example, DataDryad, Figshare, Zenodo, GigaScience, or LegumeInfo. For now, I consider the assembly and annotations to be UNAVAILABLE. I don't feel comfortable recommending acceptance of the manuscript until these data sets are available.

A5: Sorry for the inconvenience again, we have uploaded all datasets used in this work in both NCBI database and http://brassicadb.org/brad/pub/genomes/Lalbus/, and double checked to make sure that there are freely available this time.

Comments from Reviewer #3:

Q1: The paper "Co-evolution of multiple pathways to low phosphorus adaptation in white lupin" by Xu et al. describes the sequencing and analysis of the white lupin genome. This work is novel and of considerable interest, considering that white lupin serves as a model for plant adaptations to nutrient deficiency. Illumina Genomic Survey and Long-read sequencing (Pac Bio) were used to sequence the lupin genome; HiC sequencing aided in mapping the sequences onto the corresponding chromosomes. Bioinformatics combined with RNA-seq aided in genome annotation and expression analysis. Authors used comparative genomics to convincingly shed light on the unusual evolution of white lupin, revealing two whole genome duplication (WGD) events and expansion of phosphate-starvation related genes. Overall, I thought the paper was of considerable interest; the genome sequencing and analysis sections appear well done and are easy to follow, though the language will benefit from some revisions.

A1: Many thanks for your positive comments. Yes, we have revised our manuscript extensively (IN BLUE) and asked editing service from native English speaker through the LetPub's language editing company. We have attached the editing certificate with this revised submission.

Q2: My main concern with this paper are the conclusions on cluster root development. Authors show that homologues of auxin precurser (IBA) transporters are expanded in white lupin. Two of these genes (ABCG36s and ABCG37s) showed increased expression during maturation of cluster roots; auxin concentration also fluctuated during cluster root development. This is a potentially very interesting correlation, considering that auxin is known to contribute to cluster root development. To show causation, authors grew lupin without phosphate, but with addition of EDTA; EDTA has been shown previously to inhibit ABC transporters, and indeed, cluster roots formation was reduced. My problem with this approach is that EDTA chelates metals, and thus can inhibit many enzymes, not specifically the two ABC transporters of interest. While this is an interesting correlation, I would not agree that causation has been established, and advise a more cautious interpretation of the data.

A2: Yes, we agreed with the reviewer's opinion that EDTA treatment and the inhibition of two ABC transporters activity is an interesting correlation. We performed another pharmacological treatment with specific auxin polar transport inhibitor to the white lupin and a significant decline in cluster root number was observed under P deficient condition. We also tune down corresponding sentence to "auxin modulation may be important for the cluster root formation and high PUE of white lupin by involving two potential genes, ABCG36 or ABCG37, which belong to the G subgroup of the ATP-binding cassette transporter". Please see the revised **Fig. 5** for detail. We have re-written and condensed this section based on our new analyses.

Q3: The discussion doesn't do the huge amount of work and findings justice; the first part is basically a summary of the background, followed by somewhat random statements that don't tell a compelling story. I suggest to carefully revise the discussion in order to highlight and interpret the main results, and put them into perspective with the current literature.

A3: We have condensed and revised the discussion section as suggested.

Q4: Minor points (For additional minor points, please see attached pdf: MS and methods)

A4: Yes, we have revised these points as suggested.

Q5: I suggest some language revisions throughout.

A5: Yes, we have carefully revised the manuscript and asked editing service from LetPub's language editing company. See attached editing certificate.

Q6: Fig. 1 b: why is the K_s higher and frequency of syntenic gene pairs lower in *L. albus* vs. *L. albus* (red line) than in *L. albus* vs. *L. angustifolius* (magenta)?

A6: For these relatively recent polyploidization events, paralogous genes have insufficient time for divergence, and the scale of divergent variation is limited, thus

they have a narrow divergence ratio and the distribution peak of K_s values is sharp and higher. Wile for these older polyploidization events, paralogous genes have much more time for divergence, and the divergent variation of different paralogs is bigger, thus the K_s distribution peak is more flat and lower.

Q7: Fig. 3 b, c, d: don't understand these figures; what exactly are the x and y axis? What does horse-race mean for the first unit of x-axis? Also, would it be possible to combine 3 b, c, d into 1 graph?

A7: We have revised the figure legend to make it clear. *x*-axis denote the thresholds to determine a pair of paralogous gene that show dominant expression of one gene to the other one. *y*-axis denote the number of genes from one sub-genome that are dominantly expressed over their paralogs from another sun-genome. We can't combine 3 b, c, d to 1 graph for that there are different numbers of paralogous gene pairs that were compared among sub-genome pairs LF/MF1, LF/MF2, MF1/MF2.

Q8: Fig. 5 f should come before Fig. 5c as it helps to understand the x-axis of several panels in this figure. Fig. 5d not needed, and may be an over-simplification.

A8: Yes, we have re-organized Fig. 5 as suggested.

Q9: Fig. 6, panel 2, perhaps a dashed arrow to indicate a correlation.

A9: We have re-drawn Fig. 6 as suggested.

Q10: Methods: Needs some editing, spaces are often missing.

A10: We have revised the Methods section thoroughly.

(5) Comments from Reviewer #3 in the **attached-pdf MS**

Question#1: Line 52 “existed”.

Answer: “existed” has been replaced by “exist”.

Question#2: Line 52 “…in compound…”, please explain this.

Answer: “…in compound…” have been changed into “ These less mobile P exist in compounds that make it more difficult to be absorbed by crops. ”

Explain: the compound means “Ca-P, Fe-P, Al-P, organic P” (as described in the paper: Nguyen and Maeda. 2016. Journal of Water and Environment Technology. 14: 228–235), which have been mentioned in the above sentence “it is often present in unavailable forms, such as phytic acid, or calcium (Ca), iron (Fe), and aluminum (Al) phosphates[1,2].”.

Question#3: Line 59 “…high-P-use crops…”, please explain this.

Answer: The sentence “…high-P-use…” has been replaced by “The development of high P use efficiency (PUE) for crops is an attractive alternative strategy”. Explain: the PUE crops mean this crop that can survive in low-P soil and have the ability for high P acquisition and low-P adaptation.

Question#4: Line 60 “Low P availability in soil is one of the major limiting factors for crop growth and development”, this sentence belongs to the previous paragraph.

Answer: “Low P availability in soil is one of the major limiting factors for crop growth and development” has been replaced by “To cope with P-limiting environments,”.

Question#5: Line 61 “Evolution led crops…”, this sounds like evolution had a plan, which it doesn't; rephrase.

Answer: “Evolution led crops to develop flexible strategies to cope with P deficiency” has been replaced by “plants have evolved flexible strategies”.

Question#6: Line 69-70 “and the establishment of symbiotic associations with mycorrhizal fungi that aid P acquisition”, misleading, considering that white lupin does not form mycorrhizal symbiosis.

Answer: “and the establishment of symbiotic associations with mycorrhizal fungi that aid P acquisition” was deleted.

Question#7: Line 71 “Leguminosae”. Fabaceae (Leguminosae is outdated).

Answer: “Leguminosae” has been replaced by “Fabaceae”.

Question#8: Line 76 “have”.

Answer: “have” has been replaced by “has”.

Question#9: Line 96 “sequencing plant of white lupin has”.

Answer: “sequencing plant of white lupin has” has been replaced by “white lupin used for sequencing had”.

Question#10: Line 107 “an”.

Answer: “an” has been replaced by “a”.

Question#11: Line 115 “homologous searching”.

Answer: “homologous searching” has been replaced by “homology search”.

Question#12: Line 120 “homologous”, redundant; rephrase

Answer: “and predict homologous genes” has been replaced by “and thus predict genes”.

Question#13: Line 347-348 “whole genome duplication, tandem duplication, and transposition duplication”, redundant

Answer: the sentence “There are three main ways for genes to be expanded in a genome: whole genome duplication, tandem duplication, and transposition duplication.” was deleted.

Question#14: Line 348 “We further separated the three groups of genes…”, What 3 groups?

Answer: “three groups” represents three main types of gene expansion. To make it clear, we rephrased this sentence.

“We further separated the three groups of genes in the genome of white lupin” has been replaced by “We further identified the three types of expanded genes in the genome of white lupin”.

Question#15: Line 381 “…show strong response…”, is this a result or literature citation?

Answer: this is a result from our own study (Line 266-268), and please see Fig. 4 and the following descriptions in this paragraph.

Question#16: Line 383-384 “lupin involves the concerted evolution of multiple biological processes such as cluster root growth and phosphorus use.”, citation?

Answer: “lupin involves the concerted evolution of multiple biological processes such as cluster root growth and phosphorus use.” have been moved into discussion section and was deleted in this paragraph.

Question#17: Line 384 “We collected genes related to some processes”, vague

Answer: “We collected genes related to some processes” has been replaced by “We further examined the expression levels of putative orthologs of 514 *Arabidopsis* genes that function in low P adaptive pathways”.

Question#18: Line 386-387 “P homeostasis (e.g., glycolysis, lipid remodeling, and mitochondrial electron transport)”, what makes these P homeostasis?

Answer: It has been changed into “These genes function in pathways such as P acquisition (e.g., TCA cycle, non-photosynthetic CO_2 fixation, phosphate transport, and root development) and P use (e.g., glycolysis, lipid remodeling, and mitochondrial electron transport), etc.”. We deleted this sentence in the revised main text.

Question#19: Line 444-445 “ATPase activity of ABCG36 and ABCG37 is crucial for cluster root formation in white lupin”, is this the only possible conclusion?

Answer: Yes, we agreed with the reviewer’s opinion. We performed another pharmacological treatment with specific auxin polar transport inhibitor to the white lupin and a significant decline in cluster root number was also observed under P deficient condition. We thus changed our conclusion to “auxin modulation may be important for the cluster root formation by involving *ABCG36* and *ABCG37* genes in white lupin”. Please see the revised Fig. 5 for detail.

Question#20: Line 451 “…CO_2 fixation through the TCA cycle”, I don't think that TCA fixes CO_2? As far as I know, dark fixation occurs in the cytosol via PEPC?

Answer: yes, PEPC performs an important role in dark (non-photosynthetic) CO_2 fixation in the cytosol. The sentence “A substantial proportion of carbon used for organic acid synthesis in roots requires CO_2 fixation through the TCA cycle” has been replaced by “In non-photosynthetic stem and root tissues, a substantial proportion of carbon was fixed by phosphoenolpyruvate carboxylase (PEPC) and the derived

oxaloacetate was further converted into other organic acids through TCA cycle ”. We deleted this sentence in the revised main text.

Question#21: Line 464 “phosphoenolpyruvate carboxylase”.

Answer: “phosphoenolpyruvate carboxylase,” was deleted.

Question#22: Line 465 “activate its activity”, redundant

Answer: “activate its activity” has been replaced by “activate it”.

Question#23: Line 471 “W. Shane’s”.

Answer: “W. Shane’s” has been replaced “Shane’s”.

Question#24: Line 532-534 “The appropriate P level in white lupin is helpful for protein phosphorylation in modulating gene action, including P-related genes, and vice versa”, where does this come from?

Answer: We deleted this sentence “The appropriate P level in white lupin is helpful for protein phosphorylation in modulating gene action, including P-related genes, and vice versa”.

Question#25: Line 538 “…PEPC genes (Supplementary Figs. 9 and 10)”, what is this about?

Line 538-539 “…which is important for supplying energy for the growth of the shoot and root through fixation of carbon”, distinguish between photosynthesis and dark fixation.

Answer: The previous Line 538 and Line 538-539 have been changed into “carbon fixation capacity increases with high expression of PEPC genes (**Supplementary Table 24**), which is important for root growth in response to P deficiency”.

Question#26: Line 541 "…indispensable roles in cluster root formation", correlation yes, but causation?

Answer: "the genes that regulate IAA homeostasis in white lupin play indispensable roles in cluster root formation" has been replaced by "Our results show that *ABCG36*/*ABCG37* (**Fig. 5**), the potential genes that regulate auxin homeostasis in white lupin, may be involved in cluster root formation".

(6) Comments from Reviewer #3 in the **attached-pdf about methods**

Question#1: Line 14 “If no otherwise specified”

Answer: “If no otherwise specified” has been replaced by “If not otherwise specified”.

Question#2: Line 20 “The results estimated that…”, should be in result section.

Answer: the sentence “The results estimated that…” was deleted, and it was presented in the result section.

Question#3: Line 57 “libraryof”

Answer: “libraryof” has been replaced by “library of”.

Question#4: Line 58 “constructedusing”

Answer: “constructedusing” has been replaced by “constructed using”.

Question#5: Line 74 “*A. Arabidopsis*”

Answer: “*A. Arabidopsis*” has been replaced by “*A. thaliana*”.

Question#6: Line 77 “Meanwhile, we have generated”, separate section

Answer: Yes, we separated it as a new paragraph.

Question#7: Line 82 “stringtie”

Answer: “stringtie” has been replaced by “StringTie”.

Question#8: Line 138 “Recent triplication of white lupin genome”, not a Methods header.

Answer: “Recent triplication of white lupin genome” has been replaced by “Analysis of whole genome triplication”.

Question#9: Line 163 “Leguminosae”

Answer: “Leguminosae” has been replaced by “Fabaceae”.

Reviewers' comments:

Reviewer #1 (Remarks to the Author):

The revised manuscript by Xu et al. on "The genome evolution and low phosphorus adaptation in white lupin" has been substantially improved as compared to the previous version. However, there are still some paragraphs that would need further improvements. In particular the paragraphs on 'The adaptive pathways of white lupin in response to low availability' (line 245-318) and the discussion (line 321-371) are very difficult to follow. What are the main messages from your analyses? What is new as compared to what is known in the literature?

Specific comments:
Line 50: you cannot write that the white lupin genome is not know. You should include in your paper the reference to genome from Hufnagel et al. and add a sentence and supplementary figure about the comparison of your sequence with their sequence.
Line 53: be careful; low P adaptation means low adaptation to P and not adaptation to low P; write low-P
Line 60: expressed rather than expressing
Line 61: has been shown to play …
Line 64: contribute
Line 134 vs 137 : synteny or syntenic?
Lines 158-159-160-: remove brackets.
L210: replace with by which?
Line 221-22 not clear; reword.
Line 230: the number of PUE genes has increased …
Line 230: remove transposition; unless you show evidence that this is the case.
Line 247 > what do you mean by 'in at least one'? briefly describe which data allows you to analyse this. RNAseq? other? Also give information about how the differential expression has been tested (test used and the level of significance).
Line 261: you should present the RNAseq data before.
Line 267: why brackets? Correct the wording.
Line304-305: idem.
Line328-348: remove 'also'
Line 328-356: too general and not shown by your results (carbon can be translocated from shoot to root … carbon fixation capacity increases with high expression of PepC… GDPD is important for low P adaptation); what is new in your results as compared to the literature? How do your results contribute to a general understanding of P nutrition in white lupin? Previous knowledge and your data should be better integrated.
Suppl Table 3: Vigna instead of vigan;
Suppl Table 6: Vigna instead of vigra
Suppl Figure 13-19: it would be good to add letters showing the significantly different classes according to a t-test, above the bars.

Reviewer #2 (Remarks to the Author):

This revision corrects most of my comments relative to the first submission. I feel that this revision is much more solid. The analyses look appropriate and well conducted.

I do have several additional, generally minor suggestions:

line 21 (abstract): "White lupin is the legume crop that" --> "White lupin is a legume crop that" -- "a" instead of "the" because there are other lupin species (L. mutabilis at least) that are used as crops

and have cluster roots and PUE.

lines 26-28 (abstract): "and revealed the sub-genome dominance phenomenon, as well as the genic expression of the different sub-genomes vary in relation to their TE density." This sentence is not grammatical. It would be better as: "and find sub-genome dominance, with genic expression of the different sub-genomes varying in relation to their TE density."

line 30: "four main pathways on high PUE of white lupin" --> "four main pathways involved in the high PUE of white lupin"

lines 63-64: "lupin will contributes" --> "lupin will contribute"

line 102: "were uploaded to the NCBI database" -- would be good to add the project number (PRJNA592024) or accession here:

line 118: "divergent ~39.97 million years" - does this refer to the divergence between Cicer and Phaseolus (I think so).

line 120: "divergent ~40.75 million years" - does this refer to the divergence between the two Arachis species? If so, this is incorrect. These species had a common ancestor around 2 million years ago (Bertioli et al., 2016)

line 121: "Two polyploidization events were found in these legume species" - this is too simple. It neglects to mention the important WGD that affects ALL papilionoid legumes, estimated to have occurred around 55 Mya and other, independent WGDs affecting other legumes (Cannon, McKain et al., 2015; Stai, Yadav et al., 2019). Another, recent WGD is seen in tetraploid Arachis. Other WGDs are seen in e.g. Mimosa and other lineages. It would be good to correct this in Figure 1a as well (adding a star at the base of the Papilionoid clade).

line 156: "white lupine" (extra "e")

Reviewer #3 (Remarks to the Author):

The manuscript "The genome evolution and low phosphorus adaptation in white lupin (Lupinus albus)" by Xu et al. has been carefully revised and my main concerns have been addressed.

I still believe that the language would benefit from some revisions, and I made many small suggestions directly in the pdf.

Minor points (in addition to the pdf):

Abstract
I suggest to put a little work in perfecting the abstract. The language is not incorrect, but considering the importance of the abstract, I think there is room for improvement.

Results
Page 9, lines 225-229
"Genes annotated as "glycolysis", "tricarboxylic acid cycle", "lipid biosynthetic process", "ATPase activity" and different types of phosphate-related functions increased in L. albus compared to L. angustifolius (Supplementary Table 14). This indicates that the function pathways of these genes evolved in L. albus after it diverged from L. angustifolius."

It is incorrect to state that these pathways evolved after divergence, as these are ancient pathways. How about: “This indicates that over-retaining of genes in these pathways occured in L. albus after it diverged from L. angustifolius”

Discussion

The structure of the discussion needs some work. The discussion states “four main biological pathways…” It then counts 3 pathways (not very clearly), and lacks the fourth.

Page 13, lines 337-341

“Our results suggest that carbon fixation capacity increases with high expression of PEPC genes (Supplementary Table 24), which is important for root growth in response to P deficiency. Secondly, under the support of carbon fixation, cluster root formation can be promoted.”

Is dark fixation really mainly important for root growth in response to low P? I always assumed its main purpose was to generate organic acids for excretion.

Materials and Methods

Phosphoproteomic analysis is described in the Materials and Methods, but I can’t find any mentioning in the results.

Point by point responses to reviewers' comments

Comments from Reviewer #1:

Question #1: The revised manuscript by Xu et al. on "The genome evolution and low phosphorus adaptation in white lupin" has been substantially improved as compared to the previous version. However, there are still some paragraphs that would need further improvements. In particular the paragraphs on 'The adaptive pathways of white lupin in response to low availability' (**line 245-318**) and the discussion (**line 321-371**) are very difficult to follow. What are the main messages from your analyses? What is new as compared to what is known in the literature?

Answer 1: Many thanks for your comments. Yes, we have extensively revised the paragraph of 'The adaptive pathways of white lupin in response to low availability' and the discussion and others section incorporating all suggestions (in blue).

Q2: Line 50: you cannot write that the white lupin genome is not know. You should include in your paper the reference to genome from Hufnagel et al. and add a sentence and supplementary figure about the comparison of your sequence with their sequence.

A2: Thanks for your comments, we revised the sentences. We highly valued the work of Hufnagel *et al.*, and would like to cite their preprint manuscript. However, the information of preprint version of Hufnagel *et al.*'s manuscript shows that their manuscript has been under review in Nature Communications, just as ours. We don't know if it is OK to cite their manuscript when both manuscript are under parallel review process in Nature Communications. We will discuss it with the editor to find a proper way to cite Hufnagel *et al.*'s manuscript and add the contents as suggested later. Thank you again very much.

Q3: Line 53: be careful; low P adaptation means low adaptation to P and not adaptation to low P; write low-P

A3: "low P adaptation**"** has been replaced by "low-P adaptation".

Q4: Line 60: expressed rather than expressing

A4: "expressing**"** has been replaced by "expressed".

Q5: Line 61: has been shown to play …

A5: "have also played" has been replaced by "has been shown to play".

Q6: Line 64: contribute

A6: "contributes" has been replaced by "contribute".

Q7: Line 134 vs 137 : synteny or syntenic?

A7: We revised synteny to syntenic.

Q8: Lines 158-159-160-: remove brackets.

A8: brackets have been deleted as suggested.

Q9: L 210: replace with by which?

A9: We replaced "with" by "which".

Q10: Line 221-22 not clear; reword.

A10: We have revised the sentence.

Q11: Line 230: the number of PUE genes has increased …

A11: "PUE genes have increased..." has been replaced by "the number of PUE genes has increased...".

Q12: Line 230: remove transposition; unless you show evidence that this is the case.

A12: We have removed transposition, and used dispersed to indicate other ways of duplication that cannot be grouped into WGT or tandem duplications.

Q13: Line 247 > what do you mean by 'in at least one'? briefly describe which data allows you to analyse this. RNAseq? other? Also give information about how the differential expression has been tested (test used and the level of significance).

A13: Thanks for your suggestions. We have briefly described our RNA-seq data and given the information about how the differential expression has been tested, detail analysis is provided in the Methods section.

Q14: Line 261: you should present the RNAseq data before.

A14: We have reordered this part to present results following: 1) the overall findings

of mRNA-seq analysis; 2) comparing the results to *Arabidopsis* and previous reports in white lupin; 3) new findings in this study on duplicated genes and their up-regulated or specific expression pattern related to low-P adaptation in white lupin.

Q15: Line 267: why brackets? Correct the wording.

A15: The brackets have been removed as suggested.

Q16: Line 304-305: idem.

A16: The brackets have been removed as suggested.

Q17: Line 328-348: remove ‘also’

A17: “also” were removed.

Q18: Line 328-356: too general and not shown by your results (carbon can be translocated from shoot to root … carbon fixation capacity increases with high expression of PepC… GDPD is important for low P adaptation); what is new in your results as compared to the literature? How do your results contribute to a general understanding of P nutrition in white lupin? Previous knowledge and your data should be better integrated.

A18: Thank you for the advice, we revised the discussion part extensively. We have removed the general descriptions, and discusses our findings in related to previous results, and provided what is new in our results clearly.

Q19: Suppl Table 3: Vigna instead of vigan;

A19: “*Vigan*” has been replaced by “*Vigna*”.

Q20: Suppl Table 6: Vigna instead of vigra

A20: “*Vigan*” has been replaced by “*Vigna*”.

Q21: Suppl Figure 13-19: it would be good to add letters showing the significantly different classes according to a t-test, above the bars.

A21: Thanks for your suggestion. The letters have been added above the error bars to indicate the significant difference according to Student’s t-test.

Comments from Reviewer #2:

Question #1: This revision corrects most of my comments relative to the first submission. I feel that this revision is much more solid. The analyses look appropriate and well conducted.

Answer 1: Thanks for your comments. We have further revised the manuscript (in blue).

Q2: line 21 (abstract): "White lupin is the legume crop that" --> "White lupin is a legume crop that" -- "a" instead of "the" because there are other lupin species (L. mutabilis at least) that are used as crops and have cluster roots and PUE.

A2: “the” has been replaced by “a”.

Q3: lines 26-28 (abstract): "and revealed the sub-genome dominance phenomenon, as well as the genic expression of the different sub-genomes vary in relation to their TE density." This sentence is not grammatical. It would be better as: "and find sub-genome dominance, with genic expression of the different sub-genomes varying in relation to their TE density."

A3: "and revealed the sub-genome dominance phenomenon, as well as the genic expression of the different sub-genomes vary in relation to their TE density." has been replaced by "and find sub-genome dominance, with genic expression of the different sub-genomes varying in relation to their TE density."

Q4: line 30: "four main pathways on high PUE of white lupin" --> "four main pathways involved in the high PUE of white lupin"

A4: "four main pathways on high PUE of white lupin" has been changed to “four main pathways for high PUE of white lupin”.

Q5: lines 63-64: "lupin will contributes" --> "lupin will contribute"

A5: “contributes” has been replaced by “contribute”.

Q6: line 102: "were uploaded to the NCBI database" -- would be good to add the project number (PRJNA592024) or accession here:

A6: The project number (PRJNA592024) has been mentioned.

Q7: line 118: "divergent ~39.97 million years" - does this refer to the divergence between Cicer and Phaseolus (I think so).

A7: We are sorry for the confusion, it’s between *L. albus* and *P. vulgaris*. We have revised corresponding sentences and added the species pairs on the divergence time to make it clear.

Q8: line 120: "divergent ~40.75 million years" - does this refer to the divergence between the two Arachis species? If so, this is incorrect. These species had a common ancestor around 2 million years ago (Bertioli et al., 2016)

A8: Sorry again, it’s not. Please see the response of **A7**.

Q9: line 121: "Two polyploidization events were found in these legume species" - this is too simple. It neglects to mention the important WGD that affects ALL papilionoid legumes, estimated to have occurred around 55 Mya and other, independent WGDs affecting other legumes (Cannon, McKain et al., 2015; Stai, Yadav et al., 2019). Another, recent WGD is seen in tetraploid Arachis. Other WGDs are seen in e.g. Mimosa and other lineages. It would be good to correct this in Figure 1a as well (adding a star at the base of the Papilionoid clade).

A9: Thanks very much for pointing out this, we agree with your comments. We have added the red star at the base of the Papilionoid clade as suggested, and revised the corresponding sentence to avoid confusions.

Q10: line 156: "white lupine" (extra "e")

A10: the extra “e” has been removed.

Comments from Reviewer #3:

Question #1: The manuscript "The genome evolution and low phosphorus adaptation in white lupin (*Lupinus albus*)" by Xu et al. has been carefully revised and my main concerns have been addressed.

Answer 1: Thanks for your comments. We have further revised the manuscript (in blue).

Minor points (in addition to the pdf):

Q2: Abstract

I suggest to put a little work in perfecting the abstract. The language is not incorrect, but considering the importance of the abstract, I think there is room for improvement.

A2: Thank you for your suggestion. The abstract has been carefully revised.

Q3: Results

Page 9, lines 225-229

"Genes annotated as "glycolysis", "tricarboxylic acid cycle", "lipid biosynthetic process", "ATPase activity" and different types of phosphate-related functions increased in L. albus compared to L. angustifolius (Supplementary Table 14). This indicates that the function pathways of these genes evolved in L. albus after it diverged from L. angustifolius."

It is incorrect to state that these pathways evolved after divergence, as these are ancient pathways. How about: "This indicates that over-retaining of genes in these pathways occured in L. albus after it diverged from L. angustifolius"

A3: Thanks for your suggestion.

"This indicates that the function pathways of these genes evolved in *L. albus* after it diverged from *L. angustifolius*." has been replaced by "This indicates that over-retaining of genes in these pathways occurred in *L. albus* after it diverged from *L. angustifolius*".

Q4: Discussion

Line 332-333: The structure of the discussion needs some work. The discussion states "four main biological pathways…" It then counts 3 pathways (not very clearly), and lacks the fourth.

A4: We are sorry for the confusion, we previously used "beside…" to start the fourth pathway. We have extensively rewritten the discussion part in the revised manuscript.

Q5: Page 13, lines 337-341

"Our results suggest that carbon fixation capacity increases with high expression of PEPC genes (Supplementary Table 24), which is important for root growth in response to P deficiency. Secondly, under the support of carbon fixation, cluster root formation can be promoted."

Is dark fixation really mainly important for root growth in response to low P? I always assumed its main purpose was to generate organic acids for excretion.

A5: Yes, we agree with you. The description has been rewritten according to your suggestion.

Q6: Materials and Methods

Line 559: Phosphoproteomic analysis is described in the Materials and Methods, but I can't find any mentioning in the results.

A6: Thanks very much for pointing out this. We have rewritten the result part and described our phoshoproteomic data in Line 324-327.

Comments in the attached-pdf MS

Q7: Line 22 "the".

A7: "the" has been replaced by "a".

Q8: Line 22 "was".

A8: "was" has been replaced by "has".

Q9: Line 25-28, this sentence is long and a little difficult to understand. I suggest you split it into 2 sentences, one that states what you have done, another what was revealed.

A9: We have revised the sentence as suggested.

Q10: Line 29 insert "through".

A10: "through" has been inserted.

Q11: Line 30 "on".

A11: “on” has been replaced by “for”.

Q12: Line 39 “form”.

A12: “form” has been replaced by “forms”.

Q13: Line 47 “N_2”

A13: “N_2” has been replaced by “dinitrogen (N_2) ”.

Q14: Line 48, citation.

A14: The citation has been added.

Q15: Line 52, citation.

A15: The citation has been added.

Q16: Line 77 “in situ”; italicize.

A16: “in situ” has been italicized in the line 78.

Q17: Line 83 “,” .

A17: “,” has been replaced by “;”.

Q18: Line 83 “whereas”.

A18: “whereas” has been deleted.

Q19: Line 84 “linking”

A19: “linking” has been replaced by “linkage”.

Q20: Line 147 “the”

A20: “the” has been replaced by “than”.

Q21: Line 152 “*A. ipaensis*”. spell out at first mentioning

A21: "*A. ipaensis*" has been replaced by "*Arachis ipaensis*".

Q22: Line 156 "lupine".

A22: "lupine" has been replaced by "lupin".

Q23: Line 176 "left".

A23: "left" has been replaced by "remaining".

Q24: Line 178 "orthologous".

A24: "orthologous" has been replaced by "orthologs".

Q25: Line 180 "that of ".

A25: "that of " has been deleted as suggested.

Q26: Line 188 "that of".

A26: "that of " has been deleted as suggested.

Q27: Line 204 "two steps".

A27: "two steps " has been replaced by "two-step".

Q28: Line 210 "have".

A28: "have" has been deleted as suggested.

Q29: Line 224 "My".

A29: "My" has been replaced by "Mya".

Q30: Line 288 "orthologue".

A30: "orthologue" has been replaced by "ortholog".

Q31: Line 288 "overexpressing".

A31: “overexpressing” has been replaced by “- overexpressing”.

Q32: Line 289 “*rhizogene*”.

A32: “*rhizogene*” has been replaced by “*rhizogenes*”.

Q33: Line 294 “involved”.

A33: “involved” has been changed into “is involved”.

Q34: Line 294 “P deficiency induced”.

A34: “P deficiency induced” has been replaced by “P deficiency-induced”.

Q35: Line 339, is carbon fixation really important for root growth? I always assumed the purpose of dark fixation was to generate enough citrate and malate for excretion?

A35: We have removed and revised some sentences on the relationship between carbon fixation and root growth.

Q36: Line 340, does carbon fixation really support cluster root formation, or is it rather that cluster root function involves carbon fixation?

A36: We agree with your comments, and revised corresponding part.

Q37: Line 341 “new paragraph”.

A37: Thanks, we have revised the discussion part extensively.

Q38: Line 351 “is this the fourth pathway? If so, state this. Also, make a new paragraph for each of the 4 pathways”.

A38: Thanks, we have revised the discussion part extensively.

Q39: Line 367, “will”.

A39: “will” has been replaced by “may”.

Q40: Line 382, “as”.

A40: “as” has been replaced by “for”.

REVIEWERS' COMMENTS:

Reviewer #1 (Remarks to the Author):

The revised manuscript by Xu et al. on “The genome evolution and low phosphorus adaptation in white lupin” has been substantially improved and authors have addressed my major concerns. I suggest in the attached file some additional minor revisions. The main revision needed is to cite properly the white lupin genome from Hufnagel et al.

Reviewer #3 (Remarks to the Author):

The manuscript “The genome evolution and low phosphorus adaptation in white lupin (Lupinus albus)” by Xu et al. has been carefully revised a second time and my concerns have been addressed.

The paper reads well and a rather complicated topic is presented in a clear and interesting manner; I believe it is ready for publication!

There are a couple of very minor points that I addressed directly in the attached pdf.

Point by point responses to reviewers' comments

Comments from Reviewer #1:

Question #1: The revised manuscript by Xu et al. on "The genome evolution and low phosphorus adaptation in white lupin" has been substantially improved and authors have addressed my major concerns. I suggest in the attached file some additional minor revisions. **The main revision needed is to cite properly the white lupin genome from Hufnagel *et al*.**

Answer 1: Thanks! We have compared our genome with the currently released genome of Hufnagel *et al*., and cited their work as suggested.

Comments from R1-PDF:

Q2: Line 39: "P is often present in unavailable forms" ---> "P is often present in soils in unavailable forms".

A2: "P is often present in unavailable forms" has been replaced by "P is often present in soils in unavailable forms".

Q3: Line 44: remove "been".

A3: "been" has been removed.

Q4: Line 52-53: This is not correct. A white lupin genome has been already published is publicly available at https://www.whitelupin.fr/. Please refer to it!

A4: We have changed the sentence to **"**However, the genome evolution and low-P adaptation in white lupin is unclear". We also compared with and cited the currently released white lupin genome.

Q5: Line 56: "but it does not show low-P adaptation" move to line 66-67.

A5: Thanks, this sentence has been moved to line 66-67.

Q6: Line 57: "Meso hexaploidization is not a common term. Remove meso or specify what it means and why it applies in the case of white lupin."

A6: "Meso" has been removed.

Q7: Line 109: Same remark as line 57.

A7: “Meso” has been removed.

Q8: Line 141: “The diploid ancestral genome of white lupin had nine chromosomes.” ---> “The diploid ancestral genome of white lupin probably had nine chromosomes.”

A8: “The diploid ancestral genome of white lupin had nine chromosomes.” has been replaced by “The diploid ancestral genome of white lupin probably had nine chromosomes”.

Q9: Line 212: “P-use-efficiency” ---> “P-use efficiency”, correct here and elsewhere.

A9: “P-use-efficiency” has been replaced by “P-use efficiency”.

Q10: Line 250: “low-phosphorus-adaptive” ---> “low-P adaptive”. Ok for me but then, why put low-phosphorus below instead of low-P? if you need to specific what is low-P do it the first time you use it.

A10: Thanks for your suggestion. “low-phosphorus-adaptive” has been replaced by “low-P adaptive”.

Q11: Line 276: “…composition to remobilize…” ---> “…composition most probably to remobilize…”

A11: “…composition to remobilize…” has been replaced by “…composition most probably to remobilize…”.

Q12: Line 329: “These results together strongly suggest that…” ---> “Altogether, these results strongly suggest that…”

A12: “These results together strongly suggest that…” has been replaced by “Altogether, these results strongly suggest that…”.

Q13: Line 339: remove “from the WGT”.

A13: “from the WGT” has been deleted.

Q14: Line 339: remove “the”.

A14: “the” has been deleted.

Q15: Line 340: remove “phenomenon”.

A15: “phenomenon” has been deleted.

Q16: Line 342-343: “and protein phosphorylation might be involved in the regulation of PUE genes (Supplementary Table 28).” should be placed after line 347.

A16: We have revised the sentence as suggested.

Q17: Line 344-345: This is already described from line 357 to 369.

A17: This sentence has been deleted.

Q18: Line 348: “likely have” ---> “have likely”.

A18: “likely have” has been replaced by “have likely”.

Comments from Reviewer #3:

Q1: The manuscript "The genome evolution and low phosphorus adaptation in white lupin (*Lupinus albus*)" by Xu et al. has been carefully revised a second time and my concerns have been addressed.

The paper reads well and a rather complicated topic is presented in a clear and interesting manner; I believe it is ready for publication!

There are a couple of very minor points that I addressed directly in the attached pdf.

A1: Thank you for your comments. We have revised all the points that you suggested, please see the detail in the manuscript (in blue).

Comments from R3-PDF:

Q2: Line 52-53: this sounds a bit awkward, but I understand that you will discuss if/how the other white lupin genome study should be cited. If it is not to be cited, I suggest: the lack of a genomic sequence for white lupin...

A2: The previous description has been rephased by "However, the genome evolution and low-P adaptation in white lupin is unclear".

Q3: Line 137: Insert "-".

A3: "-" has been inserted.

Q4: Line 190: remove "the".

A4: "the" has been removed.

Q5: Line 215: remove "as".

A5: "as" has been removed.

Q6: Line 239: delete "the".

A6: "the" has been deleted.

Q7: Line 251-252 and line 271: Insert "-".

A7: "-" has been inserted.

Q8: Line 311-312: delete "One role of EDTA is to inhibit the activity of".

A8: This sentence has been changed into "The activity of AAA proteins (ATPases associated with diverse cellular activities) can be inhibited by EDTA".

Q9: Line 316: delete "the".
A9: "the" has been deleted.

Q10: Line 322: replace "mRNA-seq" by "RNA-seq".
A10: We have done this.

Q11: Line 328: Insert "-".
A11: "-" has been inserted.

Q12: Line 344-345: "as potential genes that may function…" ---> "as potentially function…"
A12: "as potential genes that may function…" has been replaced by "as potentially function…".

Q13: Line 346-347: "These results together increased our knowledge on the genome evolution, as well as provide clues to the low-P adaptation from the genome perspective of white lupin." ---> "Taken together, these results increased our knowledge on the genome evolution, as well as provide clues to the low-P adaptation of white lupin from the genome perspective."
A13: Thanks, we have revised the sentence as suggested.

Q14: Line 348: "Four main biological pathways likely have co-evolved for the adaptations of white lupin to low levels of P (Supplementary Figure 20)." ---> "Four main biological pathways have likely co-evolved as adaptations of white lupin to low levels of P (Supplementary Figure 20)."
A14: The sentence has been revised as suggested.

Q15: Line 357: delete "for the cluster root development".
A15: "for the cluster root development" has been deleted.

Q16: Line 358: remove "the".

A16: “the” has been removed.

Q17: Line 373: replace “by” by “for”.
A17: “by” has been replaced by “for”.

Q18: Line 385: remove “these”.
A18: “these” has been removed.

Q19: Line 391: replace “the low-P adaptation of white lupin” by “white lupin's adaptation to low P”.
A19: “the low-P adaptation of white lupin” has been replaced by “white lupin's adaptation to low P”.
